# Integrated analyses to reconstruct microRNA-mediated regulatory networks in mouse liver using high-throughput profiling

**DOI:** 10.1186/1471-2164-16-S2-S12

**Published:** 2015-01-21

**Authors:** Sheng-Da Hsu, Hsi-Yuan Huang, Chih-Hung Chou, Yi-Ming Sun, Ming-Ta Hsu, Ann-Ping Tsou

**Affiliations:** 1 Institute of Bioinformatics and Systems Biology, National Chiao Tung University, Hsin-Chu 300, Taiwan; 2 Center for Bioinformatics Research, National Chiao Tung University, Hsin-Chu 300, Taiwan; 3 Department of Biological Science and Technology, National Chiao Tung University, Hsin-Chu 300, Taiwan; 4 Department of Biotechnology and Laboratory Science in Medicine, National Yang-Ming University, Taipei, 112, Taiwan; 5 Genome Research Center, National Yang-Ming University, Taipei, 112, Taiwan

## Abstract

**Background:**

MicroRNAs (miRNAs) simultaneously target many transcripts through partial complementarity binding, and have emerged as a key type of post-transcriptional regulator for gene expression. How miRNA accomplishes its pleiotropic effects largely depends on its expression and its target repertoire. Previous studies discovered thousands of miRNAs and numerous miRNA target genes mainly through computation and prediction methods which produced high rates of false positive prediction. The development of Argonaute cross-linked immunoprecipitation coupled with high-throughput sequencing (CLIP-Seq) provides a system to effectively determine miRNA target genes. Likewise, the accuracy of dissecting the transcriptional regulation of miRNA genes has been greatly improved by chromatin immunoprecipitation of the transcription factors coupled with sequencing (ChIP-Seq). Elucidation of the miRNA target repertoire will provide an in-depth understanding of the functional roles of microRNA pathways. To reliably reconstruct a miRNA-mediated regulatory network, we established a computational framework using publicly available, sequence-based transcription factor-miRNA databases, including ChIPBase and TransmiR for the TF-miRNA interactions, along with miRNA-target databases, including miRTarBase, TarBase and starBase, for the miRNA-target interactions. We applied the computational framework to elucidate the miRNA-mediated regulatory network in the *Mir122a^-/- ^*mouse model, which has an altered transcriptome and progressive liver disease.

**Results:**

We applied our computational framework to the expression profiles of miRNA/mRNA of *Mir122a^-/- ^*mutant mice and wild-type mice. The miRNA-mediated network involves 40 curated TFs contributing to the aberrant expression of 65 miRNAs and 723 curated miRNA target genes, of which 56% was found in the differentially-expressed genes of *Mir122a^--^*mice. Hence, the regulatory network disclosed previously-known and also many previously-unidentified miRNA-mediated regulations in mutant mice. Moreover, we demonstrate that loss of imprinting at the chromosome 12qF1 region is associated with miRNA overexpression in human hepatocellular carcinoma and stem cells, suggesting initiation of precancerous changes in young mice deficient in miR-122. A group of 9 miRNAs was found to share miR-122 target genes, indicating synergy between miRNAs and target genes by way of multiplicity and cooperativity.

**Conclusions:**

The study provides significant insight into miRNA-mediated regulatory networks. Based on experimentally verified data, this network is highly reliable and effective in revealing previously-undetermined disease-associated molecular mechanisms. This computational framework can be applied to explore the significant TF-miRNA-miRNA target interactions in any complex biological systems with high degrees of confidence.

## Background

Since its first discovery in *Caenorhabditis elegans *[1-3], microRNA (miRNA) has been seen as a key regulator of gene expression [4]. miRNAs are RNA molecules ranging in size from 21 to 23 nucleotides that down-regulate genes by guiding Argonaute (AGO) proteins to form miRNA-induced silencing complexes (miRISC) leading to translational repression or mRNA degradation [5]. Currently, there are 2588 mature human miRNAs (1881 precursors) and 1915 mature mouse miRNAs (1193 precursors) (miRBase 21, http://www.mirbase.org/). miRNAs are regulated in a developmental stage-and tissue-specific fashion [6,7] and are known to participate in diverse biological functions [8-12]. The dysregulation of miRNAs has been correlated with the pathogenesis of various human diseases such as cancer [13]. Cumulative evidence has shown that, similar to protein-coding genes, miRNA genes primarily fall under transcriptional and epigenetic regulation [14]. With the advent of bioinformatics and high-throughput technology, several TF-miRNA regulatory network databases have been constructed. CircuitsDB performed human and mouse TF-miRNA Feed-Forward regulatory Loops (FFLs) by predicting the TF promoter and miRNA-target region [15]. Wang *et al*. [14] curated studies containing approximate 5000 transcription factor-miRNA regulations to create the TransmiR database. Yang *et al*. [16] integrated the high throughput ChIP-Seq datasets to provide a web-based tool called ChIPBase to help detect transcription factor binding sites (TFBSs) for non-coding RNAs including miRNAs.

The power of miRNA-target interactions (MTIs) lies in their multiplicity and cooperativity. For nearly a decade, MTIs were mostly obtained by way of computation and prediction. Numerous bioinformatics tools were generated that were based primarily on miRNA seed region complementarity with genes, free energy of miRNA-RNA duplexes, and conservation of target sites across the species [17]. Widely used MTIs prediction tools include TargetScan [18], miRanda [17], PicTar [19] and many others. The pace of identification of relevant MTIs is rather slow, mostly due to the high rate of false-positive predictions of miRNA binding sites. Rapid progress in high throughput screening technologies such as microarrays, small RNA sequencing (sRNA-Seq) and RNA sequencing (RNA-Seq) can expedite MTI prediction. The most exciting breakthrough in recent years, however, is a method which allows for the direct collection of AGO-miRNA-mRNA complexes by Argonaute cross-linked immunoprecipitation coupled with high-throughput sequencing. This experimental approach has advanced our understanding of MTI interactions from prediction-based knowledge to a level that is empirically-and genome wide-based. Representative technologies include CLIP-Seq (cross-linking and immunoprecipitation sequencing) [20] and CLASH sequencing (crosslinking, ligation, and sequencing of hybrids) [21] which can directly detect miRNA target sites. Meanwhile, development of the web servers, dChip-GemiNI [22], MAGIA [23,24], and mirConnX [25] provides additional tools for constructing TF-miRNA regulatory networks through integrating gene and miRNA expression profiles with target prediction. As a proof-of-concept demonstration, the high-throughput techniques of CLIP-Seq for miRNA-target interaction and ChIP-Seq for TF studies were found to accelerate discovery of TF-miRNA-gene regulatory networks in human pancreatic cancer early this year [26].

While experimental-based databases have become more popular for building gene regulatory networks, most reconstructed TF-miRNA-gene regulation networks are still computation/prediction-based. We expect an analysis platform integrating high-throughput datasets from ChIP-Seq for TF-miRNA network and CLIP-Seq and CLASH for miRNA-Target networks could be used to effectively explore significant TF-miRNA-MTIs interactions in any complex biological system with improved confidence.

We applied this integrated analysis platform to the *Mir122a *knockout mouse (*Mir122a^-/-^*) model of liver disease. MicroRNA-122 (miR-122) is a highly abundant, developmental-regulated, liver-specific miRNA. It plays a pivotal role in maintaining lipid metabolism homeostasis [27,28] and tumor suppression in the liver [29,30]. *Mir122a *knockout mice (*Mir122a^-/-^*) develop temporally-controlled steatohepatitis, fibrosis and hepatocellular carcinoma (HCC), a path similar to the disease progression in humans [31,32]. A striking feature of this mouse model is that miR-122a profoundly modulates the liver transcriptome. The pathway disturbances that might drive cancer initiation and progression are found in young *Mir122a^-/- ^*mice (2-months old). To date we have detected the expression of 79 experimentally-verified miR-122 targets, which represents only 8.9% (79/886) of the differentially expressed genes (DEGs) [32] in *Mir122a^-/- ^*livers. Since miR-122 represents ~70% of the liver miRNAs, the imbalance of the miRNA homeostasis in *Mir122a^-/- ^*liver can give rise to liver damage. A systematic, genome-wide investigation of the miRNA-mediated regulatory networks will provide important insights into the molecular mechanisms by which miR-122 modulates liver transcriptome and disease.

## Results

**Figure 1:**
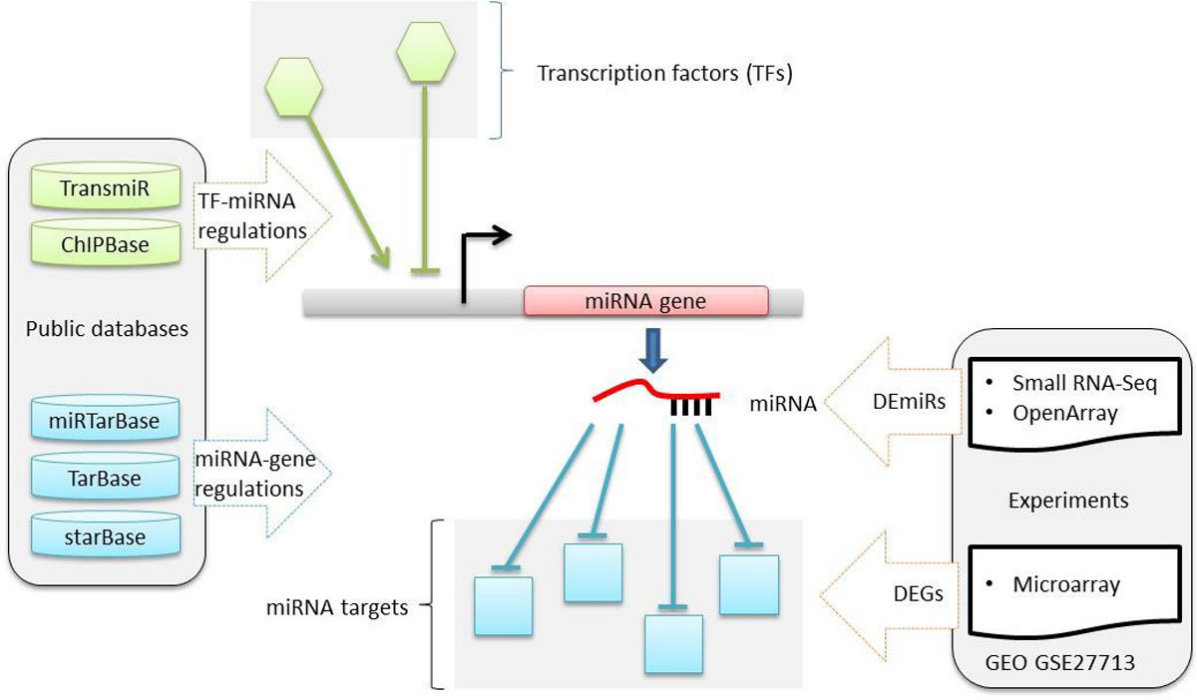
**Overview of the computational framework for reconstructing the miRNA-mediated regulatory network**.

We reconstructed the miRNA-mediated regulatory network in mouse livers using high-throughput expression profiles. The overview is shown in Figure 1. First, we identified DEGs by retrieving the multiple gene microarray datasets from GEO (GSE27713). High-confidence differentially expressed miRNAs (DEmiRs) were derived from the intersection of two miRNA expression profiles (OpenArray and small RNA-Seq). Second, we mapped the DEGs and DEmiRs to the curated TF-miRNA regulatory network as active nodes and removed the other non-mapping nodes. Finally, we reconstructed the potential miRNA-mediated regulatory network in *Mir122a^-/- ^*mouse livers.

### Differential expression of miRNAs in *Mir122a^-/- ^*mice

To obtain a list of high confidence DEmiRs in *Mir122a^-/- ^*mouse livers, we subjected the same pair of wild-type (WT) and *Mir122a^-/- ^*mouse liver samples to small RNA sequencing and OpenArray assay for miRNA profiling. The profiling results from these two entirely different platforms showed a positive correlation (R = 0.7). For each profiling technology, we selected DEmiRs (see the Method section). Considering that different platforms have different coverage for all mouse miRNAs, we focus on the DEmiRs presented by both small RNA-Seq and OpenArray assay. We identified 48 up-regulated miRNAs (UPmiRs) and 17 down-regulated miRNAs (DNmiRs) in *Mir122a^-/- ^*mouse livers. Among the UPmiRs, expression of 41 miRNAs (85%) was highly elevated while 7 others showed moderate increase in expression (Table 1). We studied the chromosomal locations and found that, remarkably, the group of 41 highly expressed UPmiRs is located in the imprinted Dlk1-Dio3 gene cluster on the mouse chromosome 12qF1 region (Chr12qF1). In contrast, only 1 DNmiR (6%) was located in the Chr12qF1 region. No preferred chromosomal locations were assigned to those moderately increased UPmiRs and other DNmiRs. We speculate that the promoter regions of Chr12qF1-DEmiRs operate under a similar mode of transcription regulation. We further investigated the TF-miRNA regulation to support our speculation.

**Table 1:** List of differentially expressed miRNAs common to both OpenArray analysis and miRNA-Seq.

Category	miRNA	miRNA loci	miRNA-Seq	OpenArray
	miR-429-3p	chr4:156053905-156053987:-	2.01	3.56E+00
	
	miR-200a-3p	chr4:156054896-156054985:-	2.50	2.25E+00
	
	miR-200b-3p	chr4:156055681-156055750:-	3.53	4.25E+00
	
	miR-200c-3p	chr6:124718322-124718390:-	1.86	2.88E+00
	
	miR-182-5p	chr6:30165918-30165992:-	2.12	2.41E+00
	
	miR-326-3p	chr7:99552269-99552363:+	2.07	1.83E+00
	
	miR-199a-5p	chr9:21496495-21496564:-	3.39	1.10E+01
	
	miR-673-3p	chr12: 109571990-109572080:+	34.09	3.55E+04
	
	miR-337-3p	chr12: 109585789-109585885:+	22.14	9.67E+00
	
	miR-337-5p	chr12: 109585789-109585885:+	59.88	2.20E+01
	
	miR-540-3p	chr12: 109586080-109586146:+	26.89	1.04E+04
	
	miR-431-5p	chr12: 109590447-109590537:+	34.02	5.96E+05
	
	miR-127-3p	chr12: 109592846-109592915:+	61.60	1.59E+01
	
	miR-127-5p	chr12: 109592846-109592915:+	38.92	3.07E+01
	
	miR-434-3p	chr12: 109594506-109594599:+	68.55	2.98E+01
	
	miR-434-5p	chr12: 109594506-109594599:+	49.12	2.35E+01
	
	miR-136-3p	chr12:109595327-109595388:+	55.82	1.29E+01
	
	miR-136-5p	chr12:109595327-109595388:+	42.94	2.49E+00
	
	miR-379-5p	chr12:109709060-109709125:+	46.48	1.74E+01
	
	miR-379-3p	chr12:109709060-109709125:+	62.39	7.18E+00
	
	miR-411-3p	chr12:109710175-109710256:+	64.08	1.16E+01
	
	miR-411-5p	chr12:109710175-109710256:+	56.88	2.51E+01
	
Up-regulated	miR-299a-5p	chr12:109710638-109710700:+	34.89	6.81E+02
	
	miR-329-3p	chr12:109713481-109713577:+	19.84	1.05E+04
	
	miR-494-3p	chr12:109715318-109715402:+	16.36	1.35E+03
	
	miR-1193-3p	chr12:109715671-109715791:+	33.38	1.07E+01
	
	miR-543-3p	chr12:109717258-109717333:+	28.07	5.30E+00
	
	miR-495-3p	chr12:109718754-109718816:+	31.35	2.03E+01
	
	miR-376c-3p	chr12:109722718-109722803:+	43.62	1.85E+01
	
	miR-376b-3p	chr12:109723458-109723539:+	64.47	4.21E+01
	
	miR-376b-5p	chr12:109723458-109723539:+	56.26	2.81E+01
	
	miR-376a-3p	chr12:109723781-109723848:+	12.12	2.07E+04
	
	miR-300-3p	chr12:109724313-109724391:+	48.31	2.87E+03
	
	miR-381-3p	chr12:109726822-109726896:+	59.30	2.33E+03
	
	miR-382-5p	chr12:109733771-109733846:+	26.07	4.62E+00
	
	miR-382-3p	chr12:109733771-109733846:+	16.26	4.37E+03
	
	miR-134-5p	chr12:109734139-109734209:+	41.55	3.29E+01
	
	miR-485-5p	chr12:109734902-109734974:+	10.71	1.18E+05
	
	miR-485-3p	chr12:109734902-109734974:+	63.49	9.91E+01
	
	miR-154-3p	chr12:109738433-109738498:+	63.36	8.38E+01
	
	miR-154-5p	chr12:109738433-109738498:+	57.20	4.12E+03
	
	miR-496a-3p	chr12:109739119-109739197:+	48.48	5.39E+03
	
	miR-541-5p	chr12:109742409-109742498:+	48.98	4.90E+01
	
	miR-409-5p	chr12:109743158-109743236:+	16.22	7.26E+03
	
	miR-409-3p	chr12:109743158-109743236:+	58.62	2.91E+01
	
	miR-369-3p	chr12:109743418-109743496:+	19.52	1.03E+04
	
	miR-369-5p	chr12:109743418-109743496:+	7.92	2.19E+03
	
	miR-410-3p	chr12:109743715-109743795:+	51.74	9.52E+00

Down-regulated	miR-455-3p	chr4:63256851-63256932:+	0.64	4.35E-01
	
	miR-455-5p	chr4:63256851-63256932:+	0.66	3.85E-01
	
	miR-31-3p	chr4:88910557-88910662:-	0.40	9.54E-02
	
	miR-31-5p	chr4:88910557-88910662:-	0.25	8.27E-02
	
	miR-93-5p	chr5:138165523-138165610:-	0.58	9.70E-04
	
	miR-339-3p	chr5:139369650-139369745:-	0.57	2.59E-01
	
	miR-335-5p	chr6:30741299-30741396:+	0.41	3.99E-01
	
	miR-486b-5p	chr8: 23142573-23142662:-	0.50	3.09E-01
	
	miR-144-3p	chr11:78073005-78073070:+	0.46	1.83E-01
	
	miR-451a	chr11:78073170-78073241:+	0.35	7.81E-03
	
	miR-345-5p	chr12:108836973-108837068:+	0.52	3.19E-01
	
	miR-17-5p	chr14:115043671-115043754:+	0.55	2.16E-07
	
	miR-19a-3p	chr14:115044000-115044081:+	0.35	4.21E-01
	
	miR-484	chr16:14159626-14159692:+	0.59	6.05E-01
	
	miR-802-5p	chr16:93369720-93369816:+	0.17	7.01E-02
	
	miR-145a-5p	chr18:61647825-61647894:-	0.56	1.95E-01
	
	miR-322-5p	chrX:53054255-53054349:-	0.61	4.79E-01

There are 48 up-regulated and 17 down-regulated miRNAs in 2-month-old male *Mir122a^-/- ^*(KO) and WT mice. The expression ratio between the *mir-122^-/- ^*and WT (KO/WT) is shown.

### Potential TF-miRNA regulatory networks in *Mir122a^-/- ^*mice

**Figure 2:**
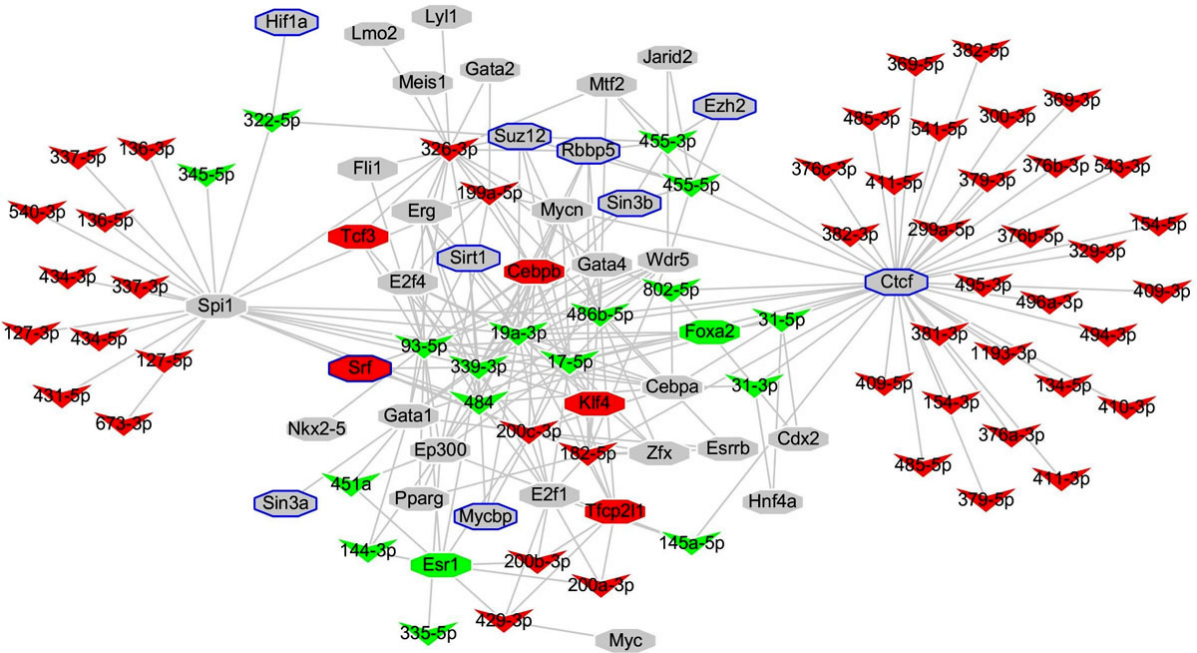
**TF-miRNA regulatory network**. A total of 240 TF-miRNA interactions involving 40 TFs and 65 DEmiRs were collected for this network, with transcription factor Spi1 and Ctcf binding directly to the promoters of numerous miRNAs. Most of the UPmiRs (41 miRNAs) regulated by Ctcf and Spi1 are located in Chr12qF1. The octagonal nodes represent the transcription factors (TF) and the V-shaped nodes correspond to the miRNAs, while the nodes marked by the blue border represent the miR-122 targets. The up-regulated miRNAs/TFs are marked in red; down-regulated miRNAs/TFs are marked in green; TFs showing no differential expression are marked in grey.

To understand whether these DEmiRs are transcriptionally regulated by specific transcription factors in *Mir122a^-/- ^*mice, we reconstructed the TF-miRNA regulatory networks by integrating two experimentally verified resources, ChIPBase [16] and TransmiR [14]. ChIPBase is a comprehensive resource which includes annotated 7306 TF-miRNA relationships from 119 mouse ChIP experiments targeting 73 TFs. We identified a total of 240 TF-miRNA interactions involving 40 TFs and 65 DEmiRs. The TF-DEmiRs regulatory network in the *Mir122a^-/- ^*hepatocyte is presented in Figure 2 and detailed TF-DEmiRs interactions are listed in Tables S2.1 and S2.2. The results showed that, similar to the majority of the pol II genes, one miRNA can be regulated by different TFs (Table S2.1) and one specific TF can modulate the expression of multiple target miRNAs (Table S2.2). Ten of the 40 curated TFs, potentially regulating 46 miRNAs, are verified as miR-122 target genes. CTCF [33] is a miR-122 target gene found in the human HCC cell line, while Hif1a [34] is a recently confirmed miR-122a target in mouse hepatocytes. Although the target relationship of miR-122 with EZH2, MYCBP, RBBP5, SIN3A, SIN3B, SIRT1, SRF and SUZ12 has not been studied in mouse liver but it has been identified in starBase with human samples. The fact that many target genes of miR-122 are common to both mouse and human it is highly likely that EZH2, MYCBP, RBBP5, SIN3A, SIN3B, SIRT1, SRF and SUZ12 are mouse miR-122a target genes. This result suggests that miR-122 can potentially modulate the expression of 46 miRNAs via its target transcription factors.

The appearance of higher numbers of connections in some hub-TFs and hub-DEmiRs suggests that these hub-TFs may play an essential role in the regulation of multiple DEmiRs, and that the hub-DEmiRs could potentially be regulated by multiple TFs in a combinatorial mode. For example, spleen focus forming virus (SFFV) proviral integration oncogene (Spi1) and CCCTC-binding factor (Ctcf) are the top two hub-TFs (Figure 2). The expression level of both Spi1 and Ctcf is moderately increased (1.3-1.4 fold) in *Mir122a^-/- ^*mice. Ctcf can perhaps regulate 42 DEmiRs including 32 UPmiRs and 10 DNmiRs. Most of the Ctcf-UPmiRs (94%) are located in Chr12qF1 locus. Spi1 could potentially regulate 23 DEmiRs (15 UPmiRs with 12 miRNAs in Chr12qF1 and 8 DNmiRs). Our analysis also suggests that 6 DEmiRs, miR-17-5p, miR-486b-5p, miR-19a-3p, miR-484, miR-199a-5p and miR-182-5p, are likely to be co-regulated by both Ctcf and Spi1. Further experimentation is needed to support these initial findings. It is interesting to note that Spi1-UPmiRs and Ctcf-UPmiRs are respectively clustered within different regions of Chr12qF1, namely the *Meg3-Rian *region and *Mirg *region.

### Network analysis of curated miRNA target genes

**Figure 3:**
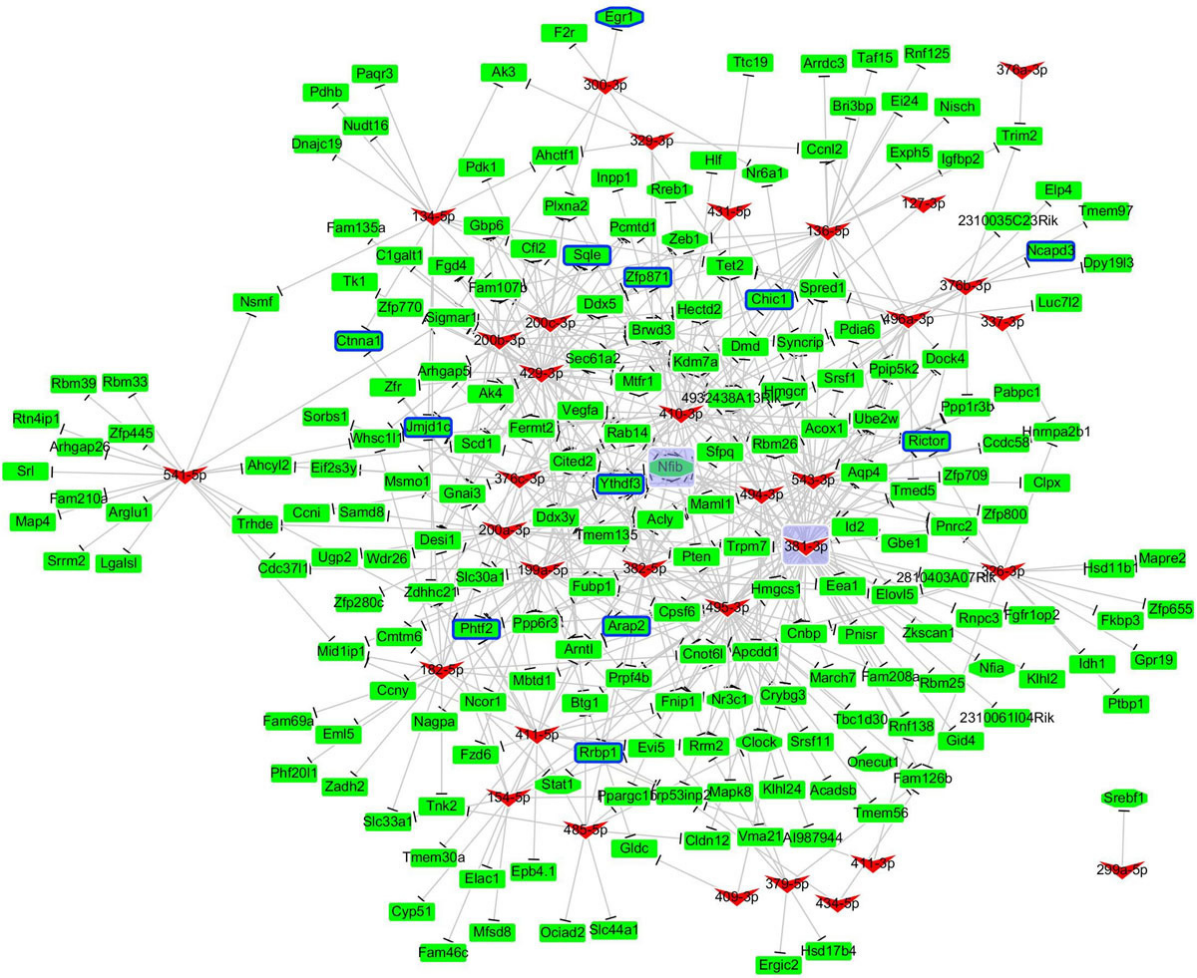
**UPmiRs regulatory network**. This network involves 536 interactions between 33 UPmiRs and 204 DEGs. Fifteen of the 48 UPmiRs did not have known validated targets (Table 2). The regulator hub-UPmiR is miR-381-3p which has 45 target genes (Table S3.1). Down-regulated *Nfib *being targeted by 12 UPmiRs (Table S3.2) is the miRNA target hub. The background of hub-miRNAs/genes is marked in purple. The V-shaped nodes correspond to the miRNAs, while the rectangle-shaped nodes are the target genes. The up-regulated miRNAs/genes are marked in red; down-regulated miRNAs/genes are marked in green. Regulatory effects are illustrated as blunt ends (⊥) between miRNAs and genes.

**Figure 4:**
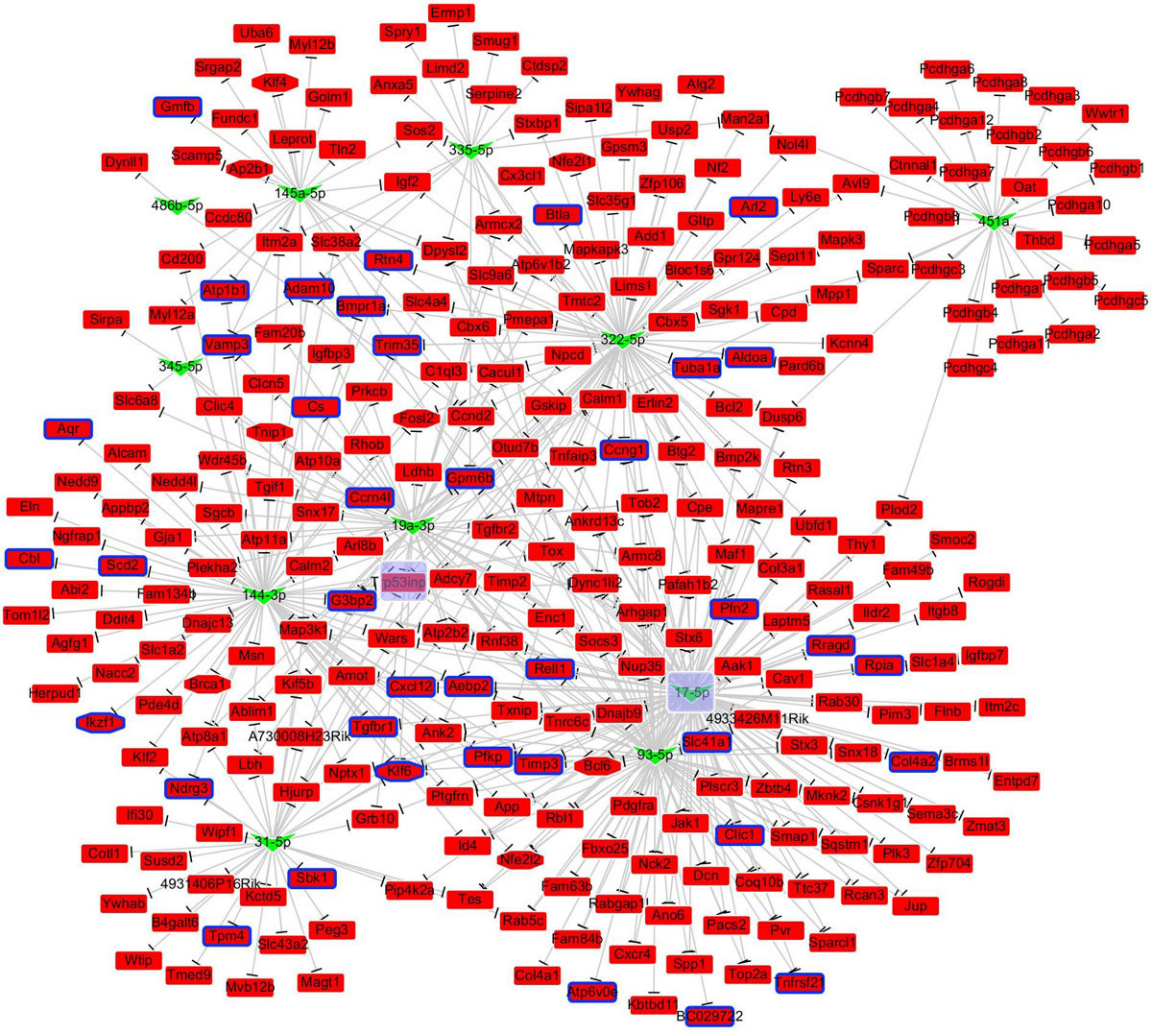
**DNmiRs regulatory network**. This network involves 531 interactions between 11 DNmiRs and 295 DEGs. Six of the 17 DNmiRs did not have known validated targets (Table 2). The regulator hub-DNmiR is miR-17-5p which has 115 target genes (Table S3.1). Up-regulated *Trp53inp1 *being targeted by 7 DNmiRs (Table S3.2) is the miRNA target hub. The background of hub-miRNAs/genes is marked in purple. The V-shaped nodes correspond to the miRNAs, while the rectangle-shaped nodes are the target genes, the nodes marked by the blue border represent the co-miR-122 targets, and the octagonal nodes represent the transcription factors (TF). The up-regulated miRNAs/genes are marked in red; down-regulated miRNAs/genes are marked in green. Regulatory effects are illustrated as blunt ends (⊥) between miRNAs and genes.

Since 21 DEmiRs have no known validated targets (Table S3.1), we collected the interactions of 44 DEmiRs and 2,315 DEGs to establish the miRNA-target regulatory network. Table 2 shows detailed statistics of the curated MTIs. A total of 1,067 interactions between the 44 DEmiRs and 723 curated target genes were obtained. There are 460 up-regulated and 263 down-regulated genes in the group of 723 curated targets. A total of 499 targets (295 up-regulated and 204 down-regulated) are present in the group of 886 DEGs found in 2-month old *Mir122a^-/- ^*mice livers [32]. We were surprised to find that 56% (499/886) of DEGs in *Mir122a^-/- ^*mice livers are likely modulated by dysregulated miRNAs. Figures 3 and 4 respectively present the UPmiRs-regulatory network (536 interactions between the 33 UPmiRs and the 204 DEGs) and the DNmiRs-regulatory network (531 interactions between the 11 DNmiRs and 295 DEGs). The regulator hub-DEmiRs in the two networks are miR-381-3p and miR-17-5p, respectively. Moreover, down-regulated *Nfib *and up-regulated *Trp53inp1 *are the miRNA target hubs (Table S3.2).

**Table 2:** Statistics for curated miRNA-target interactions.

	miRNA	Number of curated targets	Number of DE miRNA target genes in *Mir122a^-/- ^*mice
	miR-429-3p	438	39
	
	miR-200a-3p	394	25
	
	miR-200b-3p	410	25
	
	miR-200c-3p	382	26
	
	miR-182-5p	379	18
	
	miR-326-3p	175	16
	
	miR-199a-5p	249	24
	
	miR-673-3p	0	0
	
	miR-337-3p	36	2
	
	miR-337-5p	0	0
	
	miR-540-3p	0	0
	
	miR-431-5p	178	5
	
	miR-127-3p	39	1
	
	miR-127-5p	1	0
	
	miR-434-3p	7	0
	
	miR-434-5p	23	2
	
	miR-136-3p	0	0
	
	miR-136-5p	501	24
	
	miR-379-5p	138	6
	
	miR-379-3p	0	0
	
	miR-411-3p	26	2
	
	miR-411-5p	140	15
	
	miR-299a-5p	18	1
	
Up-regulated	miR-329-3p	329	5
	
	miR-494-3p	427	26
	
	miR-1193-3p	0	0
	
	miR-543-3p	480	36
	
	miR-495-3p	644	40
	
	miR-376c-3p	337	18
	
	miR-376b-3p	227	10
	
	miR-376b-5p	0	0
	
	miR-376a-3p	11	1
	
	miR-300-3p	40	6
	
	miR-381-3p	606	45
	
	miR-382-5p	296	20
	
	miR-382-3p	1	0
	
	miR-134-5p	203	14
	
	miR-485-5p	140	9
	
	miR-485-3p	0	0
	
	miR-154-3p	0	0
	
	miR-154-5p	226	11
	
	miR-496a-3p	218	13
	
	miR-541-5p	298	20
	
	miR-409-5p	0	0
	
	miR-409-3p	29	2
	
	miR-369-3p	0	0
	
	miR-369-5p	0	0
	
	miR-410-3p	524	29

Down-regulated	miR-455-3p	3	0
	
	miR-455-5p	0	0
	
	miR-31-3p	0	0
	
	miR-31-5p	334	36
	
	miR-93-5p	705	83
	
	miR-339-3p	0	0
	
	miR-335-5p	223	19
	
	miR-486b-5p	28	2
	
	miR-144-3p	709	72
	
	miR-451a	108	31
	
	miR-345-5p	18	5
	
	miR-17-5p	992	115
	
	miR-19a-3p	602	62
	
	miR-484	0	0
	
	miR-802-5p	0	0
	
	miR-145a-5p	262	26
	
	miR-322-5p	817	80

Total	65 DemiRs^a^	4,220^b*^	499^c,*^

^a ^includes 48 up-regulated miRNAs and 17 down-regulated miRNAs

^b ^includes 460 up-regulated target genes and 263 down-regulated target genes, for a total of 723 genes.

^c ^includes 295 up-regulated genes and 204 down-regulated genes of the 886 differentially-expressed genes found in 2-month old *Mir122a-/- *mouse livers [32].

* indicates the unique gene count

The complex interplay of miRNAs and target genes is accomplished through multiplicity and cooperativity. We identified 9 DEmiRs that can simultaneously regulate 39 miR-122 targets (Table S4.1, S4.2). *A priori*., we reasoned that in *Mir122a^-/- ^*livers, elevated expression of Klf6, a miR-122 target gene [32], is a simple response to the loss of miR-122a; nonetheless, elevated expression of Klf6 is likely to be the sum of a cascade of events involving the loss of miR-122a and the down regulation of 5 other DNmiRs (miR-17-5, -31, 19a, 93-5p and 144-3p). Our analysis supports the conclusion that miRNAs and transcription factors are two major classes of gene regulators using similar control principles, namely one-to-many and many-to-one relationships between the regulator and the target genes [17].

**Figure 5:**
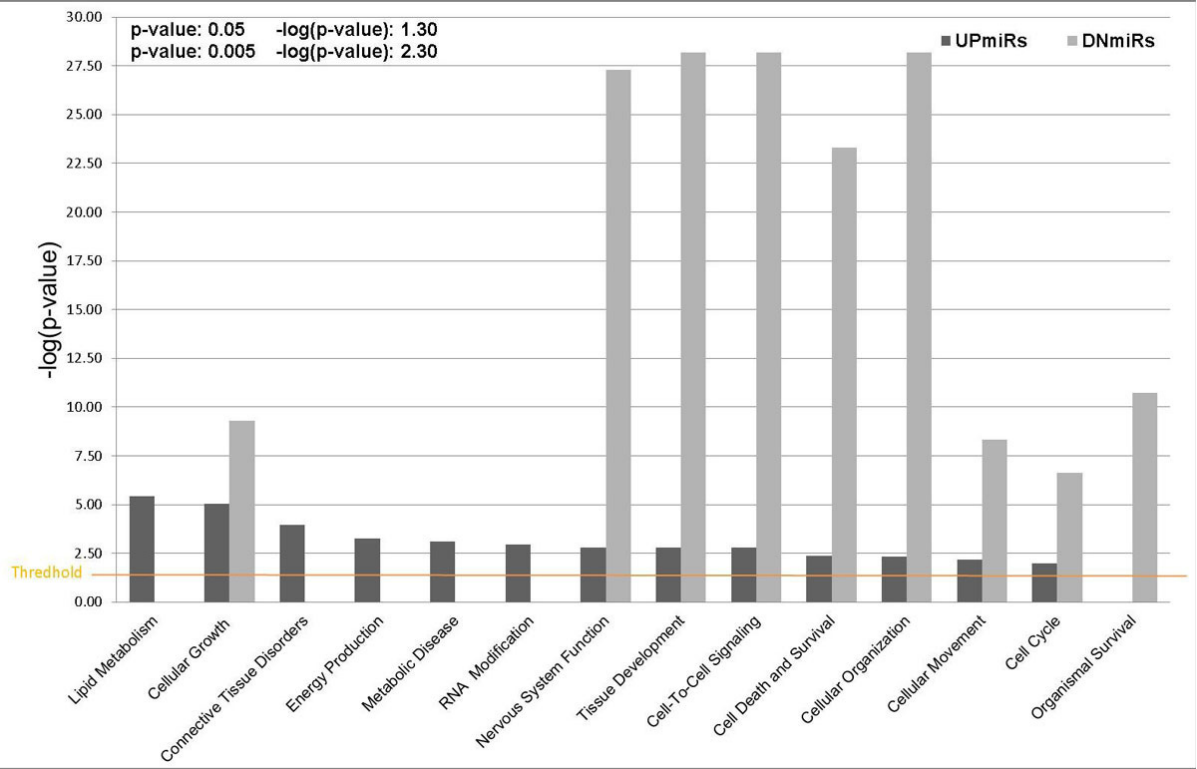
**The target genes of UPmiRs and DNmiRs display distinct functional enrichment in the category of "Diseases and Functions"**. The functions of the UPmiR-targets are involved in the lipid metabolism and RNA processing while the DNmiR-targets are related to cell growth, cell cycle, tissue development, cell-to-cell signalling and cell survival. The threshold significance level is set at *p *< 0.05.

The differentially expressed target genes were analyzed using Ingenuity Pathway Analysis (IPA). Selected molecular and cellular functions significantly enriched in the DEGs under the categories of "Diseases and Biological Functions" and "Canonical Pathways" are respectively listed in Tables 3 and 4, while Tables S5.1 and S5.2 respectively provide detailed gene lists. As shown in Figure 5, different functional enrichment in the category of "Diseases and Functions" was identified for the target genes of UPmiRs and DNmiRs. The functions of the UPmiR-targets are involved in the negative regulation of lipid metabolism, energy production, and RNA processing. They also contribute to connective tissue disorders and metabolic disease. In contrast, the functional roles of the DNmiR-targets are significantly associated with the promotion of cell cycles, tissue development and cell-to-cell signalling.

**Table 3:** Functional analysis of 204 down-regulated target genes of UPmiRs and 295 up-regulated target genes of DNmiRs in IPA "Diseases and Functions".

Diseases and Functions	UPmiRs		DNmiRs	
	
	*p *value	Count	*p *value	Count
Lipid Metabolism	3.85E-06	22		

Cellular Growth and Proliferation	9.18E-06	26	5.17E-10	77

Connective Tissue Disorders	1.12E-04	11		

Energy Production	5.59E-04	5		

Metabolic Disease	8.11E-04	8		

RNA Post-Transcriptional Modification	1.10E-03	3		

Nervous System Development and Function	1.57E-03	25	5.27E-28	61

Tissue Development	1.60E-03	34	6.33E-29	96

Cell-To-Cell Signaling and Interaction	1.64E-03	12	6.33E-29	54

Cell Death and Survival	4.27E-03	12	4.85E-24	105

Cellular Assembly and Organization	4.68E-03	17	6.33E-29	65

Cellular Movement	6.92E-03	6	4.52E-09	58

Cell Cycle	1.06E-02	4	2.28E-07	32

Organismal Survival			1.82E-11	99

Total number of UPmiR-targets: 57				
Total number of DNmiR-targets: 168				

**Table 4:** Function analysis of 204 down-regulated target genes of UPmiRs and 295 up-regulated target genes of DNmiRs in IPA "Canonical Pathways".

Ingenuity Canonical Pathways	UPmiRs		DNmiRs	
	
	*p *value	Count	*p *value	Count
Superpathway of Cholesterol Biosynthesis	8.51E-06	5		

Glycogen Biosynthesis II (from UDP-D-Glucose)	1.10E-03	2		

Pyrimidine Deoxyribonucleotides De Novo Biosynthesis I	2.09E-02	2		

Regulation of the Epithelial-Mesenchymal Transition Pathway	4.57E-02	5	7.76E-02	6

Leukocyte Extravasation Signaling	5.50E-02	5	4.17E-03	9

Integrin Signaling	5.50E-02	5	7.59E-05	12

PDGF Signaling	1.98E-01	2	2.51E-04	7

CXCR4 Signaling	2.12E-01	3	3.02E-03	8

PAK Signaling	2.29E-01	3	2.57E-03	6

p53 Signaling	2.64E-01	3	4.27E-03	6

IGF-1 Signaling	2.64E-01	3	2.19E-05	9

p70S6K Signaling	3.54E-01	3	1.20E-02	6

RhoA Signaling	3.54E-01	3	2.95E-03	7

Axonal Guidance Signaling	4.68E-01	5	5.50E-04	17

Molecular Mechanisms of Cancer	5.28E-01	4	2.14E-03	14

NRF2-mediated Oxidative Stress Response	5.42E-01	2	1.86E-03	9

STAT3 Pathway	5.42E-01	1	1.78E-04	7

TGF-P Signaling	5.98E-01	1	7.08E-05	8

Glioma Signaling	6.35E-01	1	8.32E-04	7

Actin Cytoskeleton Signaling			6.76E-03	9

Hepatic Fibrosis/Hepatic Stellate Cell Activation			2.82E-04	11

Protein Kinase A Signaling			9.33E-04	15

ERK/MAPK Signaling			8.91E-03	8

PTEN Signaling			6.03E-04	8

NF-κB Signaling			5.37E-03	8

Regulation of Actin-based Motility by Rho			2.45E-03	6

Cell Cycle: G2/M DNA Damage Checkpoint Regulation			8.13E-03	4

Total number of UPmiR-targets: 29				
Total number of DNmiR-targets: 76				

**Figure 6:**
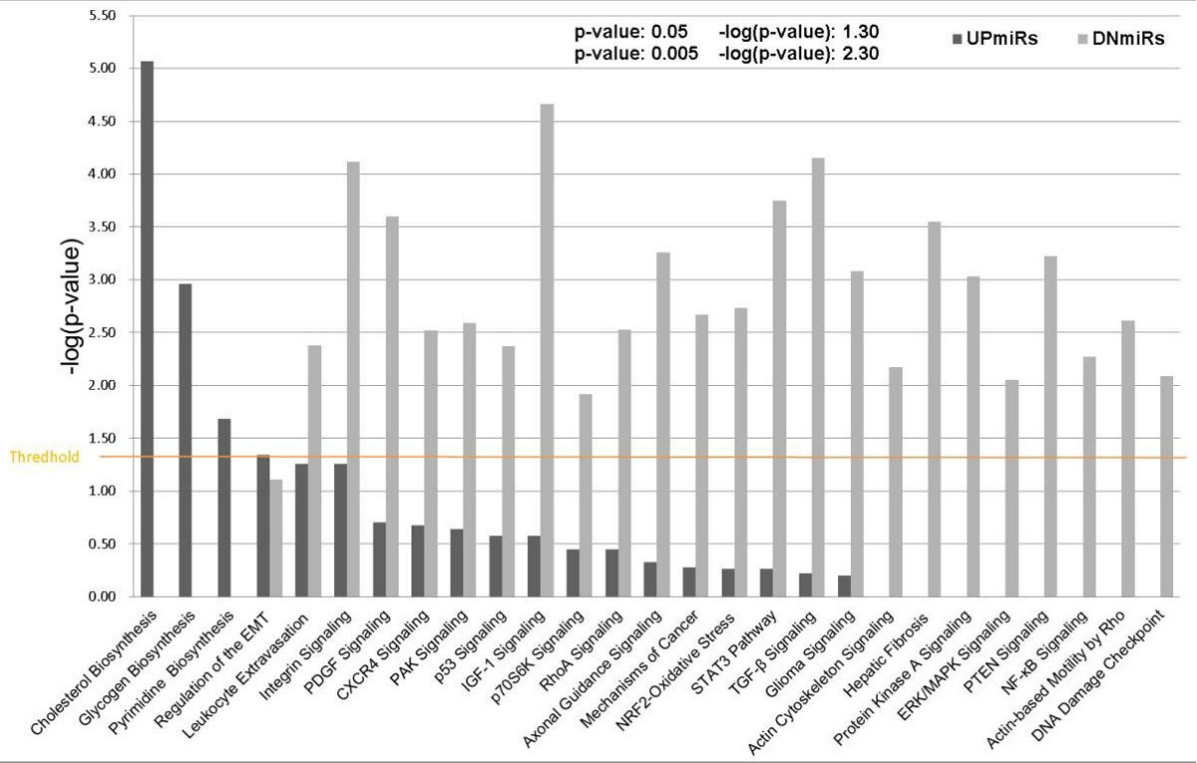
**The target genes of UPmiRs and DNmiRs display distinct functional enrichment in the category of "Canonical Pathway"**. The functions of the UPmiR-targets are involved in biosynthesis of macromolecules while the DNmiR-targets participate in various cell signalling pathways for cell adhesion, proliferation, cell transformation and hepatic fibrosis. The threshold significance level is set at *p *< 0.05.

To further understand their functional involvement, the group of differentially expressed target genes was used for IPA Canonical Pathway analysis. As shown in Figure 6, different functional enrichment was assigned to the target genes of UPmiRs and DNmiRs. The UPmiR-targets are involved in the negative regulation of the biosynthesis of macromolecules, while the DNmiR-targets participates in various cell signalling pathways for cell adhesion, proliferation, cell transformation and hepatic fibrosis. Highly significant enriched pathways such as signalling for actin cytoskeleton/actin-based mobility by Rho, hepatic stellate cell activation, PKA signalling, ERK signalling, PTEN signalling and NF-κB signalling, were enriched only for DNmiR-targets. The IPA analysis results strongly suggest that UPmiRs-targets are involved in loss-of-function activities, while DNmiRs-targets work in a gain-of-function manner. Figure S2.1-S2.22 and S3.1-S3.19 respectively show the pathway presentation of the Causal Network Analysis of "Diseases and Functions" and "Canonical Pathways".

## Discussion

In this case study we analyzed the miRNA-mediated regulatory network in *Mir122a^-/- ^*mouse livers and identified a complex interplay of TF-miRNA and miRNA-targets by way of multiplicity and cooperativity. Using similar control principles, TF and miRNA can jointly fine-tune the target gene expression. The results of this study showed that altered expression of miRNAs in miR-122a-deficient livers had far-reaching effects on liver homeostasis. Previously we demonstrated that fibrotic and tumor signature genes were induced early in *Mir122a *deficient livers [32]. The current study provided additional new evidence to reveal that the interplay between differentially expressed miRNAs and their target genes not only contributes greatly to the properties we previously characterized but also raised new activities to assist the process of cell transformation of *Mir122a^-/- ^*hepatocytes. A general theme of DEmiR-target interaction is that UPmiRs-targets are involved in loss-of-function activities while DNmiRs-targets contribute to gain-of-function activities.

In the absence of the abundant liver-specific miR-122a, the altered expression of DEmiRs did not reinstate the normal physiological functions of miR-122a, but rather expedited the hepatocyte de-differentiation. Several lines of evidence supported our observation. (1) DEmiRs synergistically heighten the adverse effect of miR-122a deficiency. We found a group of 9 DNmiRs that can potentially target 39 miR-122a target genes. These 9 DNmiRs are miR-144-3p, 17-5p, 93-5p, 322-5p, 19a-3p, 31-5p, 145a-5p, 335-5p and 345-5p (Tables S4.1 and S4.2). miR-144-3p and miR-17-5p can respectively recognize 19 and 17 miR-122a targets. Upregulation of the miR-122a-co-targets contribute to altered cell adhesion, cytoskeleton organization, protein phosphorylation, ion transport, glucose catabolic process and liver fibrosis. (2) Induced expression of Chr12qF1 miRNAs was detected in mutant hepatocytes. The imprinted Dlk1-Dio3 gene cluster is located on mouse chromosome 12 (12qF1) and human chromosome14 (14q32.2). Concurrent overexpression of Chr12qF1 miRNAs is regarded as the miRNA signature of the stem cell property [35,36] and overexpression of 14q32.2 miRNAs has been detected in cancer stem cells and in aggressive types of human HCC [37] as well as in other cancer types [38]. (3) Upregulation of several UPmiRs has been reported in HCC despite the fact the 2-month old *Mir122a^-/- ^*livers are free of tumors. Elevated expression of miR-182 [39], miR-429 [40], miR-199a, 199a*, 200a, and 200b was positively and significantly correlated to the progression of liver fibrosis [41]. These results reinforce our previous findings that dysregulation of liver functions begins in young *Mir122a^-/- ^*mice [32].

In *Mir122a^-/- ^*livers, overexpression of several classic imprinted genes *H19, Igf2, Meg3, Mirg *and *Rian *signals for altered epigenetic regulation. How miR-122 influences epigenetic regulation of DEGs is not clear. It has been reported that many miRNA targets are involved in gene regulation at the epigenetic level [42]. From the curated miRNA-target interactions, several enzymes affecting methylation of DNA or histones were found to be the target genes of UPmiRs. Tet methylcytosine dioxygenase 2 (Tet2), lysine (K)-specific demethylase 7A (Kdm7a) and jumonji domain containing 1C (Jmjd1c, a H3K9 demethylase) are respectively targeted by 8, 9 and 5 UPmiRs (Table S3.2). Whether reduced expression of these enzymes is linked to epigenetic regulation of DEGs in *Mir122a^-/- ^*mice requires further experimental demonstration. It is worth mentioning that CTCF, an insulator-and chromatin loop-associated protein as well as an epigenetic regulator, is a direct target of miR-122 [33].

TF-DEmiR interactions revealed that Ctcf, a hub-TF, potentially can regulate 42 DEmiRs including 32 UPmiRs and 10 DNmiRs. A great proportion of the Ctcf-UPmiRs (94%) is located in the Chr12qF1 locus. Recently, CTCF was found to be involved in the regulation of *miR-125b1, miR-375*, and the *miR-290 *cluster in breast cancer cells and stem cells [43]. DNA methylation seems to play a role in the CTCF regulation of miRNAs since CTCF binding sites tend to be mapped to the hypomethylated regions [44]. Our initial methylome data also showed that the Ctcf binding sites in the Chr12qF1 locus are less methylated in *Mir122a^-/- ^*than in the WT liver (data not shown).

## Conclusions

We applied our computational framework to the expression profiles of the miRNA/mRNA from *Mir122a^-/- ^*mutant mice and wild-type mice and found a miRNA-mediated network involving 40 curated TFs regulating the aberrant expression of 65 miRNAs and 723 curated miRNA target genes, of which 56% was found in the differentially expressed genes of *Mir122a^-/- ^*mice. This regulatory network disclosed previously known as well as many previously unidentified miRNA-mediated regulations in mutant mice. Remarkably, we demonstrate that loss of imprinting at 12qF1 is associated with miRNA overexpression in human HCC and stem cells, suggesting initiation of precancerous changes in young mice deficient in miR-122. In addition, a group of 9 miRNAs was found to share miR-122 target genes, indicating synergy between miRNAs and target genes by way of multiplicity and cooperativity. These findings have a potentially significant impact on our understanding of miRNA-mediated regulatory networks. By collectively utilizing the experimentally verified data, this computational framework is highly reliable, effective and is suitable for exploring significant TF-miRNA-miRNA target interactions in any complex biological system.

## Methods

### Computational framework for reconstructing the miRNA-mediated regulatory network

Figure 1 presents an overview of the proposed computational framework to reconstruct the miRNA regulatory network. We obtained mouse TF-miRNA regulation information from TransmiR and ChIPBase and those regulations related to differentially expressed miRNAs in the livers of *Mir122a^-/- ^*and wild type mice were selected. The differentially expressed miRNAs were determined by expression profiles using the OpenArray system and small RNA-Seq. The differentially expressed genes were identified from the expression profiles using gene chips. Moreover, we integrated experimental miRNA-target gene interactions from miRTarBase, TarBase and starBase. For each miRNA-target interaction, miRNA and its target gene with inverse expression level were selected for further reconstruction of the miRNA-mediated regulatory network.

### Curated TF-miRNA interactions and construction of the TF-miRNA regulatory network

To reconstruct the high confidence TF-miRNA regulatory network in *Mir122a^-/- ^*mice, we integrated two experimentally verified resources, TransmiR (version 1.2) [14] and ChIPBase (release 1.1) [16], to support the regulations in TF-miRNA. TransmiR is the TF-miRNA interaction database, and contains 735 manually curated TF-miRNA regulatory interactions. ChIPBase provides the transcriptional regulation not only for the protein-coding genes, but also for non-coding RNA (including miRNA) from ChIP-Seq data. The miRNA promoter region used in this study is defined as a 5 kb upstream region and 1 kb downstream region. Transcription factor binding sites (TFBSs) from ChIP-Seq data on each miRNA promoter provide empirical evidence of TF-miRNA interactions. Table S1 summarizes the 8,697 curated TF-miRNA interactions between 73 TFs and 964 miRNAs in mouse livers. The TF-miRNA regulatory network was constructed based on the interactions between differentially expressed miRNAs and their experimentally verified TFs. We used Cytoscape (version 3.1.1) [45-48] to visualize the TF-miRNA regulatory network.

### Curated miRNA-target interactions and construction of the miRNA-target regulatory network

To construct the miRNA-mediated regulatory network in *Mir122a^-/- ^*mice, we collected datasets of experimentally validated miRNA-target interactions (MTIs) from miRTarBase [49], TarBase [50] and starBase [51]. The miRTarBase release 4.5 dataset http://mirtarbase.mbc.nctu.edu.tw contains more than fifty thousand miRNA-target interactions, obtained by manually surveying the relevant literature using textual data mining tools to systematically filter research articles related to functional studies of miRNAs. Generally, the collected MTIs have been validated experimentally by 3'UTR-reporter assay, western blotting, microarray analysis and by next-generation sequencing experiments. TarBase (version 6) is the other data source for obtaining the experimentally validated MTIs. This work not only integrated the MTIs from miRTarBase and TarBase, we also collected MTIs from Argonaute CLIP-Seq data. starBase is a database used for deciphering miRNA-target interactions accumulated from 108 CLIP-Seq data from 37 studies. For this study, we retrieved MTIs only from data derived from mouse tissue. Table S1 summarizes the relationships among the 95,364 curated MTIs between 329 miRNAs and 7,001 target genes.

To construct the miRNA-target regulatory network, we gathered the DEmiRs and DEGs respectively from the miRNA expression profiles (OpenArray and small RNA-Seq) and microarray datasets. We identified the transcriptional regulation and the post-transcription regulation of DEmiRs from experimentally verified resources. An inverse relationship in expression was often found between miRNAs and their target genes [52-55], thus we focused on those inversely correlated pairs of DEmiRs and the curated targets. For each up-regulated miRNA, the curated target genes are down-regulated and vice versa. These interactions are imported to Cytoscape for visualizing the miRNA-target regulatory network.

### Functional and pathway analysis using Ingenuity Pathway Analysis (IPA)

To discuss the TF-miRNA regulatory network, we analyzed the target genes of differentially expressed miRNAs using Ingenuity Pathways Analysis 2014 (IPA Summer Release 2014, Ingenuity Systems, http://www.ingenuity.com). IPA analysis identified biological functions and canonical pathways related to the targets of up-and down-regulated miRNAs and enabled comparison across multiple analyses with varying conditions. A threshold was set at *p *value 0.05 which was calculated by Fisher's exact test. Causal Network Analysis provides the causal connections between diseases, genes and networks of upstream regulators [56].

### RNA isolation, gene expression, and high-density oligonucleotide microarray analysis

Total RNA was isolated from the liver samples of wild-type mice and *Mir122a^-/- ^*mice using the Trizol reagent (Invitrogen) according to the manufacturer's protocol. The microarray hybridizations were performed using total RNA prepared from the liver samples of three wild-type mice and four *Mir122a^-/- ^*mice at an age of 2-months. GeneChip Mouse Genome 430 2.0 Affymetrix oligonucleotide Gene Chips (Affymetrix) were analyzed at the Microarray & Gene Expression Analysis Core Facility (VGH-YM Genome Center, National Yang-Ming University) according to the Affymetrix protocols. All the data files are presented in compliance with MIAMI guidelines and can be accessed online at the Gene Expression Omnibus (series accession numbers GSE27713 [32]). A total of 2315 differentially expressed genes was identified in the *Mir122a^-/- ^*mice, 1,448 genes up-regulated (a fold change of ≥ 1.5) and 867 genes down-regulated (a fold change of ≤ 0.66).

### miRNA Profiling on high-throughput OpenArray™ system

MiRNA expression profiling of the liver sample from one wild-type mouse and one *Mir122a^-/- ^*mouse (both 2-months old) was performed using TaqMan Rodent MicroRNA Array v2.0 (Applied Biosystems) according to the manufacturer's instructions. This array panel enables the simultaneous quantification of the expression of 750 rodent (mouse and rat) well-characterized mature miRNAs. All the mature miRNA names are mapped to the name recorded in miRBase V20. Expression data were processed using OpenArray™ Real-Time qPCR Analysis Software. The data were further analyzed in HTqPCR package from Bioconductor (v2.1.2) in R 2.23. Data were quantile normalized and duplicates averaged using U6 rRNA as an endogenous control. Undetermined miRNAs or those with a Ct value below 15 or greater than 35 across all samples were removed from subsequent analysis. We set the cut-off for the up-regulated miRNAs with ≥ 1.5 fold change, or ≤ 0.66 for down-regulated miRNAs.

### Small RNA-Seq data analysis

The same liver samples that were analysed by OpenArray™ system were further subjected to small RNA sequencing. The sequencing libraries for miRNA-Seq were prepared using TruSeq Small RNA Sample Preparation Kit (Illumina) according to the manufacturer's instruction. One microgram of total RNA with an RNA integrity number (RIN) greater than 8.0 was ligated sequentially to 3' and 5' adaptors. Subsequently, the ligation product was reverse transcribed followed by PCR for 11 cycles to enrich miRNAs that have adapter sequences on both ends. The amplified cDNA construct was size fractionated (145-160 bp) and purified by 6% Novex TBE PAGE gel (Invitrogen). The miRNA library was quantified by qPCR and the size distribution was validated on a 2100 Bioanalyzer (Agilent) by a High Sensitivity DNA chip. The miRNA library was sequenced on a HiSeq 2000 (Illumina) by single end sequencing with a 50 bp read length.

Raw sequencing reads were quality pre-processed using the FASTX-Toolkit [57] version 0.0.13 as follows. First, the Illumina 3' adapter sequences 'TGGAATTCTCGGGTGCCAAGG' were removed. The reads were then trimmed according to their quality values based on the Phred quality score. We set a Phred quality score of 20 as the cut-off value. We obtained the small RNA reads if the reads were longer than 18 nucleotides and shorter than 30 nucleotides. We then used the ncPRO-seq [58] package (version 1.5.1), a bowtie-based [59] read alignment tool for the annotation reads, to confirm the read distribution in the reference genome. Only reads that mapped a maximum of two mismatches and 20 locations in the genome were used (bowtie parameter: -v2 -a -m20 --best --strata --nomaqround -f -y). Five main databases were employed to annotate the small RNA distribution: the UCSC reference genome (mm10), miRBase v20 [60,61], UCSC refGene 06-Apr-2014, RFam v11.0 [62], and UCSC repeatMasks (mm10). To quantify the miRNA profiles, we used miRDeep2 [63,64] package (version 2.0.0.5), another bowtie-based alignment tool. Figure S1 shows that more than 65% of small RNA reads were miRNAs, indicating that our small RNA reads were highly enriched for miRNAs. The miRNAs were identified as significantly differentially expressed as compared to those in normal livers if the fold change was greater than or equal to 1.5 (up-regulated miRNAs) or the fold change was less than or equal to 0.66 (down-regulated miRNAs).

## Competing interests

The authors declare that they have no competing interests.

## Authors' contributions

SDH and APT conceived and designed the experiments. SDH, HYH, CHC and YMS analyzed the data and performed the experiments. SDH, HYH, CHC, MTH and APT wrote the paper.

## Supplementary Material

Additional file 1 **Table S1 **- Summary of the relationships among curated miRNAs, TFs and target genes. Click here for file
