# Supplementary material for: Integrated analyses to reconstruct microRNA-mediated regulatory networks in mouse liver using high-throughput profiling
Source: BMC Genomics. 2015 Jan 21;16(Suppl 2):S12. doi: 10.1186/1471-2164-16-S2-S12 (PMC4331712; doi:10.1186/1471-2164-16-S2-S12)
Supplement: Additional file 1 — Table S1 - Summary of the relationships among curated miRNAs, TFs and target genes. [file 1471-2164-16-S2-S12-S1.pdf]

Table S1 – Summary of the relationships among curated miRNAs, TFs and target genes.

Relationship	TF-miRNA	miRNA-target
**Number of pairs**	8,697	95,364
**Number of TFs**	73	-
**Number of miRNAs**	964	329
**Number of genes**	-	7,001
**Data source**	TransmiR and ChIPBase	TarBase, miRTarBase and starBase

Table S2.1 – Detailed curated TF-miRNA: Statistics of TF-regulated miRNAs.

miRNA	TFs	Number of TFs
mmu-miR-339-3p	E2f1;Wdr5;Foxa2;Pparg;Klf4;E2f4;Sirt1;Sin3a;Erg;Sin3b;Cebpa;Ep300;Gata4;Spi1;Cebpb;Srf;Mycbp;Mycn;Esr1;Rbbp5	20
mmu-miR-17-5p	E2f4;Zfx;Spi1;Mycbp;Suz12;Tfcp2l1;Klf4;Gata1;Mycn;Cebpb;Erg;Ctcf;Foxa2;Sirt1;Cebpa;E2f1;Wdr5;Ep300	18
mmu-miR-19a-3p	Spi1;Foxa2;Suz12;Klf4;Erg;E2f4;Mycbp;Wdr5;Mycn;E2f1;Ctcf;Sirt1;Gata1;Ep300;Zfx;Cebpb;Tfcp2l1;Cebpa	18
mmu-miR-326-3p	Tcf3;Lyl1;Meis1;Spi1;Gata4;Suz12;Gata2;Cebpb;Lmo2;Cebpa;Mycn;Gata1;Fli1;Erg;E2f4;Sirt1;Rbbp5;Mtf2	18
mmu-miR-93-5p	Gata1;E2f4;Esr1;Nkx2-5;Erg;Srf;Cebpb;Gata4;Fli1;Sin3b;Ep300;Foxa2;Wdr5;Spi1;Sirt1;Cebpa;Pparg;Tcf3	18
mmu-miR-484	Cebpb;Spi1;E2f1;Ctcf;Ep300;Sirt1;Mycn;Rbbp5;Erg;E2f4;Srf;Cebpa;Wdr5	13
mmu-miR-182-5p	Zfx;Rbbp5;Tfcp2l1;Spi1;Cebpb;Klf4;Pparg;Wdr5;E2f1;Esrrb;Gata4;Ctcf	12
mmu-miR-200c-3p	Esr1;Gata4;Zfx;Cebpb;Tcf3;Gata1;Klf4;E2f1;Esrrb;Spi1;Cebpa	11
mmu-miR-486b-5p	Esrrb;Zfx;Ctcf;Suz12;Tfcp2l1;Rbbp5;Mtf2;Spi1;Mycn;Gata4	10
mmu-miR-455-5p	Jarid2;Ezh2;Ctcf;Sin3b;Rbbp5;Mtf2;Suz12;Wdr5	8
mmu-miR-455-3p	Ezh2;Ctcf;Jarid2;Rbbp5;Mtf2;Sin3b;Suz12;Wdr5	8
mmu-miR-199a-5p	Spi1;E2f4;Ep300;Cebpb;Gata2;Ctcf;Erg	7
mmu-miR-31-3p	Cebpa;Ctcf;Hnf4a;Cdx2;Foxa2	5
mmu-miR-31-5p	Foxa2;Cdx2;Hnf4a;Ctcf;Cebpa	5
mmu-miR-429-3p	Myc;Tfcp2l1;E2f1;Esr1	4
mmu-miR-802-5p	Ctcf;Foxa2;Gata4;Cebpa	4
mmu-miR-322-5p	Suz12;Spi1;Hif1a	3
mmu-miR-200a-3p	E2f1;Esr1;Tfcp2l1	3
mmu-miR-200b-3p	Esr1;E2f1;Tfcp2l1	3
mmu-miR-145a-5p	Ep300;E2f1;Ctcf	3
mmu-miR-451a	Gata1;Ep300;Esr1	3
mmu-miR-144-3p	Esr1;Gata1;Ep300	3
mmu-miR-431-5p	Spi1	1
mmu-miR-485-3p	Ctcf	1
mmu-miR-411-5p	Ctcf	1
mmu-miR-1193-3p	Ctcf	1
mmu-miR-376c-3p	Ctcf	1
mmu-miR-379-5p	Ctcf	1
mmu-miR-411-3p	Ctcf	1
mmu-miR-410-3p	Ctcf	1
mmu-miR-376b-3p	Ctcf	1
mmu-miR-381-3p	Ctcf	1
mmu-miR-540-3p	Spi1	1
mmu-miR-382-5p	Ctcf	1
mmu-miR-136-3p	Spi1	1
mmu-miR-300-3p	Ctcf	1
mmu-miR-376a-3p	Ctcf	1
mmu-miR-369-3p	Ctcf	1
mmu-miR-335-5p	Ep300	1
mmu-miR-127-5p	Spi1	1
mmu-miR-409-3p	Ctcf	1
mmu-miR-543-3p	Ctcf	1
mmu-miR-299a-5p	Ctcf	1
mmu-miR-495-3p	Ctcf	1
mmu-miR-337-3p	Spi1	1

mmu-miR-379-3p	Ctcf	1
mmu-miR-485-5p	Ctcf	1
mmu-miR-154-3p	Ctcf	1
mmu-miR-127-3p	Spi1	1
mmu-miR-434-3p	Spi1	1
mmu-miR-409-5p	Ctcf	1
mmu-miR-494-3p	Ctcf	1
mmu-miR-496a-3p	Ctcf	1
mmu-miR-369-5p	Ctcf	1
mmu-miR-376b-5p	Ctcf	1
mmu-miR-541-5p	Ctcf	1
mmu-miR-382-3p	Ctcf	1
mmu-miR-329-3p	Ctcf	1
mmu-miR-345-5p	Spi1	1
mmu-miR-337-5p	Spi1	1
mmu-miR-673-3p	Spi1	1
mmu-miR-154-5p	Ctcf	1
mmu-miR-434-5p	Spi1	1
mmu-miR-134-5p	Ctcf	1
mmu-miR-136-5p	Spi1	1
Total: 65 miRNAs*	40 TFs*	240 TF-miRNA interactions*

* indicates the unique gene count

Table S2.2 – Detailed curated TF-miRNA: Statistics of TF-regulated miRNAs.

TF	miRNAs regulated by TF	Number of miRNAs
Ctcf	miR-486b-5p;miR-17-5p;miR-485-3p;miR-182-5p;miR-411-5p;miR-1193-3p;miR-376c-3p;miR-31-3p;miR-379-5p;miR-411-3p;miR-410-3p;miR-376b-3p;miR-455-5p;miR-381-3p;miR-455-3p;miR-382-5p;miR-300-3p;miR-376a-3p;miR-369-3p;miR-19a-3p;miR-199a-5p;miR-409-3p;miR-543-3p;miR-145a-5p;miR-299a-5p;miR-495-3p;miR-379-3p;miR-485-5p;miR-154-3p;miR-409-5p;miR-494-3p;miR-496a-3p;miR-369-5p;miR-376b-5p;miR-484;miR-541-5p;miR-382-3p;miR-329-3p;miR-802-5p;miR-31-5p;miR-154-5p;miR-134-5p	42
Spi1	miR-486b-5p;miR-17-5p;miR-431-5p;miR-182-5p;miR-322-5p;miR-339-3p;miR-540-3p;miR-136-3p;miR-19a-3p;miR-127-5p;miR-199a-5p;miR-337-3p;miR-326-3p;miR-127-3p;miR-434-3p;miR-484;miR-345-5p;miR-200c-3p;miR-337-5p;miR-673-3p;miR-434-5p;miR-136-5p;miR-93-5p	23
E2f1	miR-17-5p;miR-182-5p;miR-339-3p;miR-200a-3p;miR-200b-3p;miR-19a-3p;miR-145a-5p;miR-429-3p;miR-484;miR-200c-3p	10
Cebpa	miR-17-5p;miR-31-3p;miR-339-3p;miR-19a-3p;miR-326-3p;miR-484;miR-802-5p;miR-200c-3p;miR-31-5p;miR-93-5p	10
Ep300	miR-17-5p;miR-339-3p;miR-335-5p;miR-19a-3p;miR-199a-5p;miR-145a-5p;miR-451a;miR-484;miR-144-3p;miR-93-5p	10
Cebpb	miR-17-5p;miR-182-5p;miR-339-3p;miR-19a-3p;miR-199a-5p;miR-326-3p;miR-484;miR-200c-3p;miR-93-5p	9
Wdr5	miR-17-5p;miR-182-5p;miR-339-3p;miR-455-5p;miR-455-3p;miR-19a-3p;miR-484;miR-93-5p	8
Esr1	miR-339-3p;miR-200a-3p;miR-200b-3p;miR-451a;miR-429-3p;miR-200c-3p;miR-144-3p;miR-93-5p	8
Tfcp2l1	miR-486b-5p;miR-17-5p;miR-182-5p;miR-200a-3p;miR-200b-3p;miR-19a-3p;miR-429-3p	7
Rbbp5	miR-486b-5p;miR-182-5p;miR-339-3p;miR-455-5p;miR-455-3p;miR-326-3p;miR-484	7
Foxa2	miR-17-5p;miR-31-3p;miR-339-3p;miR-19a-3p;miR-802-5p;miR-31-5p;miR-93-5p	7

E2f4	miR-17-5p;miR-339-3p;miR-19a-3p;miR-199a-5p;miR-326-3p;miR-484;miR-93-5p	7
Suz12	miR-486b-5p;miR-17-5p;miR-322-5p;miR-455-5p;miR-455-3p;miR-19a-3p;miR-326-3p	7
Gata4	miR-486b-5p;miR-182-5p;miR-339-3p;miR-326-3p;miR-802-5p;miR-200c-3p;miR-93-5p	7
Gata1	miR-17-5p;miR-19a-3p;miR-326-3p;miR-451a;miR-200c-3p;miR-144-3p;miR-93-5p	7
Erg	miR-17-5p;miR-339-3p;miR-19a-3p;miR-199a-5p;miR-326-3p;miR-484;miR-93-5p	7
Mycn	miR-486b-5p;miR-17-5p;miR-339-3p;miR-19a-3p;miR-326-3p;miR-484	6
Sirt1	miR-17-5p;miR-339-3p;miR-19a-3p;miR-326-3p;miR-484;miR-93-5p	6
Klf4	miR-17-5p;miR-182-5p;miR-339-3p;miR-19a-3p;miR-200c-3p	5
Zfx	miR-486b-5p;miR-17-5p;miR-182-5p;miR-19a-3p;miR-200c-3p	5
Sin3b	miR-339-3p;miR-455-5p;miR-455-3p;miR-93-5p	4
Mtf2	miR-486b-5p;miR-455-5p;miR-455-3p;miR-326-3p	4
Tcf3	miR-326-3p;miR-200c-3p;miR-93-5p	3
Pparg	miR-182-5p;miR-339-3p;miR-93-5p	3
Srf	miR-339-3p;miR-484;miR-93-5p	3
Esrrb	miR-486b-5p;miR-182-5p;miR-200c-3p	3
Mycbp	miR-17-5p;miR-339-3p;miR-19a-3p	3
Cdx2	miR-31-3p;miR-31-5p	2
Hnf4a	miR-31-3p;miR-31-5p	2
Gata2	miR-199a-5p;miR-326-3p	2
Jarid2	miR-455-5p;miR-455-3p	2
Fli1	miR-326-3p;miR-93-5p	2
Ezh2	miR-455-5p;miR-455-3p	2
Lyl1	miR-326-3p	1
Sin3a	miR-339-3p	1
Hif1a	miR-322-5p	1
Meis1	miR-326-3p	1
Myc	miR-429-3p	1
Nkx2-5	miR-93-5p	1
Lmo2	miR-326-3p	1
Total: 40 TFs*	65 miRNAs*	240 TF-miRNA interactions*

* indicates the unique gene count

Table S3.1 – Single miRNA can target multiple target genes: list of DEGs targeted by miRNA.

miRNA	Target genes	Number of target genes
mmu-miR-17-5p	Col3a1;Zmat3;Itgb8;App;Amot;Adcy7;Gpm6b;Thy1;Rragd;Pafah1b2;Maf1;Igfbp7;Dnajb9;Tes;Cxcl12;Pvr;Aebp2;Rab30;Dcn;Sqstm1;Adam10;Fbxo25;Rogdi;Clic1;Jup;Slc41a1;Grb10;Tox;Rpia;Map3k1;Ildr2;Ank2;Csnk1g1;Itm2c;Tob2;Trp53inp1;Ankrd13c;Pacs2;Arhgap1;Ttc37;Fam84b;Armc8;Rtn3;Coq10b;Ccng1;Zfp704;Rbl1;Pfn2;Tgfbr1;Id4;Atp2b2;Nfe2l2;Laptm5;Top2a;Mtpn;Nptx1;Nup35;Mknk2;Snx18;Tnfaip3;Col4a2;Sema3c;Wars;Ccnd2;Brms1l;Tgfbr2;Btg2;Tnfrsf21;Fam63b;Txnip;Aak1;Pim3;Dusp6;Pip4k2a;Plscr3;Rell1;Cav1;Dpysl2;Rasal1;Pdgfra;Rab5c;Ubfd1;Socs3;Cxcr4;Bmp2k;Rabgap1l;Entpd7;Cpe;Ptgfrn;Stx6;Smoc2;Klf6;Smap1;Mapre1;4933426M11Rik;Sparcl1;Bcl6;Jak1;Rnf38;Spp1;Dync1li2;Zbtb4;Pfkp;Fam49b;Plod2;Rcan3;Flnb;Tnrc6c;Nck2;Slc1a4;Ano6;Plk3;Stx3;Timp3;Timp2	115
mmu-miR-93-5p	Smap1;Tob2;Arhgap1;Txnip;Dpysl2;Ccng1;Cpe;Dync1li2;Maf	83

	1;Jup;Plscr3;Spp1;Sparcl1;Dcn;Trp53inp1;Cxcr4;Mknk2;Socs3;Clic1;Rnf38;Tes;Stx6;Stx3;Armc8;Ccnd2;Rbl1;Id4;Rell1;Sqstm1;4933426M11Rik;Aak1;Adcy7;Pdgfra;Dusp6;Pacs2;Ank2;Timp3;Ankrd13c;Nfe2l2;Atp2b2;App;Tnfrsf21;Pfkp;Aebp2;Nup35;Ptgfrn;Nck2;Jak1;Klf6;Col4a1;Coq10b;Tgfbr2;Kbtbd11;Bcl6;Ano6;Pafah1b2;Rabgap1l;Tox;Slc41a1;Atp6v0e;Zbtb4;Rcan3;Ttc37;Rab5c;Csnk1g1;Top2a;BC029722;Plk3;Fam63b;Col3a1;Enc1;Ubfd1;Tnrc6c;Fam84b;Btg2;Snx18;Pvr;Fbxo25;Plod2;Dnajb9;Mapre1;Cav1;Pfn2	
mmu-miR-322-5p	Erlin2;Tmtc2;Pafah1b2;Ubfd1;Gskip;Ly6e;Trim35;Gltp;Bmpr1a;Ywhag;Ccng1;Calm1;Stxbp1;Nol4l;Sparc;Trp53inp1;Sgk1;Dync1li2;Btla;Sos2;C1ql3;Cacul1;Cx3cl1;Bmp2k;Lims1;Usp2;Tob2;Nf2;Slc35g1;Pard6b;Aldoa;Tuba1a;Otud7b;Igf2;Ank2;Ccnd2;Sipa1l2;Rtn3;Pfn2;Kcnn4;Slc9a6;Bloc1s6;Hjurp;Sept11;Rtn4;Alg2;A730008H23Rik;Mapk3;Mpp1;Armcx2;Cpd;Armc8;Kif5b;Col3a1;Gpr124;Atp2b2;Cbx5;Cbx6;Add1;Nfe2l1;Atp6v1b2;Npcd;Maf1;G3bp2;Bcl2;Man2a1;Wars;Zfp106;Btg2;Mapre1;Fosl2;Slc4a4;Gpsm3;Mapkapk3;Avl9;Arl2;Pmepa1;Tnfaip3;Txnip;Mtpn	80
mmu-miR-144-3p	Ddit4;Tox;Gja1;Aqr;Ank2;Fam134b;Mtpn;Nptx1;Pfkp;Tgfbr1;Itm2a;Adam10;Calm1;Calm2;Pde4d;Bmpr1a;Msn;Rtn4;Rbl1;Dync1li2;Tgif1;Dnajc13;Slc4a4;Kif5b;Nfe2l2;Map3k1;Abi2;Gpm6b;Wars;Scd2;Herpud1;Cbl;Nedd4l;Atp1b1;Klf2;Klf6;Brca1;Tom1l2;C1ql3;Plekha2;Eln;Atp11a;Ndrg3;Myl12a;Slc1a2;Alcam;Wdr45b;Cd200;G3bp2;Atp2b2;Trp53inp1;Nacc2;Nedd9;Ptgfrn;Aebp2;Ablim1;Id4;Sgcb;Ngfrap1;Atp8a1;Amot;App;Cxcl12;Ikzf1;Appbp2;Rnf38;Vamp3;Timp3;Fosl2;Trim35;Slc38a2;Agfg1	72
mmu-miR-19a-3p	Cs;Aebp2;Ldhb;Gpm6b;Sgcb;Klf6;Armc8;Armcx2;G3bp2;Igfbp3;Plekha2;Vamp3;Tgfbr2;Npcd;Rell1;Nedd4l;Atp2b2;Dnajb9;Calm2;Calm1;Ccrn4l;Arl8b;Slc9a6;Timp2;Tnrc6c;Fosl2;Trp53inp1;Tnfaip3;Fam20b;Grb10;Tob2;Atp8a1;Enc1;Arhgap1;Map3k1;Cbx6;Socs3;Adcy7;Bcl6;Stx6;Pmepa1;Clcn5;Atp6v1b2;Snx17;Atp11a;Clic4;Cacul1;Tgif1;Gskip;Slc6a8;Wdr45b;Ablim1;Rhob;Ccnd2;Lbh;Rnf38;Atp10a;Nup35;Prkcb;Gja1;Tnip1;Otud7b	62
mmu-miR-381-3p	Aqp4;Rab14;2810403A07Rik;Phtf2;Hectd2;Kdm7a;Fgfr1op2;Klhl2;Srsf1;Trpm7;Id2;Hmgcr;Arntl;Ythdf3;Pnrc2;Crybg3;Syncrip;Arap2;Fnip1;Gid4;Prpf4b;Zkscan1;Rnf138;Cldn12;Dock4;Rrm2;Sigmar1;Tet2;Fam126b;Ppip5k2;Rbm26;Rbm25;Fam208a;Rictor;Elovl5;Nr3c1;Rnpc3;4932438A13Rik;Ube2w;Cnbp;Nfia;Nfib;Clock;Tmed5;2310061I04Rik	45
mmu-miR-495-3p	Aqp4;Ppp6r3;Tmem56;4932438A13Rik;Dmd;Btg1;Ddx3y;Pnisr;Ak4;AI987944;March7;Fubp1;Rrm2;Rictor;Fam208a;Klhl24;Nfib;Srsf11;Stat1;Arntl;Tmem135;Id2;Mtfr1;Vma21;Brwd3;Cpsf6;Tbc1d30;Ube2w;Pten;Fam126b;Prpf4b;Fnip1;Onecut1;Mapk8;Acadsb;Nr3c1;Mbtd1;Ythdf3;Cited2;Jmjd1c	40
mmu-miR-429-3p	Sigmar1;Dmd;Plxna2;4932438A13Rik;Btg1;Nfib;Fgd4;Ddx3y;Cnot6l;Hectd2;Tk1;Gbp6;Ythdf3;Brwd3;C1galt1;Arntl;Whsc1l1;Ddx5;Cited2;Rab14;Rbm26;Zfp871;Mtfr1;Fam107b;Ak4;Sqle;Scd1;Zfp770;Pcmtd1;Desi1;Zeb1;Msmo1;Cfl2;Spred1;Gnai3;Kdm7a;Fermt2;Arhgap5;Zfr	39
mmu-miR-31-5p	Ndrg3;4931406P16Rik;Wtip;Hjurp;Klf2;Mvb12b;Grb10;Tmed9;Cotl1;Susd2;Wars;Peg3;G3bp2;Atp8a1;Ifi30;Atp2b2;Pip4k2a;Txnip;Sbk1;B4galt6;Trp53inp1;Ywhab;Tpm4;Rab5c;Klf6;Kctd5;Lbh;Slc43a2;A730008H23Rik;Magt1;Ank2;Ablim1;Tes;Map3k1;Wipf1;Kif5b	36
mmu-miR-543-3p	Clpx;Crybg3;Trp53inp2;Hnrnpa2b1;Maml1;Tet2;Hmgcr;Spred1;Eea1;Cfl2;Ube2w;Ccdc58;Fubp1;Brwd3;Ythdf3;Elovl5;Cnot6l;Zfp709;Ppip5k2;Ppp1r3b;Pdia6;Kdm7a;Pten;Id2;Nr6a1;Doc	36

	k4;Syncrip;Pabpc1;Pnrc2;Nr3c1;Rab14;Fnip1;Zfp800;Trpm7;Evi5;Pcmtd1	
mmu-miR-451a	Sparc;Dusp6;Pcdhgc5;Ctnnal1;Thbd;Pcdhga3;Pcdhga1;Pcdhga6;Wwtr1;Pcdhgc3;Pcdhgc4;Oat;Avl9;Pcdhga10;Pcdhga12;Calm1;Pcdhga11;Pcdhgb8;Pcdhgb7;Pcdhgb6;Pcdhgb5;Pcdhgb4;Pcdhgb2;Pcdhgb1;Man2a1;Plod2;Pcdhga2;Pcdhga4;Pcdhga7;Pcdhga5;Pcdhga8	31
mmu-miR-410-3p	Cited2;Inpp1;Fermt2;Ythdf3;Cnbp;Phtf2;Plxna2;Syncrip;Fubp1;Aqp4;Slc30a1;Dmd;Ahctf1;Sfpq;Rreb1;Gnai3;Rictor;Kdm7a;Ube2w;Spred1;Id2;Pten;Hectd2;Hlf;Tet2;Whsc1l1;Vegfa;Maml1;Nfib	29
mmu-miR-145a-5p	Ccnd2;Sos2;Fundc1;Tgfbr2;Itm2a;Leprot;Srgap2;Cd200;Gmfb;Ccng1;Ccdc80;Golm1;Vamp3;Tln2;Dusp6;Myl12a;Myl12b;Ankrd13c;Map3k1;Ap2b1;Uba6;Scamp5;Atp1b1;Klf4;Dpysl2;Igf2	26
mmu-miR-494-3p	Spred1;Apcdd1;Ppp1r3b;Hmgcs1;Pnrc2;Cnbp;Acly;Gbe1;Ppargc1b;Zeb1;Sqle;Nfib;Clock;Pnisr;Cited2;Kdm7a;March7;Ube2w;Brwd3;Vegfa;Zfp871;Cpsf6;Tmem135;Fermt2;Rbm26;Rnf138	26
mmu-miR-200c-3p	Gbp6;Cited2;Gnai3;Rab14;Arhgap5;Pdk1;Brwd3;Syncrip;Sigmar1;Pcmtd1;Plxna2;Cfl2;Mtfr1;Ythdf3;Fam107b;Scd1;Ak4;Kdm7a;Sqle;4932438A13Rik;Fgd4;Zeb1;Arntl;Hectd2;Fermt2;Desi1	26
mmu-miR-200a-3p	Samd8;Sfpq;Mbtd1;Trpm7;Tmem135;Arntl;Cpsf6;Prpf4b;Ugp2;Ppp6r3;Acly;Tet2;Rab14;Nfib;Zfp280c;Gnai3;Ctnna1;Pten;Kdm7a;Slc30a1;Ddx5;Ythdf3;Wdr26;Trhde;Zfr	25
mmu-miR-200b-3p	Sigmar1;Ddx3y;Desi1;Sqle;Ak4;Fermt2;Cited2;Cfl2;Whsc1l1;Brwd3;Fam107b;Plxna2;Gbp6;Zfp770;Fgd4;Tmem135;Nfib;Rab14;Jmjd1c;Ythdf3;Mtfr1;Fam135a;Zfp871;Pcmtd1;Kdm7a	25
mmu-miR-136-5p	Igfbp2;Nfib;Rictor;Ddx5;Hmgcr;Rnf125;Taf15;Mtfr1;Kdm7a;Chic1;Ei24;Arrdc3;Zeb1;Trim2;Nisch;Ppip5k2;Ccnl2;Ube2w;Fam107b;Dmd;Exph5;Bri3bp;Dock4;4932438A13Rik	24
mmu-miR-199a-5p	Cmtm6;Ppp6r3;Maml1;Slc30a1;Chic1;Acox1;Desi1;Msmo1;Arap2;Cited2;Fzd6;Sec61a2;Ccny;Apcdd1;Brwd3;Elovl5;4932438A13Rik;Ncor1;Nagpa;Cnot6l;Phtf2;Gldc;Hmgcr;Mid1ip1	24
mmu-miR-541-5p	Fam210a;Rtn4ip1;Ahcyl2;Whsc1l1;Arhgap26;Lgalsl;Trhde;Srl;Srrm2;Mid1ip1;Nsmf;Phtf2;Zfp445;Map4;Cdc37l1;Arglu1;Rbm33;Rbm39;Sigmar1;Scd1	20
mmu-miR-382-5p	Cnot6l;Arntl;Crybg3;Syncrip;Ythdf3;Cdc37l1;Prpf4b;Fnip1;Sfpq;Tmed5;Zdhhc21;Arhgap5;Rrm2;Rab14;Rrbp1;Nfib;Eea1;Ddx5;Ddx3y;Ppp6r3	20
mmu-miR-335-5p	Serpine2;Anxa5;Stxbp1;Tox;Cpe;Sos2;Slc38a2;Btg2;Limd2;Dpysl2;Armcx2;Ctdsp2;Man2a1;Ermp1;Gpm6b;Itm2a;Adam10;Spry1;Smug1	19
mmu-miR-376c-3p	Phtf2;Ahcyl2;Ythdf3;Ccni;Sec61a2;Acly;Srsf1;Vegfa;Fubp1;Nfib;Eif2s3y;Ppp6r3;Jmjd1c;Rab14;Cmtm6;Arap2;Sorbs1;Pdia6	18
mmu-miR-182-5p	Stat1;Nr3c1;Jmjd1c;Slc30a1;Whsc1l1;Eml5;Fubp1;Zadh2;Fam69a;Slc33a1;Tnk2;Phf20l1;Fam107b;Cnot6l;Mid1ip1;Cmtm6;Fermt2;Trp53inp2	18
mmu-miR-326-3p	Gpr19;Rictor;Ptbp1;Pnrc2;Hmgcr;Cnot6l;Elovl5;Zfp655;Hnrnpa2b1;Hsd11b1;Fam126b;Fkbp3;Mapre2;Scd1;Idh1;Cnbp	16
mmu-miR-411-5p	Elac1;Gnai3;Epb4.1;Acly;Fnip1;Mtfr1;Cnbp;Evi5;Mid1ip1;Zdhhc21;Rrm2;Fzd6;Phtf2;Tmem30a;Trp53inp2	15
mmu-miR-134-5p	Pdhb;Tet2;Nudt16;Cfl2;Ahctf1;Nsmf;Paqr3;Ak3;Jmjd1c;Ctnna1;Fam107b;Zdhhc21;Hmgcr;Dnajc19	14
mmu-miR-496a-3p	Spred1;Ube2w;2310035C23Rik;Tet2;Hmgcs1;Luc7l2;Sfpq;Tmed5;Ncapd3;Ccnl2;Aqp4;Acox1;Hmgcr	13
mmu-miR-154-5p	Fam46c;Cyp51;Ppargc1b;Arntl;Evi5;Mfsd8;Fnip1;Hmgcs1;Slc33a1;Desi1;Zdhhc21	11
mmu-miR-376b-3p	Trim2;Acly;Tmem97;Syncrip;Elp4;Dpy19l3;Rictor;Ppp1r3b;Sr	10

	sf1;Pdia6	
mmu-miR-485-5p	Phtf2;Nfib;Ppargc1b;Clock;Cldn12;Btg1;Slc44a1;Ociad2;Tnk2	9
mmu-miR-379-5p	Cnot6l;Fam126b;Ergic2;Hsd17b4;Arntl;Rrm2	6
mmu-miR-300-3p	F2r;Nr6a1;Egr1;Sigmar1;Plxna2;Tet2	6
mmu-miR-345-5p	Slc6a8;Adcy7;Trp53inp1;Sirpa;Adam10	5
mmu-miR-329-3p	Ak3;Brwd3;Ccnl2;Ahctf1;Nfib	5
mmu-miR-431-5p	Ttc19;Brwd3;Sec61a2;Syncrip;4932438A13Rik	5
mmu-miR-411-3p	Rnf138;Trp53inp2	2
mmu-miR-337-3p	Tet2;Hnrnpa2b1	2
mmu-miR-486b-5p	Dynll1;Ccnd2	2
mmu-miR-434-5p	Trp53inp2;Fam126b	2
mmu-miR-409-3p	Crybg3;Gldc	2
mmu-miR-299a-5p	Srebf1	1
mmu-miR-127-3p	Spred1	1
mmu-miR-376a-3p	Trim2	1
mmu-miR-409-5p		0
mmu-miR-673-3p		0
mmu-miR-455-3p		0
mmu-miR-31-3p		0
mmu-miR-379-3p		0
mmu-miR-455-5p		0
mmu-miR-337-5p		0
mmu-miR-127-5p		0
mmu-miR-802-5p		0
mmu-miR-1193-3p		0
mmu-miR-434-3p		0
mmu-miR-484		0
mmu-miR-154-3p		0
mmu-miR-376b-5p		0
mmu-miR-540-3p		0
mmu-miR-369-3p		0
mmu-miR-136-3p		0
mmu-miR-382-3p		0
mmu-miR-485-3p		0
mmu-miR-369-5p		0
mmu-miR-339-3p		0
Total: 65 miRNAs*	499 target genes*	1,067 MTIs*,#

* indicates the unique gene count
# MTI is the abbreviation of microRNA-target interaction

Table S3.2 – Single DEG is targeted by multiple miRNAs: list of DEGs targeted by miRNA.

Target symbol	miRNAs target on this gene	Number of miRNAs regulate this gene
Nfib	miR-200a-3p;miR-494-3p;miR-376c-3p;miR-136-5p;miR-495-3p;miR-382-5p;miR-485-5p;miR-410-3p;miR-381-3p;miR-429-3p;miR-329-3p;miR-200b-3p	12
Ythdf3	miR-543-3p;miR-200a-3p;miR-200c-3p;miR-376c-3p;miR-495-3p;miR-382-5p;miR-410-3p;miR-381-3p;miR-429-3p;miR-200b-3p	10
Kdm7a	miR-543-3p;miR-200a-3p;miR-494-3p;miR-200c-3p;miR-136-5p;miR-410-3p;miR-381-3p;miR-429-3p;miR-200b-3p	9
Brwd3	miR-543-3p;miR-494-3p;miR-200c-3p;miR-495-3p;miR-431-5p;miR-199a-5p;miR-429-3p;miR-329-3p;miR-200b-3p	9

Tet2	miR-543-3p;miR-200a-3p;miR-496a-3p;miR-337-3p;miR-134-5p;miR-300-3p;miR-410-3p;miR-381-3p	8
Arntl	miR-200a-3p;miR-379-5p;miR-200c-3p;miR-154-5p;miR-495-3p;miR-382-5p;miR-381-3p;miR-429-3p	8
Rab14	miR-543-3p;miR-200a-3p;miR-200c-3p;miR-376c-3p;miR-382-5p;miR-381-3p;miR-429-3p;miR-200b-3p	8
Cnot6l	miR-543-3p;miR-379-5p;miR-326-3p;miR-382-5p;miR-199a-5p;miR-182-5p;miR-429-3p	7
Hmgcr	miR-543-3p;miR-496a-3p;miR-134-5p;miR-326-3p;miR-136-5p;miR-381-3p;miR-199a-5p	7
Trp53inp1	miR-17-5p;miR-31-5p;miR-19a-3p;miR-93-5p;miR-345-5p;miR-322-5p;miR-144-3p	7
Ube2w	miR-543-3p;miR-496a-3p;miR-494-3p;miR-136-5p;miR-495-3p;miR-410-3p;miR-381-3p	7
Syncrip	miR-543-3p;miR-376b-3p;miR-200c-3p;miR-382-5p;miR-431-5p;miR-410-3p;miR-381-3p	7
Cited2	miR-494-3p;miR-200c-3p;miR-495-3p;miR-410-3p;miR-199a-5p;miR-429-3p;miR-200b-3p	7
4932438A13Rik	miR-200c-3p;miR-136-5p;miR-495-3p;miR-431-5p;miR-381-3p;miR-199a-5p;miR-429-3p	7
Phtf2	miR-411-5p;miR-541-5p;miR-376c-3p;miR-485-5p;miR-410-3p;miR-381-3p;miR-199a-5p	7
Atp2b2	miR-17-5p;miR-31-5p;miR-19a-3p;miR-93-5p;miR-322-5p;miR-144-3p	6
Sigmar1	miR-541-5p;miR-200c-3p;miR-300-3p;miR-381-3p;miR-429-3p;miR-200b-3p	6
Mtfr1	miR-411-5p;miR-200c-3p;miR-136-5p;miR-495-3p;miR-429-3p;miR-200b-3p	6
Spred1	miR-543-3p;miR-496a-3p;miR-494-3p;miR-127-3p;miR-410-3p;miR-429-3p	6
Fam107b	miR-134-5p;miR-200c-3p;miR-136-5p;miR-182-5p;miR-429-3p;miR-200b-3p	6
Fnip1	miR-543-3p;miR-411-5p;miR-154-5p;miR-495-3p;miR-382-5p;miR-381-3p	6
Rictor	miR-326-3p;miR-376b-3p;miR-136-5p;miR-495-3p;miR-410-3p;miR-381-3p	6
Ccnd2	miR-17-5p;miR-486b-5p;miR-19a-3p;miR-93-5p;miR-322-5p;miR-145a-5p	6
Fermt2	miR-494-3p;miR-200c-3p;miR-410-3p;miR-182-5p;miR-429-3p;miR-200b-3p	6
Klf6	miR-17-5p;miR-31-5p;miR-19a-3p;miR-93-5p;miR-144-3p	5
Rrm2	miR-379-5p;miR-411-5p;miR-495-3p;miR-382-5p;miR-381-3p	5
Fubp1	miR-543-3p;miR-376c-3p;miR-495-3p;miR-410-3p;miR-182-5p	5
Whsc1l1	miR-541-5p;miR-410-3p;miR-182-5p;miR-429-3p;miR-200b-3p	5
Cfl2	miR-543-3p;miR-134-5p;miR-200c-3p;miR-429-3p;miR-200b-3p	5
Acly	miR-200a-3p;miR-411-5p;miR-494-3p;miR-376b-3p;miR-376c-3p	5
Fam126b	miR-379-5p;miR-326-3p;miR-495-3p;miR-434-5p;miR-381-3p	5
Jmjd1c	miR-134-5p;miR-376c-3p;miR-495-3p;miR-182-5p;miR-200b-3p	5
Cnbp	miR-411-5p;miR-494-3p;miR-326-3p;miR-410-3p;miR-381-3p	5
Ank2	miR-17-5p;miR-31-5p;miR-93-5p;miR-322-5p;miR-144-3p	5
Gnai3	miR-200a-3p;miR-411-5p;miR-200c-3p;miR-410-3p;miR-429	5

	-3p	
Map3k1	miR-17-5p;miR-31-5p;miR-19a-3p;miR-145a-5p;miR-144-3p	5
Trp53inp2	miR-543-3p;miR-411-3p;miR-411-5p;miR-434-5p;miR-182-5p	5
Desi1	miR-200c-3p;miR-154-5p;miR-199a-5p;miR-429-3p;miR-200b-3p	5
Ppp6r3	miR-200a-3p;miR-376c-3p;miR-495-3p;miR-382-5p;miR-199a-5p	5
Plxna2	miR-200c-3p;miR-300-3p;miR-410-3p;miR-429-3p;miR-200b-3p	5
Tob2	miR-17-5p;miR-19a-3p;miR-93-5p;miR-322-5p	4
Dpysl2	miR-17-5p;miR-335-5p;miR-93-5p;miR-145a-5p	4
Pten	miR-543-3p;miR-200a-3p;miR-495-3p;miR-410-3p	4
Btg2	miR-17-5p;miR-335-5p;miR-93-5p;miR-322-5p	4
Elovl5	miR-543-3p;miR-326-3p;miR-381-3p;miR-199a-5p	4
Adcy7	miR-17-5p;miR-19a-3p;miR-93-5p;miR-345-5p	4
Calm1	miR-19a-3p;miR-322-5p;miR-451a;miR-144-3p	4
Tox	miR-17-5p;miR-335-5p;miR-93-5p;miR-144-3p	4
Ak4	miR-200c-3p;miR-495-3p;miR-429-3p;miR-200b-3p	4
Sfpq	miR-200a-3p;miR-496a-3p;miR-382-5p;miR-410-3p	4
Zeb1	miR-494-3p;miR-200c-3p;miR-136-5p;miR-429-3p	4
Aqp4	miR-496a-3p;miR-495-3p;miR-410-3p;miR-381-3p	4
Wars	miR-17-5p;miR-31-5p;miR-322-5p;miR-144-3p	4
Hectd2	miR-200c-3p;miR-410-3p;miR-381-3p;miR-429-3p	4
Scd1	miR-541-5p;miR-326-3p;miR-200c-3p;miR-429-3p	4
Dusp6	miR-17-5p;miR-93-5p;miR-451a;miR-145a-5p	4
Ddx3y	miR-495-3p;miR-382-5p;miR-429-3p;miR-200b-3p	4
Adam10	miR-17-5p;miR-335-5p;miR-345-5p;miR-144-3p	4
Pcmtd1	miR-543-3p;miR-200c-3p;miR-429-3p;miR-200b-3p	4
Pnrc2	miR-543-3p;miR-494-3p;miR-326-3p;miR-381-3p	4
Ccng1	miR-17-5p;miR-93-5p;miR-322-5p;miR-145a-5p	4
Armc8	miR-17-5p;miR-19a-3p;miR-93-5p;miR-322-5p	4
Slc30a1	miR-200a-3p;miR-410-3p;miR-199a-5p;miR-182-5p	4
Mid1ip1	miR-411-5p;miR-541-5p;miR-199a-5p;miR-182-5p	4
Rnf38	miR-17-5p;miR-19a-3p;miR-93-5p;miR-144-3p	4
Dmd	miR-136-5p;miR-495-3p;miR-410-3p;miR-429-3p	4
Ddx5	miR-200a-3p;miR-136-5p;miR-382-5p;miR-429-3p	4
Tgfbr2	miR-17-5p;miR-19a-3p;miR-93-5p;miR-145a-5p	4
Dync1li2	miR-17-5p;miR-93-5p;miR-322-5p;miR-144-3p	4
Aebp2	miR-17-5p;miR-19a-3p;miR-93-5p;miR-144-3p	4
Crybg3	miR-543-3p;miR-382-5p;miR-381-3p;miR-409-3p	4
Zdhhc21	miR-134-5p;miR-411-5p;miR-154-5p;miR-382-5p	4
Nr3c1	miR-543-3p;miR-495-3p;miR-381-3p;miR-182-5p	4
Prpf4b	miR-200a-3p;miR-495-3p;miR-382-5p;miR-381-3p	4
G3bp2	miR-31-5p;miR-19a-3p;miR-322-5p;miR-144-3p	4
Sqle	miR-494-3p;miR-200c-3p;miR-429-3p;miR-200b-3p	4
Gpm6b	miR-17-5p;miR-335-5p;miR-19a-3p;miR-144-3p	4
Txnip	miR-17-5p;miR-31-5p;miR-93-5p;miR-322-5p	4
Id2	miR-543-3p;miR-495-3p;miR-410-3p;miR-381-3p	4
Tmem135	miR-200a-3p;miR-494-3p;miR-495-3p;miR-200b-3p	4
Sos2	miR-335-5p;miR-322-5p;miR-145a-5p	3
Armcx2	miR-335-5p;miR-19a-3p;miR-322-5p	3
Bcl6	miR-17-5p;miR-19a-3p;miR-93-5p	3
Arap2	miR-376c-3p;miR-381-3p;miR-199a-5p	3
Trpm7	miR-543-3p;miR-200a-3p;miR-381-3p	3
Hnrnpa2b1	miR-543-3p;miR-337-3p;miR-326-3p	3
Evi5	miR-543-3p;miR-411-5p;miR-154-5p	3
Rnf138	miR-411-3p;miR-494-3p;miR-381-3p	3

Dnajb9	miR-17-5p;miR-19a-3p;miR-93-5p	3
Btg1	miR-495-3p;miR-485-5p;miR-429-3p	3
Pfn2	miR-17-5p;miR-93-5p;miR-322-5p	3
Cpe	miR-17-5p;miR-335-5p;miR-93-5p	3
Sec61a2	miR-376c-3p;miR-431-5p;miR-199a-5p	3
Rab5c	miR-17-5p;miR-31-5p;miR-93-5p	3
Hmgcs1	miR-496a-3p;miR-494-3p;miR-154-5p	3
Ubfd1	miR-17-5p;miR-93-5p;miR-322-5p	3
Timp3	miR-17-5p;miR-93-5p;miR-144-3p	3
Pfkp	miR-17-5p;miR-93-5p;miR-144-3p	3
Stx6	miR-17-5p;miR-19a-3p;miR-93-5p	3
Tnfaip3	miR-17-5p;miR-19a-3p;miR-322-5p	3
Ppp1r3b	miR-543-3p;miR-494-3p;miR-376b-3p	3
Ppargc1b	miR-494-3p;miR-154-5p;miR-485-5p	3
Dock4	miR-543-3p;miR-136-5p;miR-381-3p	3
Rbm26	miR-494-3p;miR-381-3p;miR-429-3p	3
Cpsf6	miR-200a-3p;miR-494-3p;miR-495-3p	3
Clock	miR-494-3p;miR-485-5p;miR-381-3p	3
Atp8a1	miR-31-5p;miR-19a-3p;miR-144-3p	3
Man2a1	miR-335-5p;miR-322-5p;miR-451a	3
Ptgfrn	miR-17-5p;miR-93-5p;miR-144-3p	3
Tmed5	miR-496a-3p;miR-382-5p;miR-381-3p	3
App	miR-17-5p;miR-93-5p;miR-144-3p	3
Pdia6	miR-543-3p;miR-376b-3p;miR-376c-3p	3
Vamp3	miR-19a-3p;miR-145a-5p;miR-144-3p	3
Itm2a	miR-335-5p;miR-145a-5p;miR-144-3p	3
Mapre1	miR-17-5p;miR-93-5p;miR-322-5p	3
Maml1	miR-543-3p;miR-410-3p;miR-199a-5p	3
Maf1	miR-17-5p;miR-93-5p;miR-322-5p	3
Nup35	miR-17-5p;miR-19a-3p;miR-93-5p	3
Rell1	miR-17-5p;miR-19a-3p;miR-93-5p	3
Ablim1	miR-31-5p;miR-19a-3p;miR-144-3p	3
Cmtm6	miR-376c-3p;miR-199a-5p;miR-182-5p	3
Ankrd13c	miR-17-5p;miR-93-5p;miR-145a-5p	3
Mtpn	miR-17-5p;miR-322-5p;miR-144-3p	3
Fosl2	miR-19a-3p;miR-322-5p;miR-144-3p	3
Nfe2l2	miR-17-5p;miR-93-5p;miR-144-3p	3
Fgd4	miR-200c-3p;miR-429-3p;miR-200b-3p	3
Kif5b	miR-31-5p;miR-322-5p;miR-144-3p	3
Col3a1	miR-17-5p;miR-93-5p;miR-322-5p	3
Ppip5k2	miR-543-3p;miR-136-5p;miR-381-3p	3
Arhgap5	miR-200c-3p;miR-382-5p;miR-429-3p	3
Arhgap1	miR-17-5p;miR-19a-3p;miR-93-5p	3
Rbl1	miR-17-5p;miR-93-5p;miR-144-3p	3
Pafah1b2	miR-17-5p;miR-93-5p;miR-322-5p	3
Ahctf1	miR-134-5p;miR-410-3p;miR-329-3p	3
Socs3	miR-17-5p;miR-19a-3p;miR-93-5p	3
Grb10	miR-17-5p;miR-31-5p;miR-19a-3p	3
Plod2	miR-17-5p;miR-93-5p;miR-451a	3
Tnrc6c	miR-17-5p;miR-19a-3p;miR-93-5p	3
Gbp6	miR-200c-3p;miR-429-3p;miR-200b-3p	3
Vegfa	miR-494-3p;miR-376c-3p;miR-410-3p	3
Zfp871	miR-494-3p;miR-429-3p;miR-200b-3p	3
Ccnl2	miR-496a-3p;miR-136-5p;miR-329-3p	3
Tes	miR-17-5p;miR-31-5p;miR-93-5p	3
Srsf1	miR-376b-3p;miR-376c-3p;miR-381-3p	3
Id4	miR-17-5p;miR-93-5p;miR-144-3p	3
Trim2	miR-376b-3p;miR-136-5p;miR-376a-3p	3

Chic1	miR-136-5p;miR-199a-5p	2
Fbxo25	miR-17-5p;miR-93-5p	2
Otud7b	miR-19a-3p;miR-322-5p	2
Klf2	miR-31-5p;miR-144-3p	2
Sgcb	miR-19a-3p;miR-144-3p	2
Msmo1	miR-199a-5p;miR-429-3p	2
A730008H23Rik	miR-31-5p;miR-322-5p	2
Pnisr	miR-494-3p;miR-495-3p	2
7-Mar	miR-494-3p;miR-495-3p	2
Ndrg3	miR-31-5p;miR-144-3p	2
Stat1	miR-495-3p;miR-182-5p	2
Amot	miR-17-5p;miR-144-3p	2
Zbtb4	miR-17-5p;miR-93-5p	2
Avl9	miR-322-5p;miR-451a	2
Mknk2	miR-17-5p;miR-93-5p	2
Slc41a1	miR-17-5p;miR-93-5p	2
Timp2	miR-17-5p;miR-19a-3p	2
Ahcyl2	miR-541-5p;miR-376c-3p	2
4933426M11Rik	miR-17-5p;miR-93-5p	2
Trhde	miR-200a-3p;miR-541-5p	2
Ak3	miR-134-5p;miR-329-3p	2
Bmp2k	miR-17-5p;miR-322-5p	2
Nedd4l	miR-19a-3p;miR-144-3p	2
Coq10b	miR-17-5p;miR-93-5p	2
Trim35	miR-322-5p;miR-144-3p	2
Sparc	miR-322-5p;miR-451a	2
Smap1	miR-17-5p;miR-93-5p	2
Stx3	miR-17-5p;miR-93-5p	2
Igf2	miR-322-5p;miR-145a-5p	2
Ano6	miR-17-5p;miR-93-5p	2
Aak1	miR-17-5p;miR-93-5p	2
Gskip	miR-19a-3p;miR-322-5p	2
Top2a	miR-17-5p;miR-93-5p	2
Cldn12	miR-485-5p;miR-381-3p	2
Spp1	miR-17-5p;miR-93-5p	2
Acox1	miR-496a-3p;miR-199a-5p	2
Gja1	miR-19a-3p;miR-144-3p	2
Apcdd1	miR-494-3p;miR-199a-5p	2
Atp11a	miR-19a-3p;miR-144-3p	2
Rtn4	miR-322-5p;miR-144-3p	2
Rtn3	miR-17-5p;miR-322-5p	2
Plekha2	miR-19a-3p;miR-144-3p	2
Pip4k2a	miR-17-5p;miR-31-5p	2
C1ql3	miR-322-5p;miR-144-3p	2
Clic1	miR-17-5p;miR-93-5p	2
Ttc37	miR-17-5p;miR-93-5p	2
Cd200	miR-145a-5p;miR-144-3p	2
Pacs2	miR-17-5p;miR-93-5p	2
Pvr	miR-17-5p;miR-93-5p	2
Slc4a4	miR-322-5p;miR-144-3p	2
Mbtd1	miR-200a-3p;miR-495-3p	2
Rabgap1l	miR-17-5p;miR-93-5p	2
Plk3	miR-17-5p;miR-93-5p	2
Bmpr1a	miR-322-5p;miR-144-3p	2
Eea1	miR-543-3p;miR-382-5p	2
Zfp770	miR-429-3p;miR-200b-3p	2
Tgif1	miR-19a-3p;miR-144-3p	2
Npcd	miR-19a-3p;miR-322-5p	2

Tnk2	miR-485-5p;miR-182-5p	2
Ctnna1	miR-200a-3p;miR-134-5p	2
Hjurp	miR-31-5p;miR-322-5p	2
Cbx6	miR-19a-3p;miR-322-5p	2
Calm2	miR-19a-3p;miR-144-3p	2
Dcn	miR-17-5p;miR-93-5p	2
Tnfrsf21	miR-17-5p;miR-93-5p	2
Zfr	miR-200a-3p;miR-429-3p	2
Stxbp1	miR-335-5p;miR-322-5p	2
Sqstm1	miR-17-5p;miR-93-5p	2
Fam208a	miR-495-3p;miR-381-3p	2
Myl12a	miR-145a-5p;miR-144-3p	2
Rcan3	miR-17-5p;miR-93-5p	2
Jup	miR-17-5p;miR-93-5p	2
Fam63b	miR-17-5p;miR-93-5p	2
Pdgfra	miR-17-5p;miR-93-5p	2
Cdc37l1	miR-541-5p;miR-382-5p	2
Nr6a1	miR-543-3p;miR-300-3p	2
Tgfbr1	miR-17-5p;miR-144-3p	2
Pmepa1	miR-19a-3p;miR-322-5p	2
Slc38a2	miR-335-5p;miR-144-3p	2
Fam84b	miR-17-5p;miR-93-5p	2
Sparcl1	miR-17-5p;miR-93-5p	2
Wdr45b	miR-19a-3p;miR-144-3p	2
Cxcr4	miR-17-5p;miR-93-5p	2
Cav1	miR-17-5p;miR-93-5p	2
Plscr3	miR-17-5p;miR-93-5p	2
Fzd6	miR-411-5p;miR-199a-5p	2
Slc6a8	miR-19a-3p;miR-345-5p	2
Snx18	miR-17-5p;miR-93-5p	2
Cxcl12	miR-17-5p;miR-144-3p	2
Lbh	miR-31-5p;miR-19a-3p	2
Csnk1g1	miR-17-5p;miR-93-5p	2
Nptx1	miR-17-5p;miR-144-3p	2
Slc9a6	miR-19a-3p;miR-322-5p	2
Gldc	miR-199a-5p;miR-409-3p	2
Nsmf	miR-134-5p;miR-541-5p	2
Nck2	miR-17-5p;miR-93-5p	2
Slc33a1	miR-154-5p;miR-182-5p	2
Enc1	miR-19a-3p;miR-93-5p	2
Atp6v1b2	miR-19a-3p;miR-322-5p	2
Cacul1	miR-19a-3p;miR-322-5p	2
Atp1b1	miR-145a-5p;miR-144-3p	2
Jak1	miR-17-5p;miR-93-5p	2
Smug1	miR-335-5p	1
Appbp2	miR-144-3p	1
Ccny	miR-199a-5p	1
Nf2	miR-322-5p	1
4931406P16Rik	miR-31-5p	1
Ccni	miR-376c-3p	1
Alcam	miR-144-3p	1
Klf4	miR-145a-5p	1
Rbm33	miR-541-5p	1
Rbm39	miR-541-5p	1
Bcl2	miR-322-5p	1
Cyp51	miR-154-5p	1
Bri3bp	miR-136-5p	1
Tk1	miR-429-3p	1

Ncapd3	miR-496a-3p	1
Hsd11b1	miR-326-3p	1
Tnip1	miR-19a-3p	1
Sipa1l2	miR-322-5p	1
Zfp280c	miR-200a-3p	1
Fundc1	miR-145a-5p	1
Pim3	miR-17-5p	1
Zfp655	miR-326-3p	1
Wipf1	miR-31-5p	1
Ap2b1	miR-145a-5p	1
Brca1	miR-144-3p	1
Pcdhgc4	miR-451a	1
Pcdhgc5	miR-451a	1
Fam20b	miR-19a-3p	1
Dynll1	miR-486b-5p	1
Srrm2	miR-541-5p	1
Cpd	miR-322-5p	1
Rpia	miR-17-5p	1
Sorbs1	miR-376c-3p	1
11-Sep	miR-322-5p	1
Aqr	miR-144-3p	1
Srsf11	miR-495-3p	1
Epb4.1	miR-411-5p	1
Tln2	miR-145a-5p	1
Arl2	miR-322-5p	1
Ccrn4l	miR-19a-3p	1
Tmtc2	miR-322-5p	1
Rnpc3	miR-381-3p	1
Bloc1s6	miR-322-5p	1
Scamp5	miR-145a-5p	1
Ermp1	miR-335-5p	1
Ccdc80	miR-145a-5p	1
Phf20l1	miR-182-5p	1
Fam134b	miR-144-3p	1
Serpine2	miR-335-5p	1
Zfp445	miR-541-5p	1
Erlin2	miR-322-5p	1
Cbl	miR-144-3p	1
Hlf	miR-410-3p	1
B4galt6	miR-31-5p	1
Fam46c	miR-154-5p	1
Ctnnal1	miR-451a	1
Tpm4	miR-31-5p	1
Gpr124	miR-322-5p	1
Klhl2	miR-381-3p	1
Susd2	miR-31-5p	1
Arglu1	miR-541-5p	1
Gpsm3	miR-322-5p	1
Rab30	miR-17-5p	1
Wdr26	miR-200a-3p	1
Mapkapk3	miR-322-5p	1
Ildr2	miR-17-5p	1
Ei24	miR-136-5p	1
Herpud1	miR-144-3p	1
Zadh2	miR-182-5p	1
Agfg1	miR-144-3p	1
Sema3c	miR-17-5p	1
Ddit4	miR-144-3p	1

Rbm25	miR-381-3p	1
Ncor1	miR-199a-5p	1
Uba6	miR-145a-5p	1
Flnb	miR-17-5p	1
Rnf125	miR-136-5p	1
Zfp704	miR-17-5p	1
Zfp709	miR-543-3p	1
Tmed9	miR-31-5p	1
Tbc1d30	miR-495-3p	1
Eif2s3y	miR-376c-3p	1
Wtip	miR-31-5p	1
Kctd5	miR-31-5p	1
C1galt1	miR-429-3p	1
Gltp	miR-322-5p	1
Elac1	miR-411-5p	1
Scd2	miR-144-3p	1
Ergic2	miR-379-5p	1
Aldoa	miR-322-5p	1
Fam135a	miR-200b-3p	1
Clic4	miR-19a-3p	1
Abi2	miR-144-3p	1
Golm1	miR-145a-5p	1
Col4a1	miR-93-5p	1
Add1	miR-322-5p	1
Wwtr1	miR-451a	1
Itm2c	miR-17-5p	1
Taf15	miR-136-5p	1
Nacc2	miR-144-3p	1
Klhl24	miR-495-3p	1
Zfp106	miR-322-5p	1
Paqr3	miR-134-5p	1
Tmem97	miR-376b-3p	1
Clcn5	miR-19a-3p	1
Srebf1	miR-299a-5p	1
Magt1	miR-31-5p	1
Mpp1	miR-322-5p	1
Mapre2	miR-326-3p	1
Myl12b	miR-145a-5p	1
Sirpa	miR-345-5p	1
Rasal1	miR-17-5p	1
Rrbp1	miR-382-5p	1
Ccdc58	miR-543-3p	1
Pcdhgb4	miR-451a	1
Spry1	miR-335-5p	1
Tmem30a	miR-411-5p	1
Rragd	miR-17-5p	1
Slc35g1	miR-322-5p	1
Pcdhga8	miR-451a	1
Slc1a4	miR-17-5p	1
Slc1a2	miR-144-3p	1
Peg3	miR-31-5p	1
Gid4	miR-381-3p	1
Ngfrap1	miR-144-3p	1
Map4	miR-541-5p	1
Alg2	miR-322-5p	1
Pcdhgc3	miR-451a	1
Dnajc13	miR-144-3p	1
Rhob	miR-19a-3p	1

Dnajc19	miR-134-5p	1
F2r	miR-300-3p	1
Tmem56	miR-495-3p	1
Pdk1	miR-200c-3p	1
Inpp1	miR-410-3p	1
Nedd9	miR-144-3p	1
Btla	miR-322-5p	1
Cbx5	miR-322-5p	1
Sbk1	miR-31-5p	1
Onecut1	miR-495-3p	1
Zmat3	miR-17-5p	1
Slc43a2	miR-31-5p	1
Ly6e	miR-322-5p	1
Lgalsl	miR-541-5p	1
Nfe2l1	miR-322-5p	1
Gmfb	miR-145a-5p	1
Srl	miR-541-5p	1
Anxa5	miR-335-5p	1
Fam210a	miR-541-5p	1
Fam49b	miR-17-5p	1
Ikzf1	miR-144-3p	1
Acadsb	miR-495-3p	1
Itgb8	miR-17-5p	1
Ywhab	miR-31-5p	1
Ywhag	miR-322-5p	1
Ifi30	miR-31-5p	1
Srgap2	miR-145a-5p	1
Limd2	miR-335-5p	1
Pdhb	miR-134-5p	1
Pde4d	miR-144-3p	1
Ldhb	miR-19a-3p	1
Pcdhga2	miR-451a	1
Pcdhga3	miR-451a	1
Pcdhga1	miR-451a	1
Pcdhga6	miR-451a	1
Pcdhga7	miR-451a	1
Ctdsp2	miR-335-5p	1
Pcdhga5	miR-451a	1
Usp2	miR-322-5p	1
Atp6v0e	miR-93-5p	1
Rtn4ip1	miR-541-5p	1
Exph5	miR-136-5p	1
Cs	miR-19a-3p	1
Nfia	miR-381-3p	1
Igfbp2	miR-136-5p	1
Igfbp3	miR-19a-3p	1
Oat	miR-451a	1
Igfbp7	miR-17-5p	1
Kbtbd11	miR-93-5p	1
Arrdc3	miR-136-5p	1
2810403A07Rik	miR-381-3p	1
Fgfr1op2	miR-381-3p	1
Tuba1a	miR-322-5p	1
Elp4	miR-376b-3p	1
Fkbp3	miR-326-3p	1
Ociad2	miR-485-5p	1
Dpy19l3	miR-376b-3p	1
Kcnn4	miR-322-5p	1

Leprot	miR-145a-5p	1
2310061I04Rik	miR-381-3p	1
Sgk1	miR-322-5p	1
Arl8b	miR-19a-3p	1
Hsd17b4	miR-379-5p	1
AI987944	miR-495-3p	1
2310035C23Rik	miR-496a-3p	1
Gpr19	miR-326-3p	1
Mapk3	miR-322-5p	1
Vma21	miR-495-3p	1
Mapk8	miR-495-3p	1
Snx17	miR-19a-3p	1
Pcdhgb2	miR-451a	1
Pcdhgb1	miR-451a	1
Nudt16	miR-134-5p	1
Pcdhgb6	miR-451a	1
Pcdhgb5	miR-451a	1
Idh1	miR-326-3p	1
Rogdi	miR-17-5p	1
Pcdhga11	miR-451a	1
Pcdhga12	miR-451a	1
Laptm5	miR-17-5p	1
Cx3cl1	miR-322-5p	1
Eml5	miR-182-5p	1
Pcdhgb7	miR-451a	1
Thy1	miR-17-5p	1
Ttc19	miR-431-5p	1
Rreb1	miR-410-3p	1
Slc44a1	miR-485-5p	1
Pabpc1	miR-543-3p	1
Nisch	miR-136-5p	1
Samd8	miR-200a-3p	1
Ugp2	miR-200a-3p	1
Luc7l2	miR-496a-3p	1
Pcdhgb8	miR-451a	1
Egr1	miR-300-3p	1
Smoc2	miR-17-5p	1
Nol4l	miR-322-5p	1
Entpd7	miR-17-5p	1
Eln	miR-144-3p	1
Zfp800	miR-543-3p	1
Zkscan1	miR-381-3p	1
Msn	miR-144-3p	1
Brms1l	miR-17-5p	1
Ptbp1	miR-326-3p	1
Pard6b	miR-322-5p	1
Col4a2	miR-17-5p	1
BC029722	miR-93-5p	1
Pcdhga10	miR-451a	1
Clpx	miR-543-3p	1
Pcdhga4	miR-451a	1
Mfsd8	miR-154-5p	1
Thbd	miR-451a	1
Cotl1	miR-31-5p	1
Atp10a	miR-19a-3p	1
Lims1	miR-322-5p	1
Nagpa	miR-199a-5p	1
Prkcb	miR-19a-3p	1

Tom1l2	miR-144-3p	1
Fam69a	miR-182-5p	1
Gbe1	miR-494-3p	1
Mvb12b	miR-31-5p	1
Arhgap26	miR-541-5p	1
Total: 499 target genes*	44 miRNAs*	1,067 MTIs*,#

* indicates the unique gene count

MTI is the abbreviation of microRNA-target interaction

Table S4.1 – Single miRNA co-targets multiple miR-122a target genes: list of miRNA co-targeted to miR-122a target genes. (We selected the miR-122a target genes with ≥ 1.5 fold change in expression)

miRNA	co-miR122 targets	Number of shared miR-122a targets
mmu-miR-144-3p	Klf6;Adam10;Gpm6b;G3bp2;Aebp2;Vamp3;Pfkp;Timp3;Ndrg3;Rtn4;Atp1b1;Cxcl12;Trim35;Tgfbr1;Bmpr1a;Scd2;Ikzf1;Aqr;Cbl	19
mmu-miR-17-5p	Klf6;Ccng1;Adam10;Gpm6b;Aebp2;Rell1;Pfkp;Pfn2;Timp3;Clic1;Cxcl12;Slc41a1;Tgfbr1;Tnfrsf21;Rragd;Col4a2;Rpia	17
mmu-miR-93-5p	Klf6;Ccng1;Aebp2;Rell1;Pfkp;Pfn2;Timp3;Clic1;Slc41a1;Tnfrsf21;BC029722;Atp6v0e	12
mmu-miR-322-5p	Ccng1;G3bp2;Pfn2;Rtn4;Trim35;Bmpr1a;Aldoa;Arl2;Btla;Tuba1a	10
mmu-miR-19a-3p	Klf6;Gpm6b;G3bp2;Aebp2;Vamp3;Rell1;Ccrn4l;Cs	8
mmu-miR-31-5p	Klf6;G3bp2;Ndrg3;Tpm4;Sbk1	5
mmu-miR-145a-5p	Ccng1;Vamp3;Atp1b1;Gmfb	4
mmu-miR-335-5p	Adam10;Gpm6b	2
mmu-miR-345-5p	Adam10	1
Total: 9 miRNAs*	39 co-miR-122a targets*	

* indicates the unique gene count

Table S4.2 – Single miR-122a target gene is targeted by multiple miRNAs: list miR-122a target genes targeted by other miRNA. (We selected the miR-122a target genes with ≥ 1.5 fold change in expression)

Gene	Fold change	Co-miRs	Number of co-miRs target to this gene
Klf6	2.88	miR-17-5p;miR-31-5p;miR-19a-3p;miR-93-5p;miR-144-3p	5
Ccng1	2.49	miR-17-5p;miR-93-5p;miR-322-5p;miR-145a-5p	4
Adam10	2.01	miR-17-5p;miR-335-5p;miR-345-5p;miR-144-3p	4
Gpm6b	1.95	miR-17-5p;miR-335-5p;miR-19a-3p;miR-144-3p	4
G3bp2	1.64	miR-31-5p;miR-19a-3p;miR-322-5p;miR-144-3p	4
Aebp2	1.51	miR-17-5p;miR-19a-3p;miR-93-5p;miR-144-3p	4
Vamp3	4.60	miR-19a-3p;miR-145a-5p;miR-144-3p	3
Rell1	1.98	miR-17-5p;miR-19a-3p;miR-93-5p	3
Pfkp	1.72	miR-17-5p;miR-93-5p;miR-144-3p	3
Pfn2	1.65	miR-17-5p;miR-93-5p;miR-322-5p	3
Timp3	1.53	miR-17-5p;miR-93-5p;miR-144-3p	3
Ndrg3	3.75	miR-31-5p;miR-144-3p	2
Rtn4	2.97	miR-322-5p;miR-144-3p	2
Atp1b1	2.30	miR-145a-5p;miR-144-3p	2
Clic1	2.20	miR-17-5p;miR-93-5p	2
Cxcl12	2.20	miR-17-5p;miR-144-3p	2
Slc41a1	1.99	miR-17-5p;miR-93-5p	2
Trim35	1.68	miR-322-5p;miR-144-3p	2
Tgfbr1	1.55	miR-17-5p;miR-144-3p	2
Bmpr1a	1.53	miR-322-5p;miR-144-3p	2
Tnfrsf21	1.50	miR-17-5p;miR-93-5p	2
Ccrn4l	6.91	miR-19a-3p	1
Aldoa	4.00	miR-322-5p	1
Rragd	3.49	miR-17-5p	1
Tpm4	3.43	miR-31-5p	1
Sbk1	2.30	miR-31-5p	1

Arl2	2.30	miR-322-5p	1
BC029722	2.15	miR-93-5p	1
Cs	1.97	miR-19a-3p	1
Scd2	1.84	miR-144-3p	1
Atp6v0e	1.84	miR-93-5p	1
Gmfb	1.77	miR-145a-5p	1
Btla	1.73	miR-322-5p	1
Col4a2	1.73	miR-17-5p	1
Tuba1a	1.69	miR-322-5p	1
Rpia	1.66	miR-17-5p	1
Ikzf1	1.58	miR-144-3p	1
Aqr	1.54	miR-144-3p	1
Cbl	1.51	miR-144-3p	1
Total: 39 miR-122a targets* / 9 miRNAs*			

* indicates the unique gene count

Table S5.1 – IPA analysis of 204 down-regulated target genes of UPmiRs and 295 up-regulated target genes of DNmiRs in the category of "Diseases and Functions".

Diseases and Functions	UPmiRs			DNmiRs		
	p value	Molecules	Count	*p* value	Molecules	Count
Lipid Metabolism	3.85E-06	SCD,SIGMAR1,ARNTL,EGR1,ACOX1,MAPK8,ACLY,NR3C1,ELOVL5,IDH1,PTEN,GNAI3,EPB41,SORBS1,SREBF1,CLOCK,MID1IP1,PPARGC1B,HMGCR,HSD17B4,CYP51A1,HSD11B1	22			
Cellular Growth and Proliferation	9.18E-06	ID2,F2R,CTNNA1,DOCK4,DMD,NR3C1,PTEN,VEGFA,ARRDC3,TET2,NSMF,NCOR1,STAT1,CITED2,ARNTL,PAQR3,EGR1,MAPK8,ZEB1,IGFBP2,ARHGAP5,NFIA,APCDD1,NFIB,C1GALT1,HMGCR	26	5.17E-10	SOCS3,TGFBR1,JAK1,PLK3,KLF6,CXCL12,NF2,RBL1,BCL6,TGFBR2,CCNG1,RHOB,MAPK3,CAV1,IFI30,LEPROT,SRGAP2,JUP,BRCA1,FOSL2,KLF2,TIMP2,TNFRSF21,ELN,GJA1,TP53INP1,SPP1,YWHAG,DCN,TRIM35,KLF4,IGF2,CBL,CCND2,TLN2,BTG2,IGFBP3,RTN4,ALCAM,ENC1,ARHGAP1,SIRPA,ABI2,PIM3,KCNN4,LAPTM5,TNFAIP3,COL4A2,IGFBP7,TGIF1,BCL2,ROGDI,ZMAT3,BMPR1A,PDGFRA,Pvr,NFE2L2,BTLA,SPRY1,CXCR4,TXNIP,IKZF1,MAP3K1,APP,GRB10,GOLM1,SERPINE2,WIPF1,LIMS1,ADAM10,SPARC,SLC1A2,PLEKHA2,NEDD9,TOX,ID4,PRKCB	77
Connective Tissue Disorders	1.12E-04	VEGFA,SCD,SPRED1,SREBF1,ACOX1,EGR1,MAPK8,C1GALT1,NR3C1,HSD11B1,PTEN	11			
Energy Production	5.59E-04	SCD,ACOX1,PPARGC1B,HSD17B4,HSD11B1	5			
Metabolic Disease	8.11E-04	SCD,SREBF1,ACOX1,MAPK8,PPARGC1B,NR3C1,HSD11B1,IDH1	8			
RNA Post-Transcriptional Modification	1.10E-03	PABPC1,DDX5,SYNCRIP	3			
Nervous System Development and Function	1.57E-03	NR6A1,ID2,DOCK4,DMD,RICTOR,NR3C1,PTEN,VEGFA,CFL2,CLOCK,NCOR1,NSMF,CITED2,ARNTL,	25	5.27E-28	SOCS3,GPR124,PCDHGB1,PCDHGB7,CXCL12,PCDHGC3,PCDHGA3,RBL1,RHOB,PACS2,CAV1,BRCA1,PCDHGB6,TNFRSF21,PEG3,SLC6A8,GJA	61

		EGR1,MAPK8,SYNCRIP,ZEB1,ARHGAP5,EPB41,MBTD1,NFIA,FZD6,NFIB,CNBP			1,PCDHGA11,CD200,SPP1,PCDHGA10,PCDHGA6,Ank2,OAT,PCDHGC5,PCDHGB3,PCDHGA1,PCDHGC4,SPARCL1,IGF2,CCND2,RTN4,ALCAM,SIRPA,ABI2,PCDHGB2,PCDHGA12,PCDHGB4,PCDHGA8,ITGB8,TGIF1,BCL2,BMPR1A,PFN2,NFE2L2,GMFB,PCDHGA7,CXCR4,IKZF1,PCDHGA4,PCDHGB5,PCDHGA2,GPM6B,PCDHGA5,ATP2B2,SERPINE2,APP,ADAM10,SLC1A2,SPARC,PRKCB	
Tissue Development	1.60E-03	NR6A1,ID2,F2R,CTNNA1,DOCK4,DMD,NR3C1,PTEN,VEGFA,PPARGC1B,TET2,NSMF,NCOR1,STAT1,HSD17B4,CITED2,CYP51A1,TK1,ARNTL,PAQR3,EGR1,MAPK8,ZEB1,ZDHHC21,ONECUT1,IGFBP2,ARHGAP5,MBTD1,SREBF1,NFIA,FZD6,NFIB,HSD11B1,CNBP	34	6.33E-29	SOCS3,PCDHGB1,JAK1,TGFBR1,PCDHGB7,CXCL12,NF2,PCDHGC3,PCDHGA3,BCL6,RBL1,TGFBR2,CCNG1,DYNLL1,RHOB,MAPK3,CAV1,IFI30,LEPROT,JUP,BRCA1,FOSL2,PCDHGB6,KLF2,TIMP2,ELN,PCDHGA11,GJA1,COL4A1,PCDHGA10,PCDHGA6,CD200,YWHAG,SPP1,Ank2,PCDHGB3,DCN,PCDHGC5,SGCB,PCDHGA1,KLF4,PCDHGC4,TOB2,IGF2,CBL,TLN2,CCND2,RTN4,IGFBP3,SQSTM1,CX3CL1,ARHGAP1,SIRPA,ABI2,FLNB,SMAP1,PCDHGA12,PCDHGB2,CLIC1,WWTR1,PCDHGB4,PCDHGA8,COL4A2,ITGB8,IGFBP7,CLIC4,TGIF1,BCL2,ALG2,BMPR1A,ANXA5,PDGFRA,LY6E,NFE2L2,TIMP3,PCDHGA7,CXCR4,TXNIP,IKZF1,MAP3K1,PCDHGA4,PCDHGA2,PCDHGB5,MAN2A1,PCDHGA5,NFE2L1,SERPINE2,APP,ATP2B2,GRB10,LIMS1,ADAM10,ADD1,SPARC,LBH,NEDD9	96
Cell-To-Cell Signaling and Interaction	1.64E-03	VEGFA,EPB41,SCD,ARNTL,F2R,EGR1,CTNNA1,MAPK8,RICTOR,C1GALT1,NR3C1,PTEN	12	6.33E-29	JAK1,PCDHGB1,PCDHGB7,NF2,CXCL12,PCDHGC3,PCDHGA3,TGFBR2,RHOB,JUP,KLF2,PCDHGB6,TIMP2,GJA1,PCDHGA11,PCDHGA10,PCDHGA6,SPP1,CD200,PCDHGB3,PCDHGC5,DCN,PCDHGA1,PCDHGC4,IGF2,CBL,SIRPA,LAPTM5,PCDHGA12,PCDHGB2,CLIC1,PCDHGB4,PCDHGA8,BCL2,BLOC1S6,NFE2L2,BTLA,PCDHGA7,TXNIP,IKZF1,MAP3K1,PCDHGA4,PCDHGA2,PCDHGB5,MAN2A1,PCDHGA5,SERPINE2,ATP2B2,APP,LIMS1,ADAM10,SPARC,NEDD9,PRKCB	54

Cell Death and Survival	4.27E-03	VEGFA,SCD,SIGMAR1,ID2,EGR1,CLOCK,CTNNA1,MAPK8,STAT1,NR3C1,IGFBP2,PTEN	12	4.85E-24	TGFBR1,PCDHGB1,PLK3,SGK1,PCDHGB7,PCDHGC3,RBL1,NCK2,CCNG1,DYNLL1,CAV1,TNFRSF21,TP53INP1,PCDHGA10,YWHAG,DDIT4,DCN,THBD,MTPN,CCND2,RTN4,ALCAM,ALDOA,ARHGAP1,DUSP6,ITGB8,COL4A2,IGFBP7,PDGFRA,TOP2A,AMOT,NFE2L2,PCDHGA7,MAPKAPK3,TXNIP,IKZF1,PCDHGB5,GPM6B,APP,LIMS1,ADD1,PRKCB,SOCS3,CXCL12,BCL6,PCDHGA3,TGFBR2,TNIP1,RHOB,MAPK3,PACS2,JUP,BRCA1,NEDD4L,PCDHGB6,KLF2,SLC6A8,PEG3,PCDHGA11,STXBP1,GJA1,PCDHGA6,CD200,SPP1,COL4A1,PCDHGB3,PCDHGC5,SGCB,PCDHGA1,TRIM35,PCDHGC4,SMUG1,IGF2,CBL,IGFBP3,SIRPA,PAFAH1B2,KCNN4,PCDHGB2,PCDHGA12,PCDHGB4,TNFAIP3,PCDHGA8,USP2,CLIC4,BCL2,BMPR1A,Pvr,GMFB,BTLA,TIMP3,CXCR4,NGFRAP1,FAM134B,MAP3K1,HERPUD1,PCDHGA4,PCDHGA2,MAN2A1,PCDHGA5,NFE2L1,SERPINE2,SLC1A2,SPARC,TOX	105
Cellular Assembly and Organization	4.68E-03	SIGMAR1,SCD,CTNNA1,MAPK8,DOCK4,DMD,RICTOR,NR3C1,FNIP1,PTEN,VEGFA,ARHGAP5,EPB41,NFIA,PPARGC1B,NSMF,C1GALT1	17	6.33E-29	RAB5C,TGFBR1,PCDHGB1,PCDHGB7,NF2,CXCL12,PCDHGC3,PCDHGA3,BCL6,NCK2,DYNLL1,RHOB,MAPK3,CAV1,SRGAP2,JUP,PCDHGB6,KLF2,PCDHGA11,GJA1,PCDHGA6,PCDHGA10,SPP1,Ank2,PCDHGB3,DCN,PCDHGC5,PCDHGA1,THBD,PCDHGC4,CBL,DYNC1LI2,RTN4,SIRPA,COL3A1,ABI2,PCDHGA12,PCDHGB2,WWTR1,PCDHGB4,STX6,PCDHGA8,BCL2,PFN2,BLOC1S6,AMOT,AGFG1,PCDHGA7,CXCR4,MAPRE1,PCDHGA4,PCDHGB5,PCDHGA2,MAN2A1,PCDHGA5,GOLM1,ATP2B2,APP,SERPINE2,WIPF1,KIF5B,LIMS1,ADAM10,SPARC,RTN3	65
Cellular Movement	6.92E-03	VEGFA,Nisch,SREBF1,NFIA,STAT1,PTEN	6	4.52E-09	SOCS3,JAK1,TGFBR1,CXCL12,NCK2,TGFBR2,RHOB,CAV1,JUP,FOSL2,BRCA1,KLF2,TIMP2,TNFRSF21,ELN,TP53INP1,GJA1,SPP1,COL4A1,CD200,DCN,THBD,IGF2,CBL,BTG2,IGFBP3,RTN4,AL	58

					CAM,ARHGAP1,CX3CL1,SIRPA,COL3A1,FLNB,TNFAIP3,ITGB8,USP2,CLIC4,BCL2,BMPR1A,Pde4d,Pvr,NFE2L2,AMOT,MYL12A,BTLA,TIMP3,CXCR4,MAPRE1,MAP3K1,MPP1,APP,SERPINE2,WIPF1,ADAM10,SLC1A2,SPARC,SEMA3C,NEDD9	
Cell Cycle	1.06E-02	ID2,EGR1,ZEB1,PTEN	4	2.28E-07	Calm1,TGFBR1,PLK3,CXCL12,IGFBP7,RBL1,BCL6,TGIF1,BCL2,TGFBR2,CCNG1,RHOB,MAPK3,CAV1,PDGFRA,Pvr,BRCA1,NFE2L2,GJA1,TP53INP1,SPP1,IKZF1,MAP3K1,KLF4,APP,GRB10,IGF2,TOB2,CCND2,CBL,SPARC,ID4	32
Organismal Survival				1.82E-11	PCDHGB1,TGFBR1,PLK3,PCDHGB7,NF2,PCDHGC3,RBL1,CCNG1,NCK2,DYNLL1,CAV1,TP53INP1,PCDHGA10,Ank2,OAT,DCN,THBD,CCND2,RTN4,SQSTM1,ARHGAP1,COL3A1,SLC4A4,FLNB,COL4A2,ITGB8,TOP2A,PDGFRA,Pde4d,LY6E,AMOT,NFE2L2,PCDHGA7,MAPKAPK3,SPRY1,TXNIP,IKZF1,PCDHGB5,GPM6B,APP,LIMS1,ADAM10,ADD1,PRKCB,SOCS3,GPR124,JAK1,KLF6,CXCL12,BCL6,PCDHGA3,TGFBR2,TNIP1,MAPK3,JUP,FOSL2,BRCA1,NEDD4L,PCDHGB6,KLF2,ELN,PCDHGA11,GJA1,STXBP1,CD200,COL4A1,SPP1,PCDHGA6,PCDHGC5,PCDHGB3,UBA6,PCDHGA1,KLF4,PCDHGC4,IGF2,CBL,PCDHGA12,PCDHGB2,PCDHGB4,TNFAIP3,PCDHGA8,CLIC4,TGIF1,BCL2,BMPR1A,BTLA,TIMP3,CXCR4,MAP3K1,Scd2,PCDHGA4,MAN2A1,PCDHGA2,DNAJB9,NFE2L1,PCDHGA5,KIF5B,SLC1A2,ADCY7	99

Total number of UPmiR target genes: 57
Total number of DNmiR target genes: 168

Table S5.2 –IPA analysis of 204 down-regulated target genes of UPmiRs and 295 up-regulated target genes of DNmiRs in the category of “Canonical Pathways”.

Ingenuity Canonical Pathways	UPmiRs			DNmiRs		
	p value	Molecules	Count	*p* value	Molecules	Count
Superpathway of Cholesterol Biosynthesis	8.51E-06	SQLE,MSMO1,HMGCR,HMGCS1,CYP51A1	5			
Glycogen Biosynthesis II (from UDP-D-Glucose)	1.10E-03	UGP2,GBE1	2			
Pyrimidine Deoxyribonucleotides De Novo Biosynthesis I	2.09E-02	RRM2,AK4	2			
Regulation of the Epithelial-Mesenchymal Transition Pathway	4.57E-02	MAML1,ID2,EGR1,FZD6,ZEB1	5	7.76E-02	TGFBR2,JAK1,TGFBR1,MAPK3,SOS2,PARD6B	6
Leukocyte Extravasation Signaling	5.50E-02	ARHGAP5,GNAI3,CLDN12,MAPK8,CTNNA1	5	4.17E-03	TIMP3,WIPF1,CXCR4,CXCL12,THY1,ARHGAP1,MSN,TIMP2,PRKCB	9
Integrin Signaling	5.50E-02	ARHGAP5,ARHGAP26,MAPK8,TNK2,PTEN	5	7.59E-05	NCK2,WIPF1,TLN2,RHOB,LIMS1,MAPK3,SOS2,CAV1,ITGB8,NEDD9,MYL12B,MYL12A	12
PDGF Signaling	1.98E-01	MAPK8,STAT1	2	2.51E-04	JAK1,MAPK3,SOS2,MAP3K1,PDGFRA,CAV1,PRKCB	7
CXCR4 Signaling	2.12E-01	GNAI3,EGR1,MAPK8	3	3.02E-03	RHOB,CXCR4,MAPK3,CXCL12,ADCY7,MYL12B,PRKCB,MYL12A	8
PAK Signaling	2.29E-01	CFL2,MAPK8	3	2.57E-03	NCK2,MAPK3,SOS2,PDGFRA,MYL12B,MYL12A	6
p53 Signaling	2.64E-01	MAPK8,PTEN	3	4.27E-03	CCNG1,TP53INP1,CCND2,BRCA1,BCL2,SERPINE2	6
IGF-1 Signaling	2.64E-01	MAPK8,IGFBP2	3	2.19E-05	SOCS3,YWHAG,JAK1,YWHAB,MAPK3,SOS2,IGFBP3,IGFBP7,GRB10	9
p70S6K Signaling	3.54E-01	GNAI3,F2R	3	1.20E-02	YWHAG,JAK1,YWHAB,MAPK3,SOS2,PRKCB	6
RhoA Signaling	3.54E-01	ARHGAP5,CFL2	3	2.95E-03	PFN2,SEPT11,PIP4K2A,ARHGAP1,MYL12B,MSN,MYL12A	7
Axonal Guidance Signaling	4.68E-01	VEGFA,GNAI3,CFL2,FZD6,PLXNA2	5	5.50E-04	DPYSL2,CXCR4,SOS2,CXCL12,ABLIM1,NCK2,WIPF1,TUBA1A,MAPK3,ADAM10,RTN4,PFN2,SRGAP2,SEMA3C,MYL12B,PRKCB,MYL1	17

					2A	
Molecular Mechanisms of Cancer	5.28E-01	GNAI3,MAPK8,CTNNA1,FZD6	4	2.14E-03	JAK1,TGFBR1,SOS2,RBL1,BCL2,TGFBR2,CCND2,CBL,RHOB,BMPR1A,MAPK3,BRCA1,ADCY7,PRKCB	14
NRF2-mediated Oxidative Stress Response	5.42E-01	DNAJC19,MAPK8	2	1.86E-03	MAPK3,MAP3K1,DNAJC13,HERPUD1,SQSTM1,DNAJB9,NFE2L2,ENC1,PRKCB	9
STAT3 Pathway	5.42E-01	MAPK8	1	1.78E-04	TGFBR2,SOCS3,TGFBR1,BMPR1A,MAPK3,PDGFRA,BCL2	7
TGF-β Signaling	5.98E-01	MAPK8	1	7.08E-05	TGFBR2,TGFBR1,BMPR1A,MAPK3,SOS2,TGIF1,PMEPA1,BCL2	8
Glioma Signaling	6.35E-01	PTEN	1	8.32E-04	IGF2,Calm1 (includes others),MAPK3,SOS2,PDGFRA,RBL1,PRKCB	7
Actin Cytoskeleton Signaling				6.76E-03	ABI2,TLN2,MAPK3,SOS2,PFN2,PIP4K2A,MYL12B,MSN,MYL12A	9
Hepatic Fibrosis / Hepatic Stellate Cell Activation				2.82E-04	TGFBR2,IGF2,COL4A1,TGFBR1,KLF6,PDGFRA,IGFBP3,COL4A2,COL3A1,TIMP2,BCL2	11
Protein Kinase A Signaling				9.33E-04	FLNB,YWHAG,Calm1,TGFBR1,YWHAB,DUSP6,MAP3K1,TGFBR2,MAPK3,ADD1,ADCY7,MYL12B,SIRPA,MYL12A,PRKCB	15
ERK/MAPK Signaling				8.91E-03	TLN2,YWHAG,YWHAB,DUSP6,MAPK3,SOS2,MKNK2,PRKCB	8
PTEN Signaling				6.03E-04	TGFBR2,TGFBR1,CBL,BMPR1A,MAPK3,SOS2,PDGFRA,BCL2	8
NF-κB Signaling				5.37E-03	TGFBR2,TNIP1,TGFBR1,BMPR1A,MAP3K1,PDGFRA,TNFAIP3,PRKCB	8
Regulation of Actin-based Motility by Rho				2.45E-03	WIPF1,RHOB,PFN2,PIP4K2A,MYL12B,MYL12A	6
Cell Cycle: G2/M DNA Damage Checkpoint Regulation				8.13E-03	YWHAG,YWHAB,TOP2A,BRCA1	4

Total number of UPmiR target genes: 29
Total number of DNmiR target genes: 76

Figure S1. The proportion of different types of RNA molecules obtained from the small RNA sequencing data.

Figure S2.1. Causal Network Analysis (CNA) in the lipid metabolism of down-regulated genes. Downstream target molecules are shown here with the expression color code. Blue color coded node indicates that CNA predicted that node is “inhibited” based on the gene expression changes from the experimental results. In contract, node is color coded in orange. The relationship is color-coded based on the predicted relationship as indicated in the legend.

Figure S2.2. Causal Network Analysis in the cellular growth and proliferation of down-regulated genes.

Figure S2.3. Causal Network Analysis in the cellular growth and proliferation of up-regulated genes.

Figure S2.4. Causal Network Analysis of the connective tissue disorders with down-regulated genes.

Figure S2.5. Causal Network Analysis of the energy production with down-regulated genes.

Figure S2.6. Causal Network Analysis of the metabolic disease with down-regulated genes.

Figure S2.7. Causal Network Analysis of the RNA post-transcriptional modification with down-regulated genes.

Figure S2.8. Causal Network Analysis of the nervous system development and function with down-regulated genes.

Figure S2.9. Causal Network Analysis of the nervous system development and function with up-regulated genes.

Figure S2.10. Causal Network Analysis of the tissue development with down-regulated genes.

Figure S2.11. Causal Network Analysis of the tissue development with up-regulated genes.

Figure S2.12. Causal Network Analysis of the cell-to-cell signaling and interaction with down-regulated genes.

Figure S2.13. Causal Network Analysis of the ell-to-cell signaling and interaction with up-regulated genes.

Figure S2.14. Causal Network Analysis of the cell death and survival with down-regulated genes.

Figure S2.15. Causal Network Analysis of the cell death and survival with up-regulated genes.

Figure S2.16. Causal Network Analysis of the cellular assembly and organization with down-regulated genes.

Figure S2.17. Causal Network Analysis of the cellular assembly and organization with up-regulated genes.

Figure S2.18. Causal Network Analysis of the cellular movement with down-regulated genes.

Figure S2.19. Causal Network Analysis of the cellular movement with up-regulated genes.

Figure S2.20. Causal Network Analysis of the cell cycle with down-regulated genes.

Figure S2.21. Causal Network Analysis of the cell cycle with up-regulated genes.

Figure S2.22. Causal Network Analysis of the organismal survival with up-regulated genes.

Figure S3.1. Ingenuity canonical pathways enriched in Regulation of the Epithelial-Mesenchymal Transition Pathway.
Figure S3.2. Ingenuity canonical pathways enriched in Leukocyte Extravasation Signaling.
Figure S3.3. Ingenuity canonical pathways enriched in Integrin Signaling.
Figure S3.4. Ingenuity canonical pathways enriched in PDGF Signaling.
Figure S3.5. Ingenuity canonical pathways enriched in CXCR4 Signaling.
Figure S3.6. Ingenuity canonical pathways enriched in PAK Signaling.
Figure S3.7. Ingenuity canonical pathways enriched in IGF-1 Signaling.
Figure S3.8. Ingenuity canonical pathways enriched in RhoA Signaling.
Figure S3.9. Ingenuity canonical pathways enriched in Axonal Guidance Signaling.
Figure S3.10. Ingenuity canonical pathways enriched in Molecular Mechanisms of Cancer.
Figure S3.11. Ingenuity canonical pathways enriched in NRF2-mediated Oxidative Stress Response.
Figure S3.12. Ingenuity canonical pathways enriched in TGF-β Signaling.
Figure S3.13. Ingenuity canonical pathways enriched in Glioma Signaling.
Figure S3.14. Ingenuity canonical pathways enriched in Actin Cytoskeleton Signaling.
Figure S3.15. Ingenuity canonical pathways enriched in Hepatic Fibrosis.
Figure S3.16. Ingenuity canonical pathways enriched in ERK/MAPK Signaling.
Figure S3.17. Ingenuity canonical pathways enriched in PTEN Signaling.
Figure S3.18. Ingenuity canonical pathways enriched in Regulation of Actin-based Motility by Rho.
Figure S3.19. Ingenuity canonical pathways enriched in Cell Cycle: G2/M DNA Damage Checkpoint Regulation.

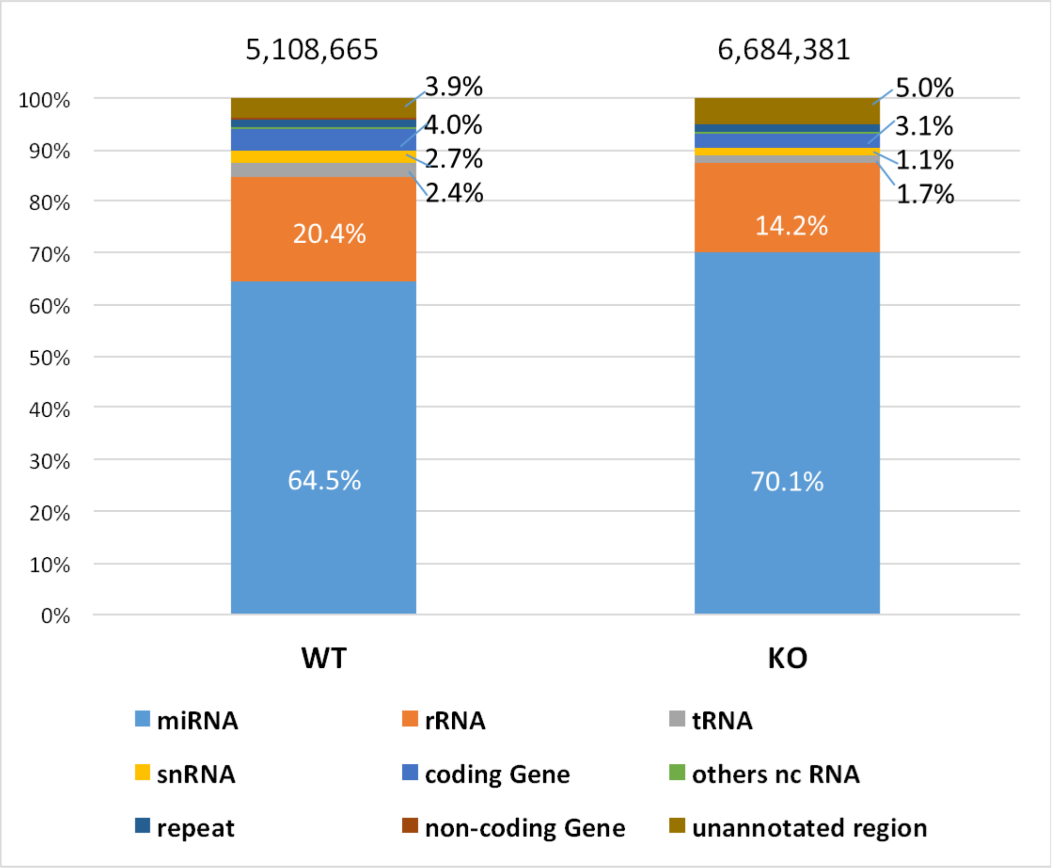

Figure S1. The proportion of different types of RNA molecules obtained from the small RNA sequencing data.

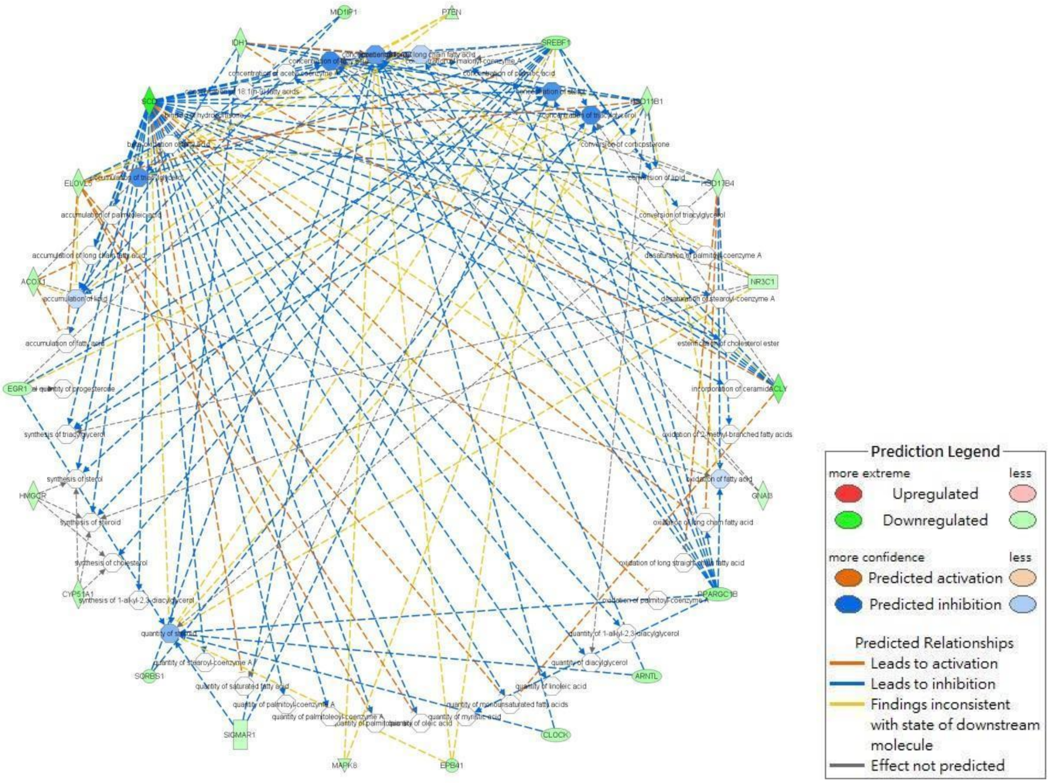

Figure S2.1. Causal Network Analysis (CNA) in the lipid metabolism of down-regulated genes. Downstream target molecules are shown here with the expression color code. Blue color coded node indicates that CNA predicted that node is “inhibited” based on the gene expression changes from the experimental results. In contract, node is color coded in orange. The relationship is color-coded based on the predicted relationship as indicated in the legend.

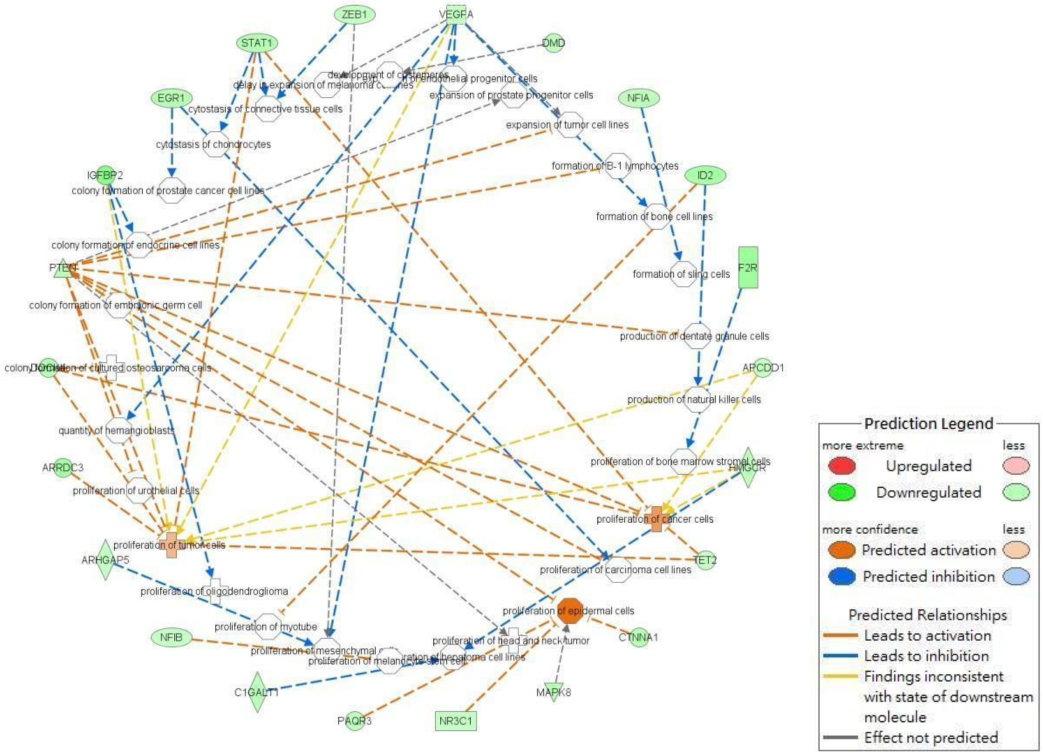

Figure S2.2. Causal Network Analysis in the cellular growth and proliferation of down-regulated genes.

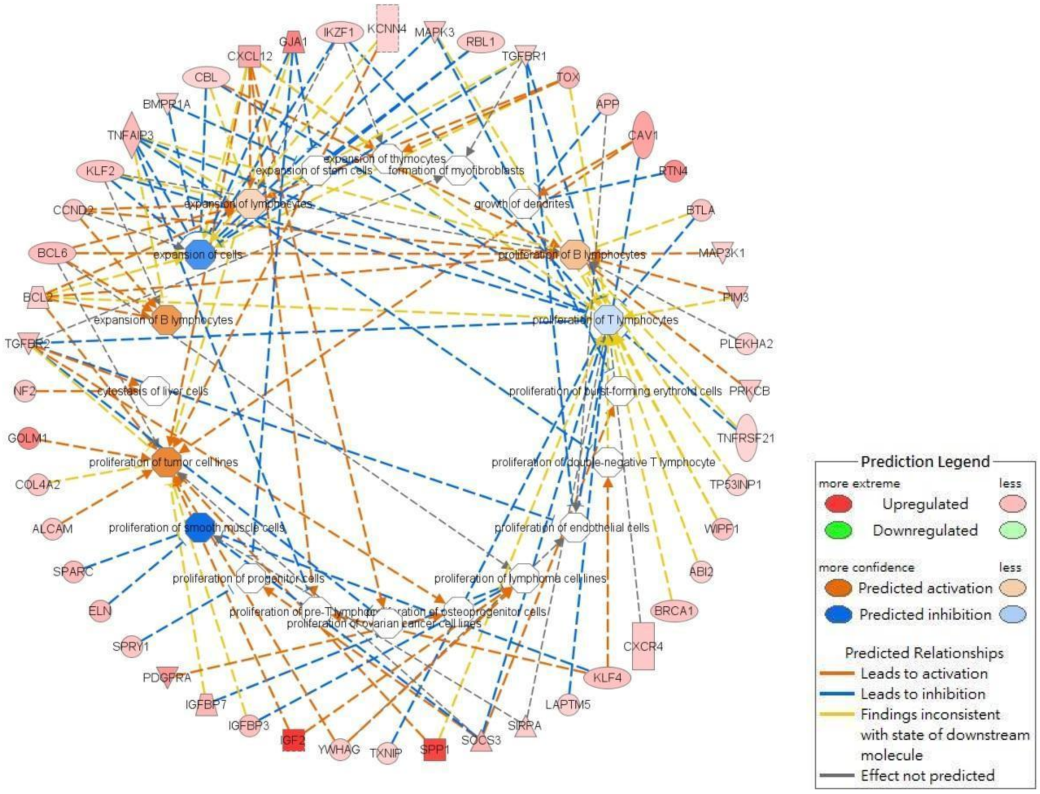

Figure S2.3. Causal Network Analysis in the cellular growth and proliferation of up-regulated genes.

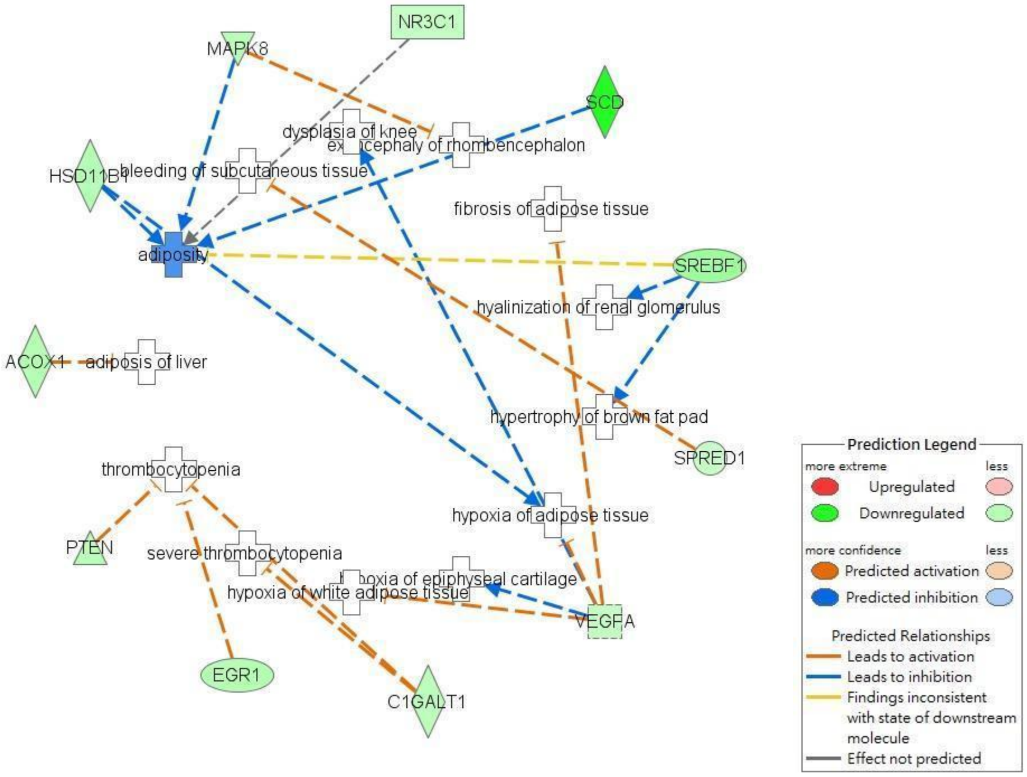

Figure S2.4. Causal Network Analysis of the connective tissue disorders with down-regulated genes.

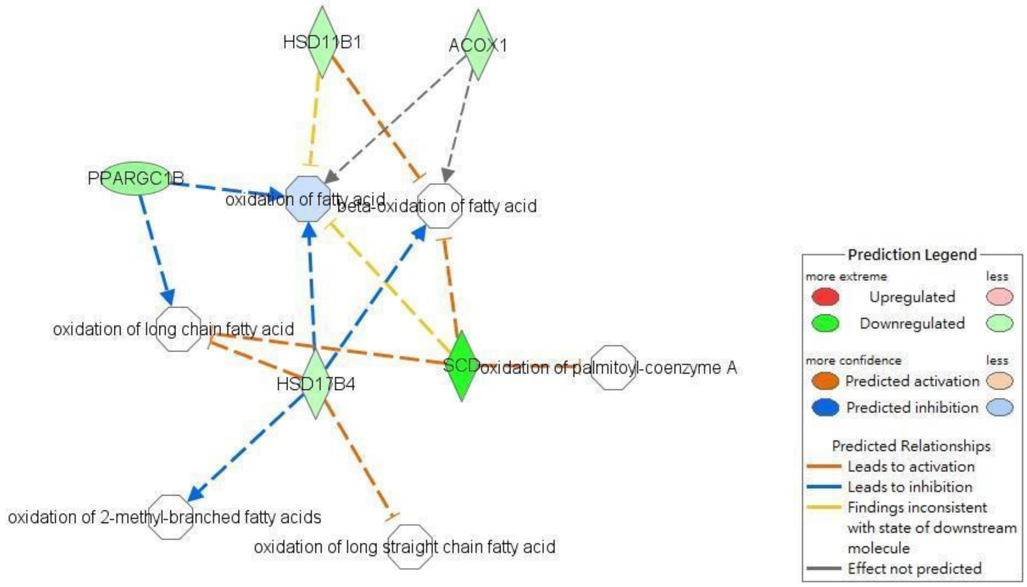

Figure S2.5. Causal Network Analysis of the energy production with down-regulated genes.

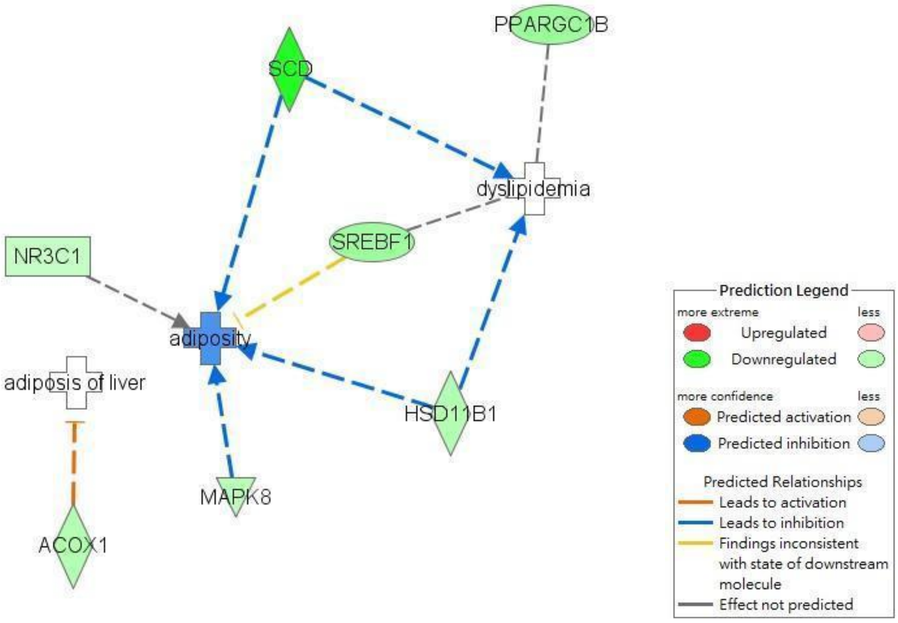

Figure S2.6. Causal Network Analysis of the metabolic disease with down-regulated genes.

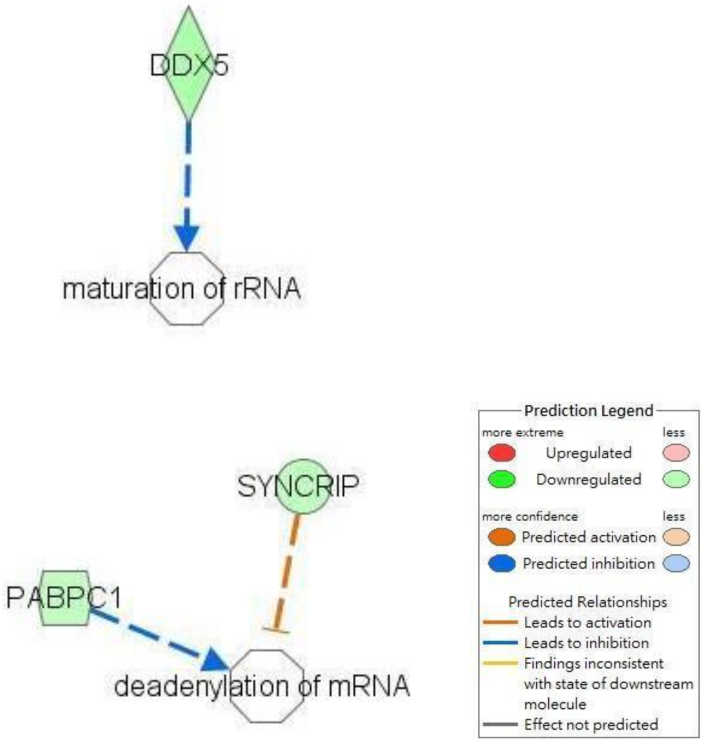

Figure S2.7. Causal Network Analysis of the RNA post-transcriptional modification with down-regulated genes.

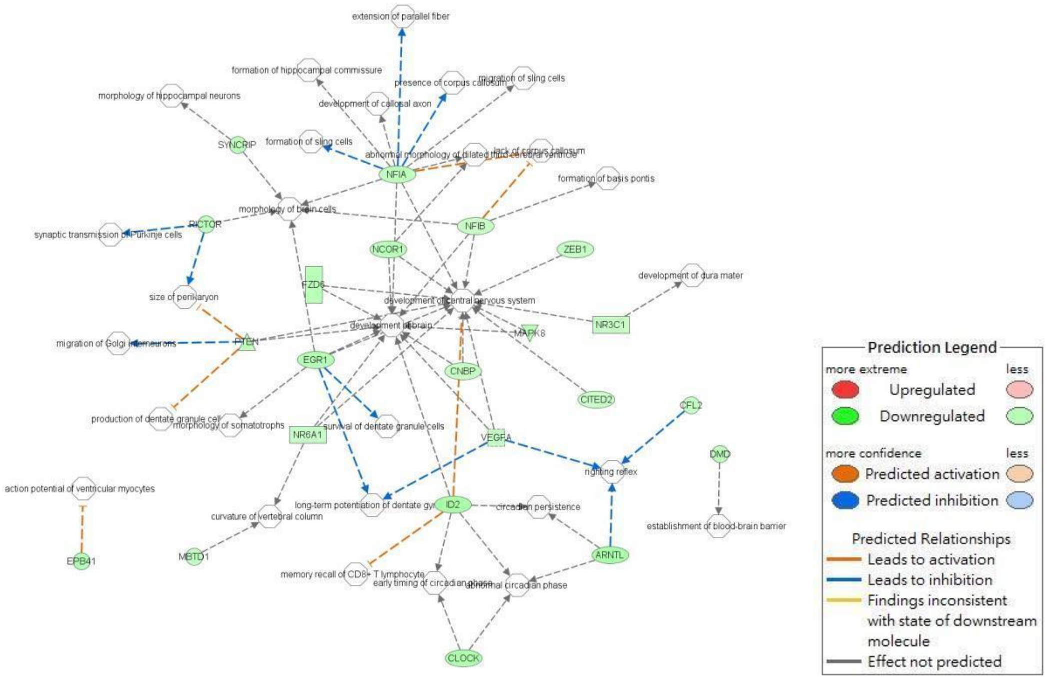

Figure S2.8. Causal Network Analysis of the nervous system development and function with down-regulated genes.

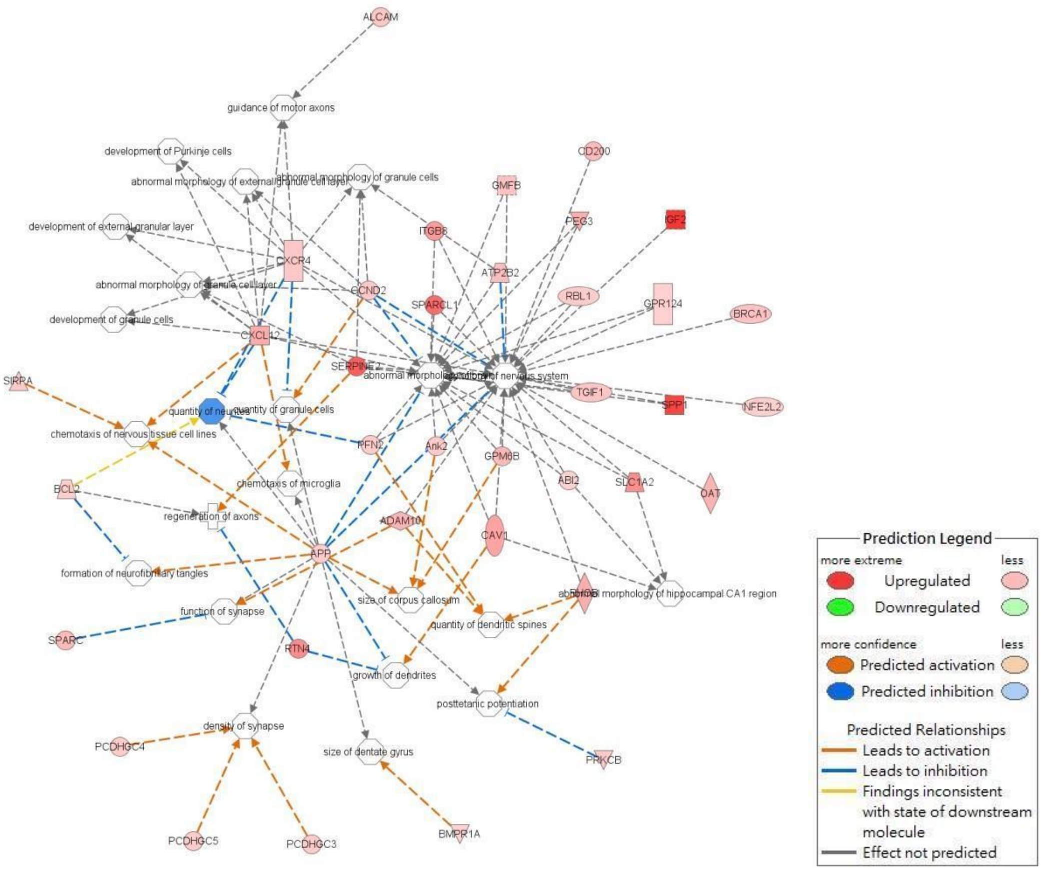

Figure S2.9. Causal Network Analysis of the nervous system development and function with up-regulated genes.

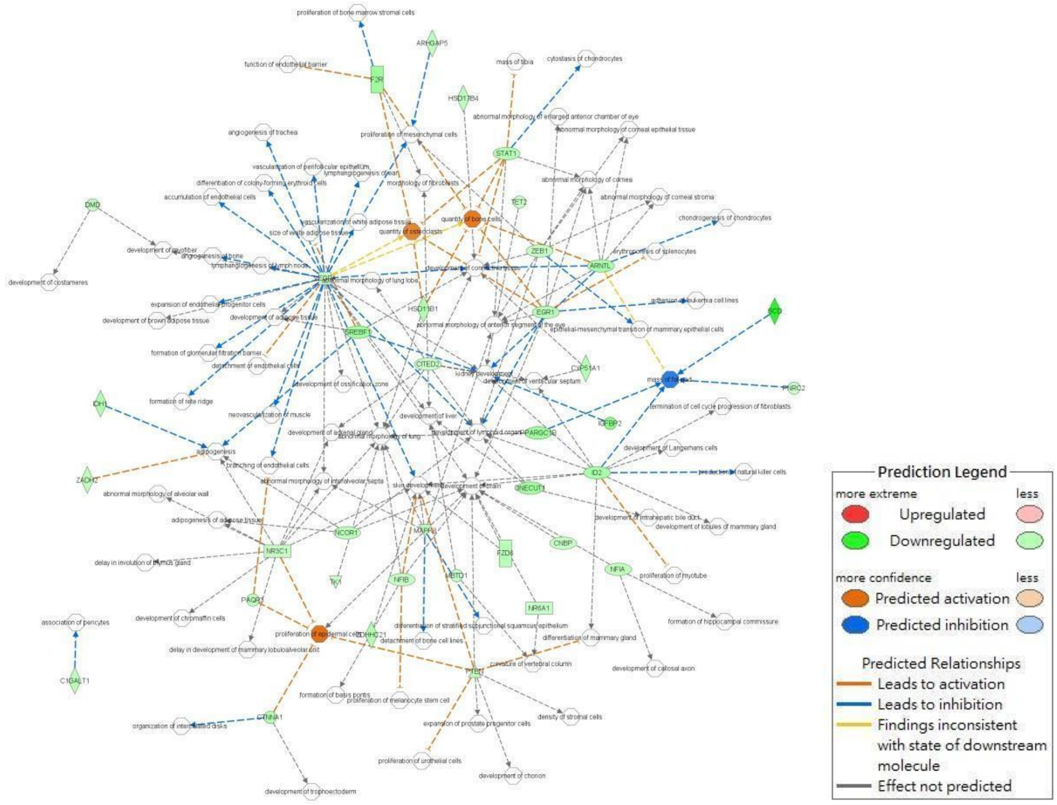

Figure S2.10. Causal Network Analysis of the tissue development with down-regulated genes.

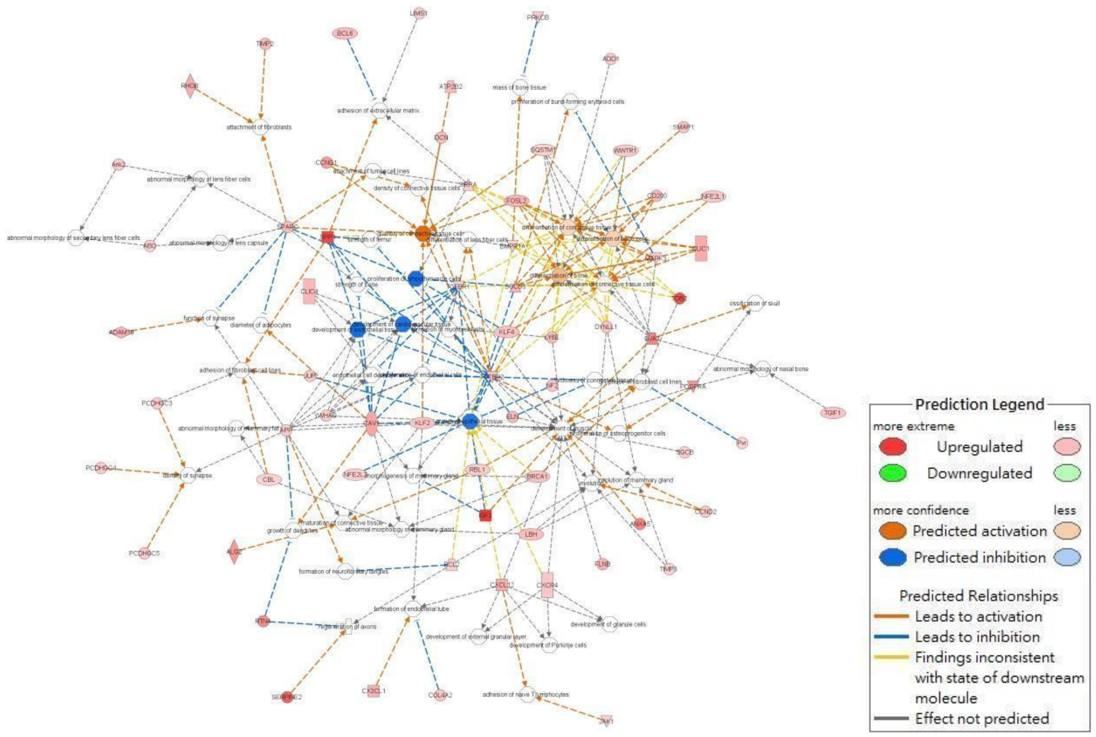

Figure S2.11. Causal Network Analysis of the tissue development with up-regulated genes.

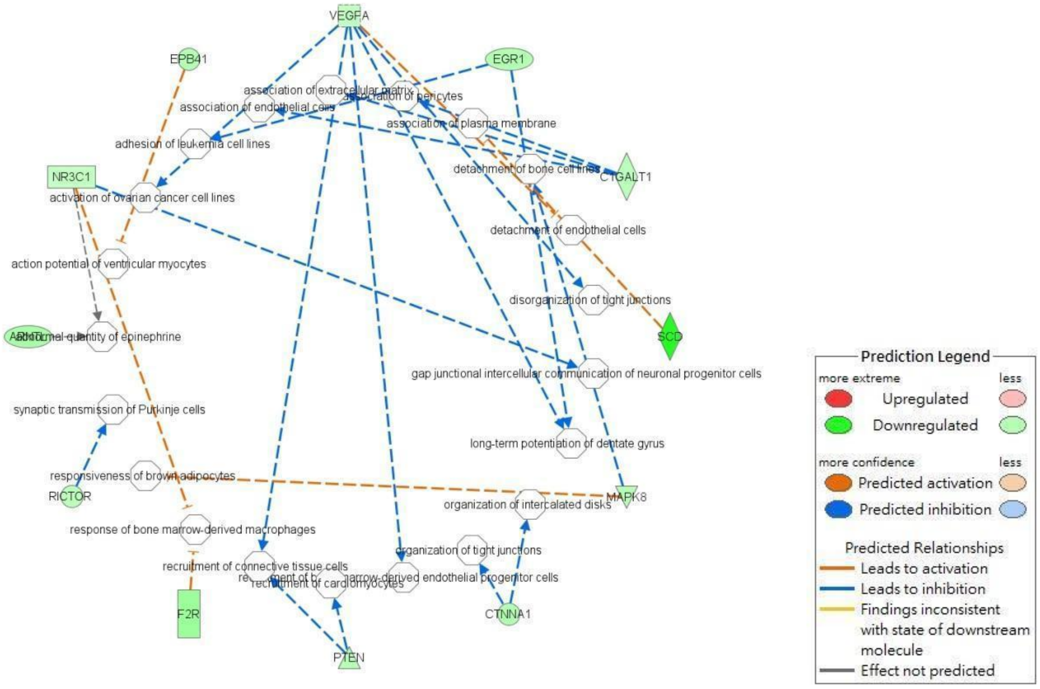

Figure S2.12. Causal Network Analysis of the cell-to-cell signaling and interaction with down-regulated genes.

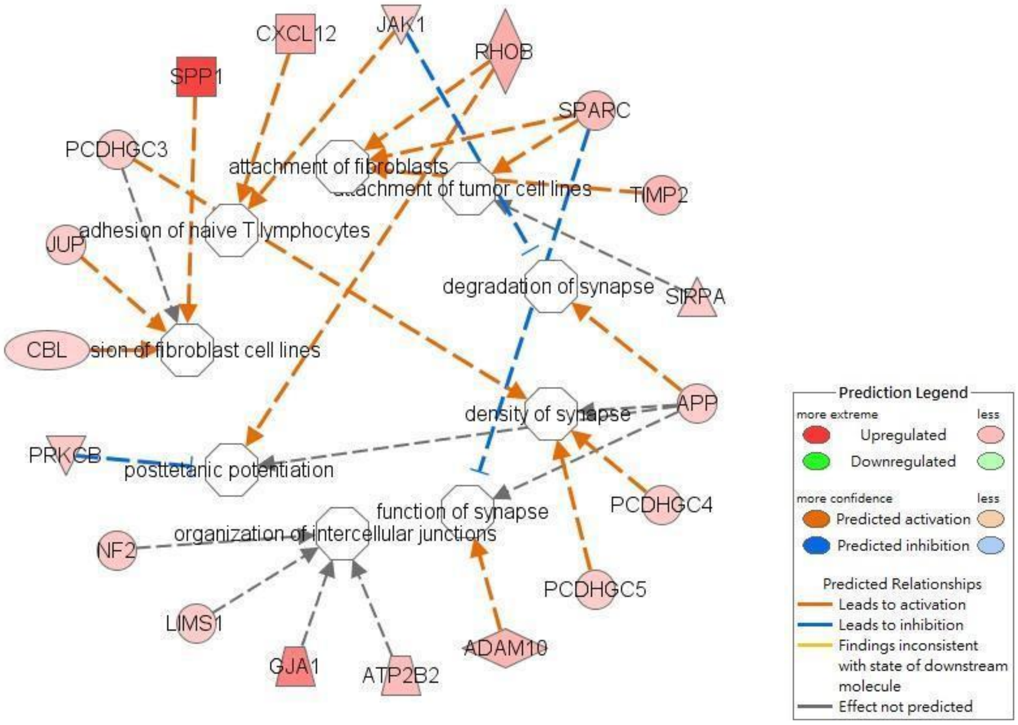

Figure S2.13. Causal Network Analysis of the ell-to-cell signaling and interaction with up-regulated genes.

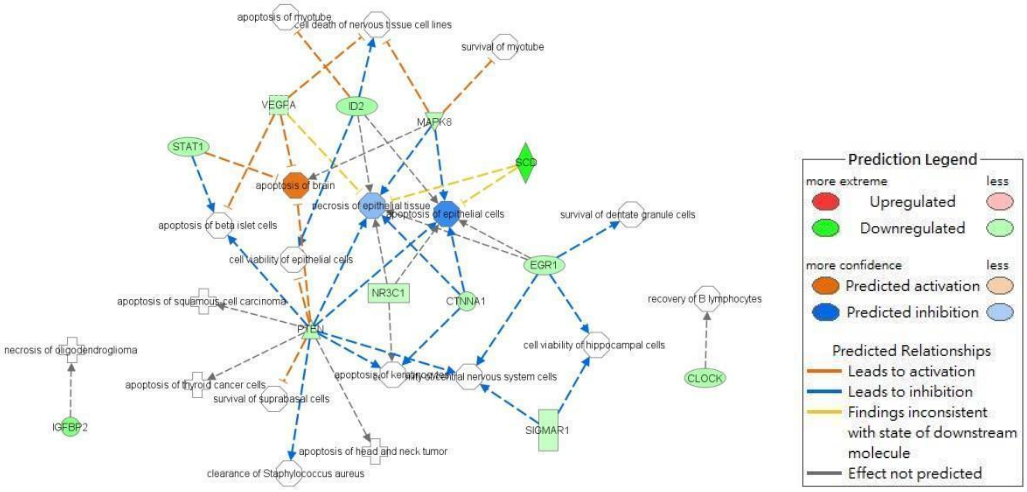

Figure S2.14. Causal Network Analysis of the cell death and survival with down-regulated genes.

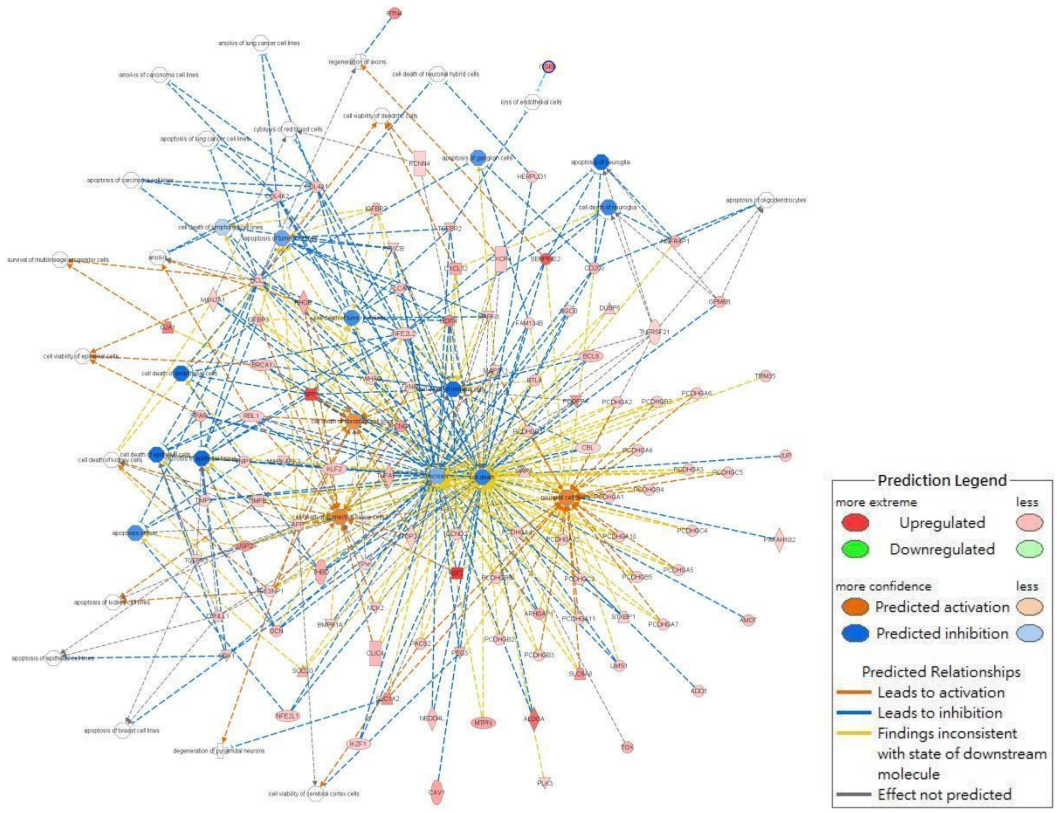

Figure S2.15. Causal Network Analysis of the cell death and survival with up-regulated genes.

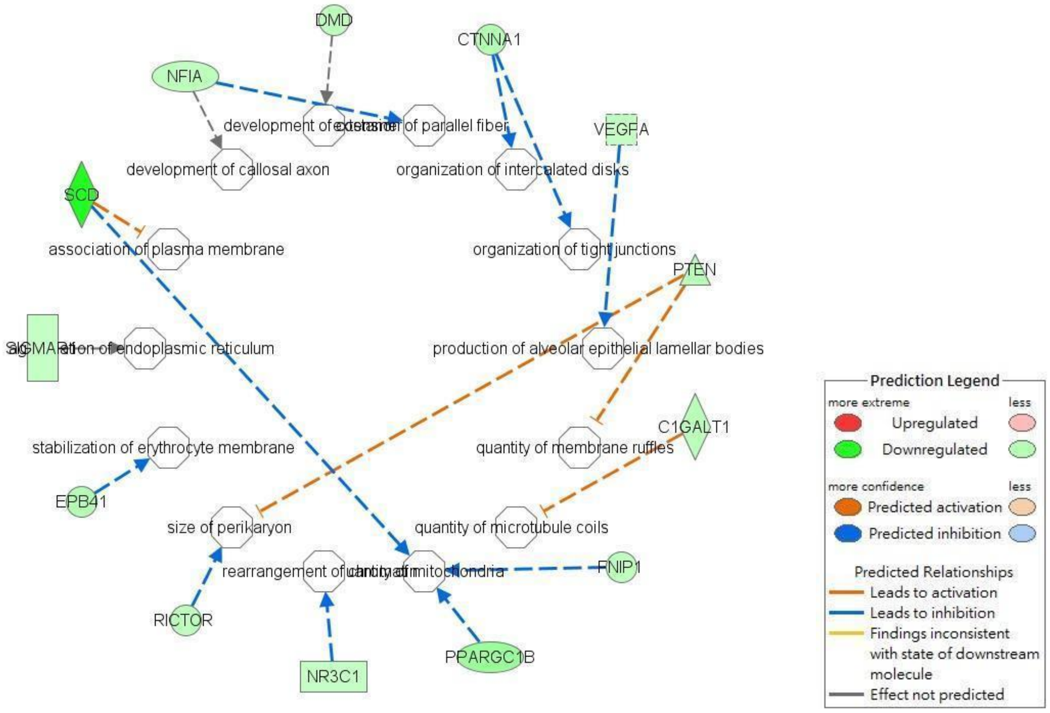

Figure S2.16. Causal Network Analysis of the cellular assembly and organization with down-regulated genes.

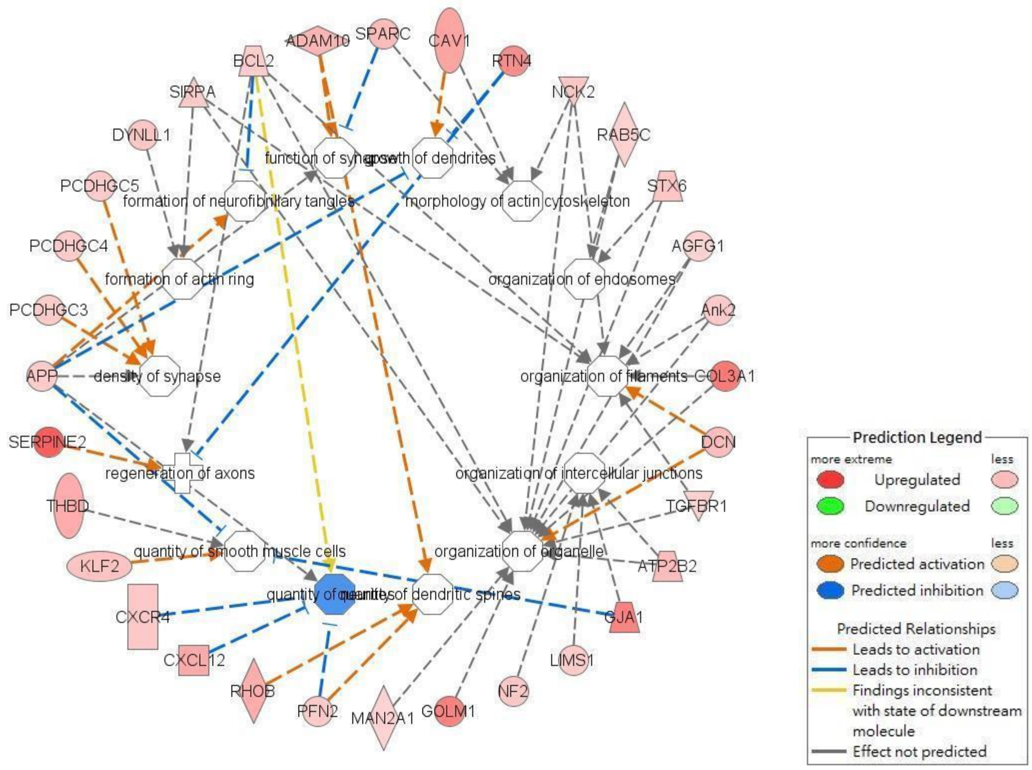

Figure S2.17. Causal Network Analysis of the cellular assembly and organization with up-regulated genes.

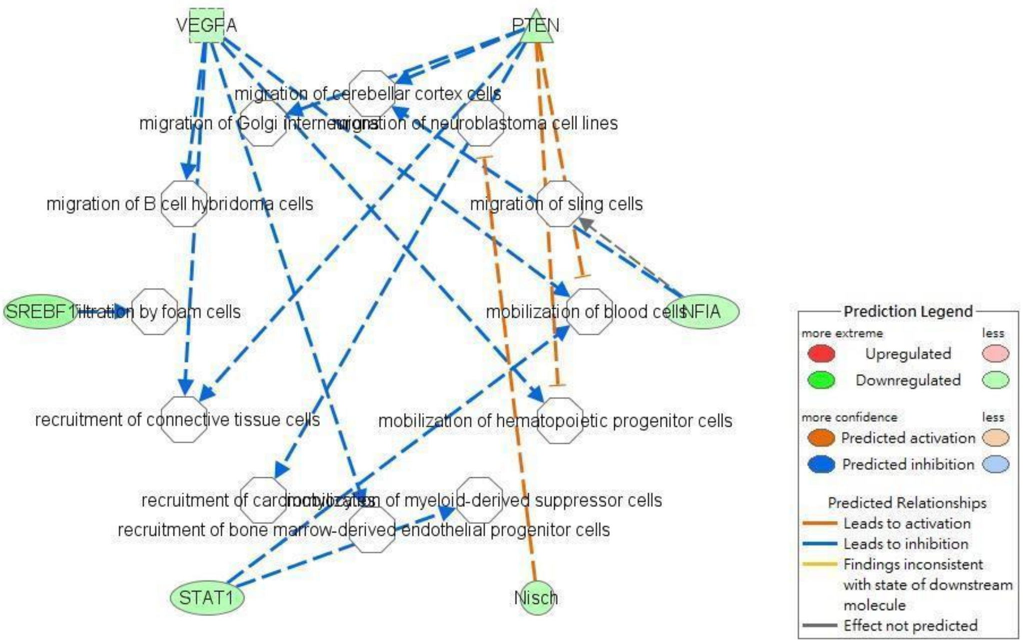

Figure S2.18. Causal Network Analysis of the cellular movement with down-regulated genes.

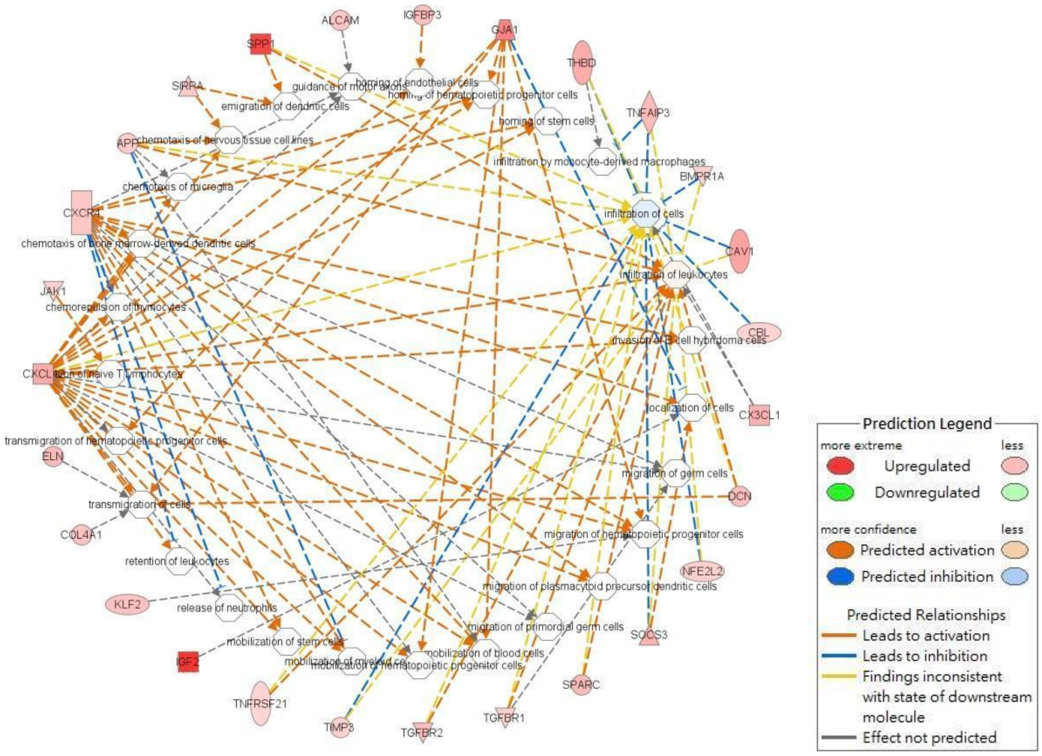

Figure S2.19. Causal Network Analysis of the cellular movement with up-regulated genes.

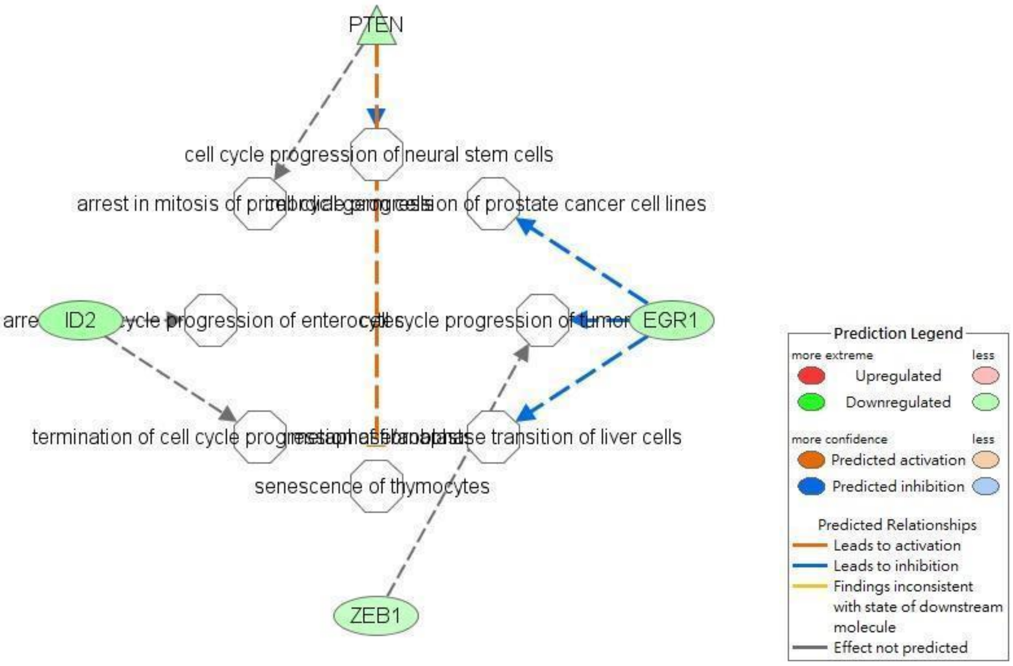

Figure S2.20. Causal Network Analysis of the cell cycle with down-regulated genes.

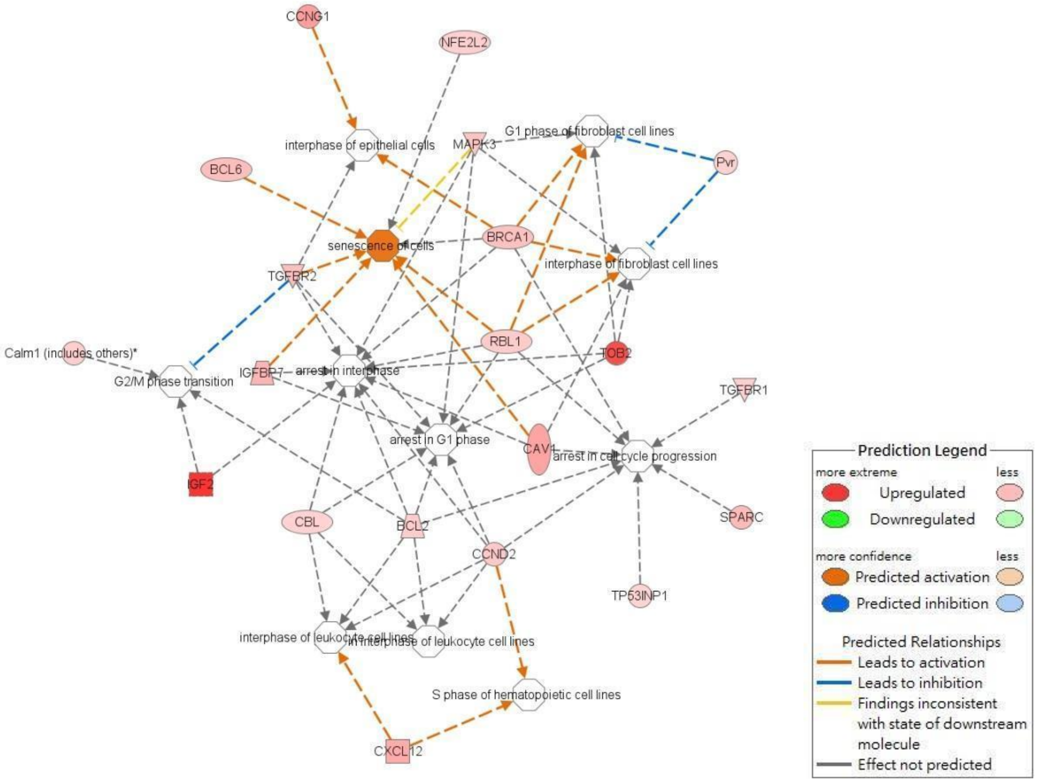

Figure S2.21. Causal Network Analysis of the cell cycle with up-regulated genes.

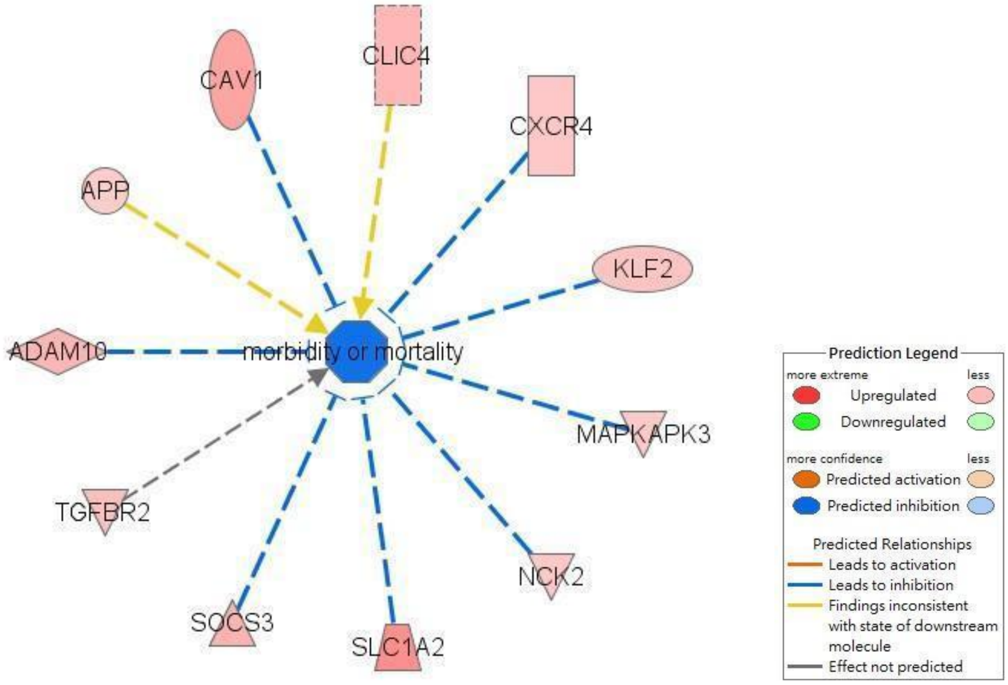

Figure S2.22. Causal Network Analysis of the organismal survival with up-regulated genes.

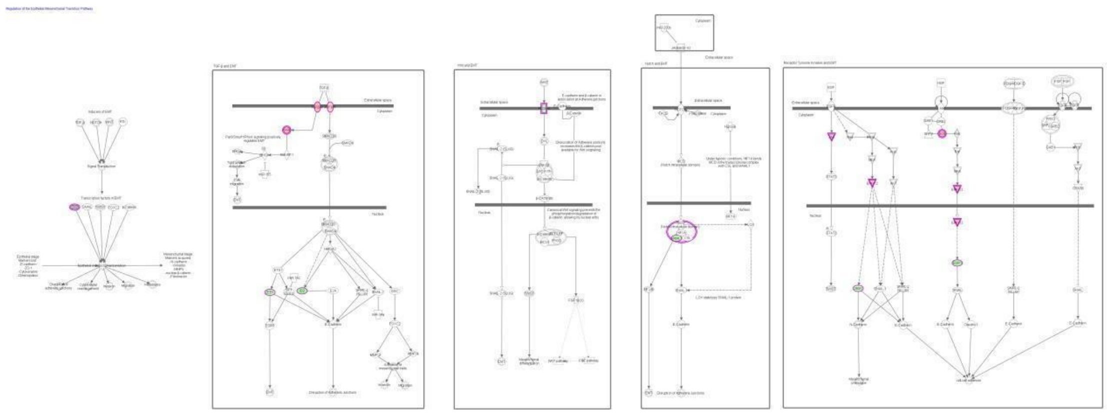

Figure S3.1. Ingenuity canonical pathways enriched in Regulation of the Epithelial-Mesenchymal Transition Pathway.

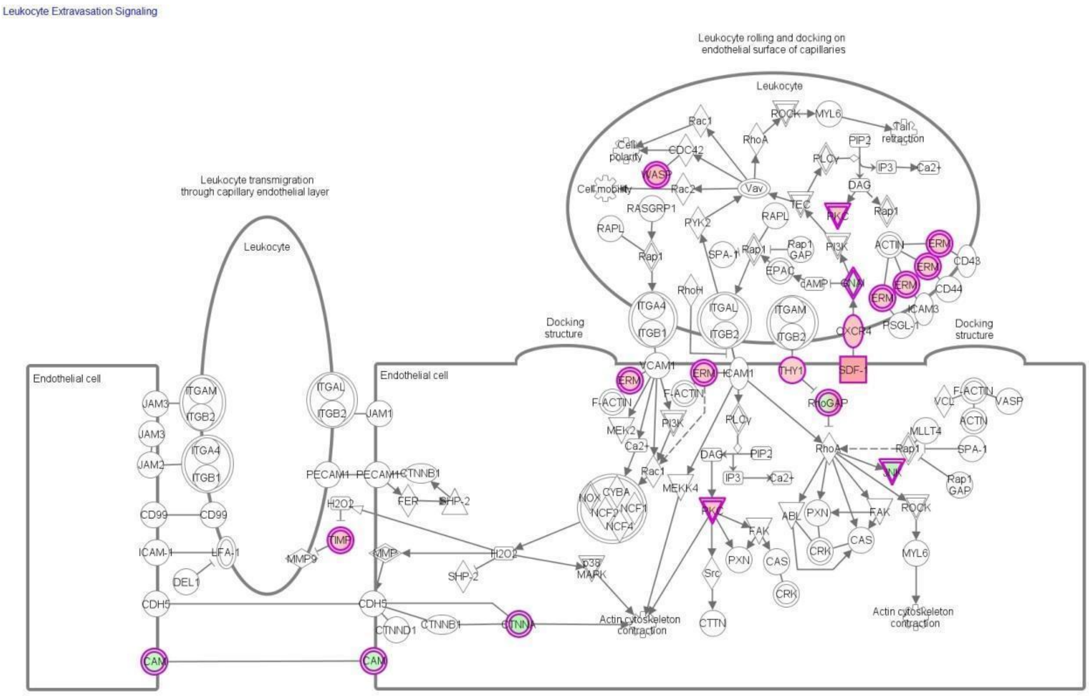

Figure S3.2. Ingenuity canonical pathways enriched in Leukocyte Extravasation Signaling.

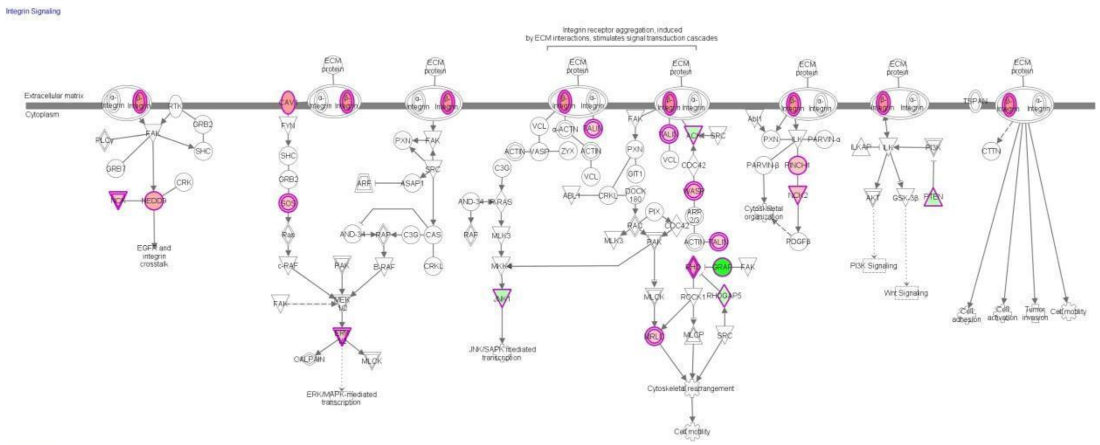

Figure S3.3. Ingenuity canonical pathways enriched in Integrin Signaling.

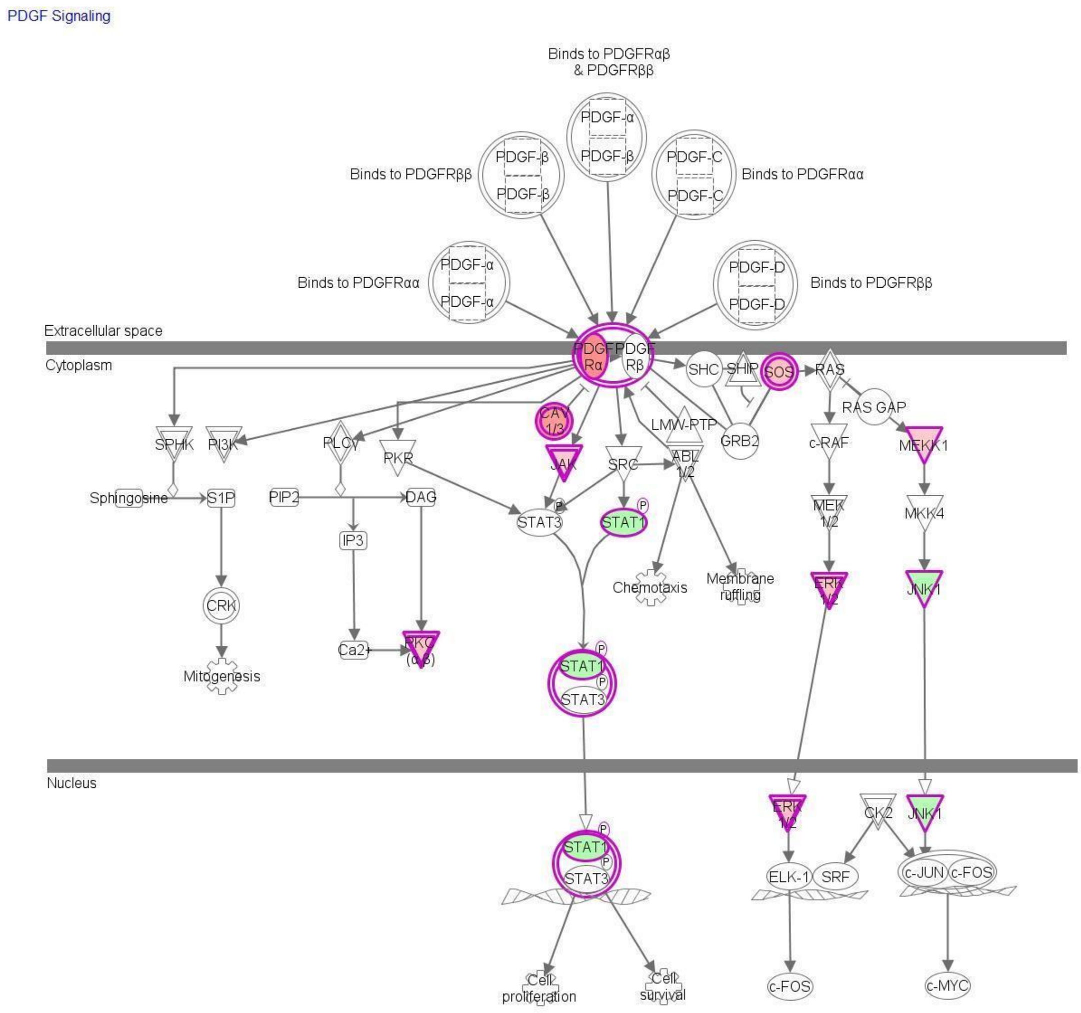

Figure S3.4. Ingenuity canonical pathways enriched in PDGF Signaling.

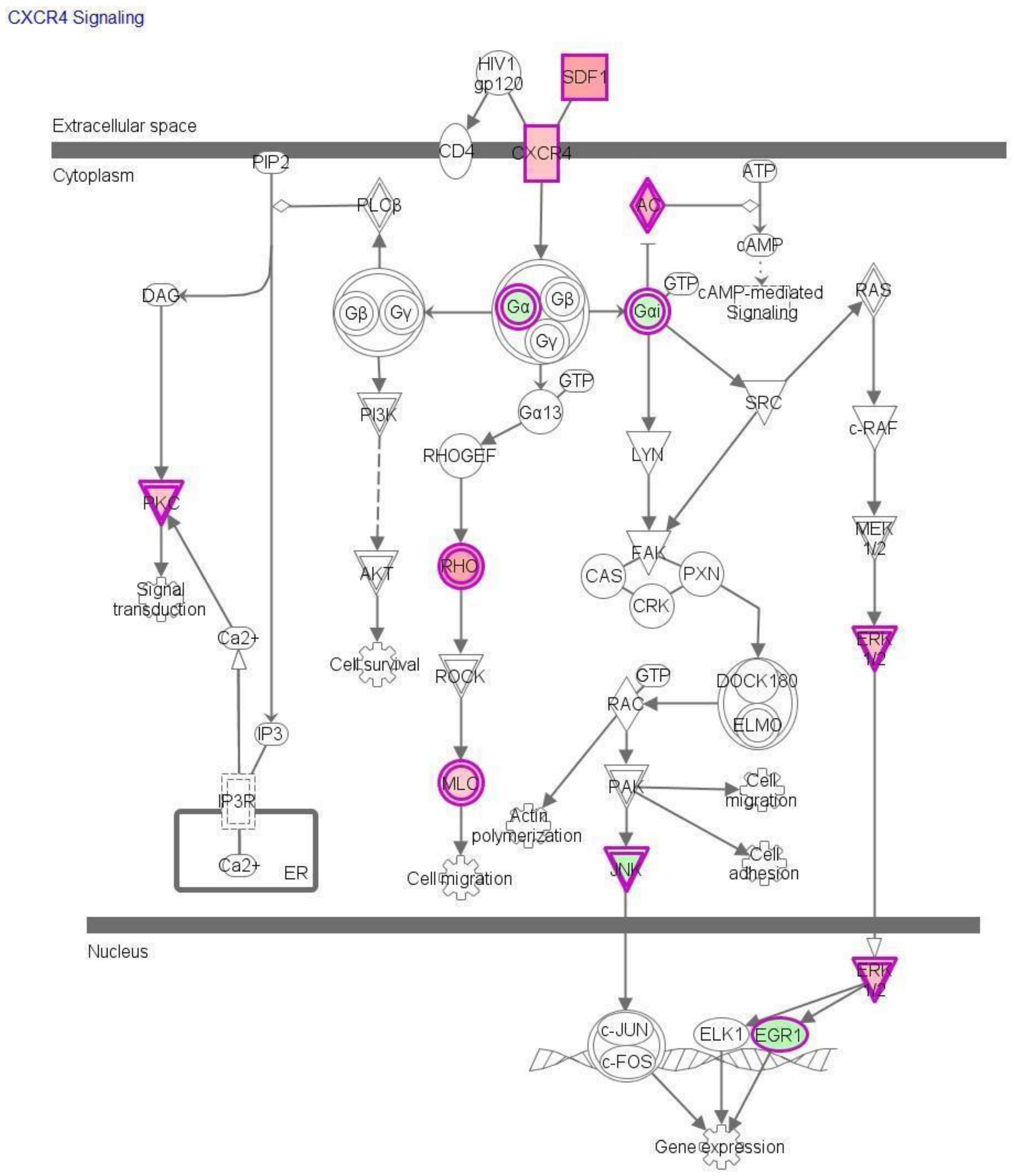

Figure S3.5. Ingenuity canonical pathways enriched in CXCR4 Signaling.

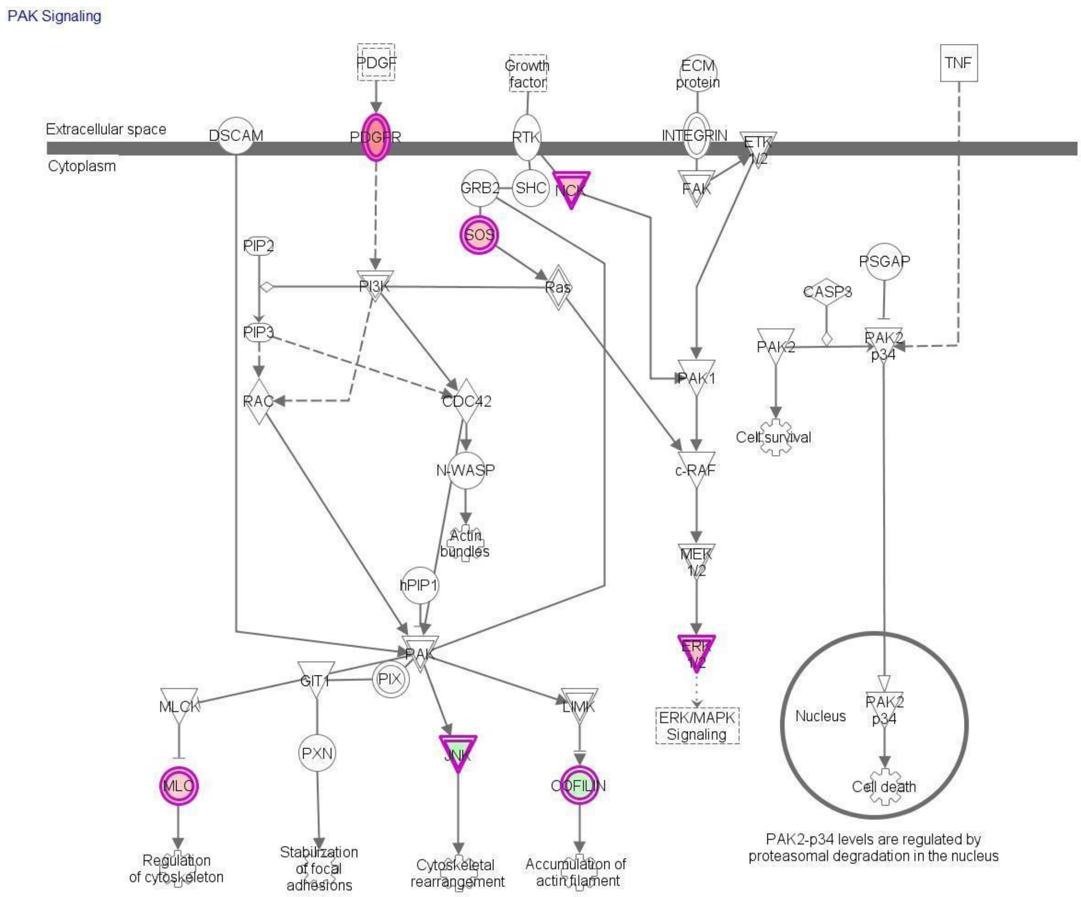

Figure S3.6. Ingenuity canonical pathways enriched in PAK Signaling.

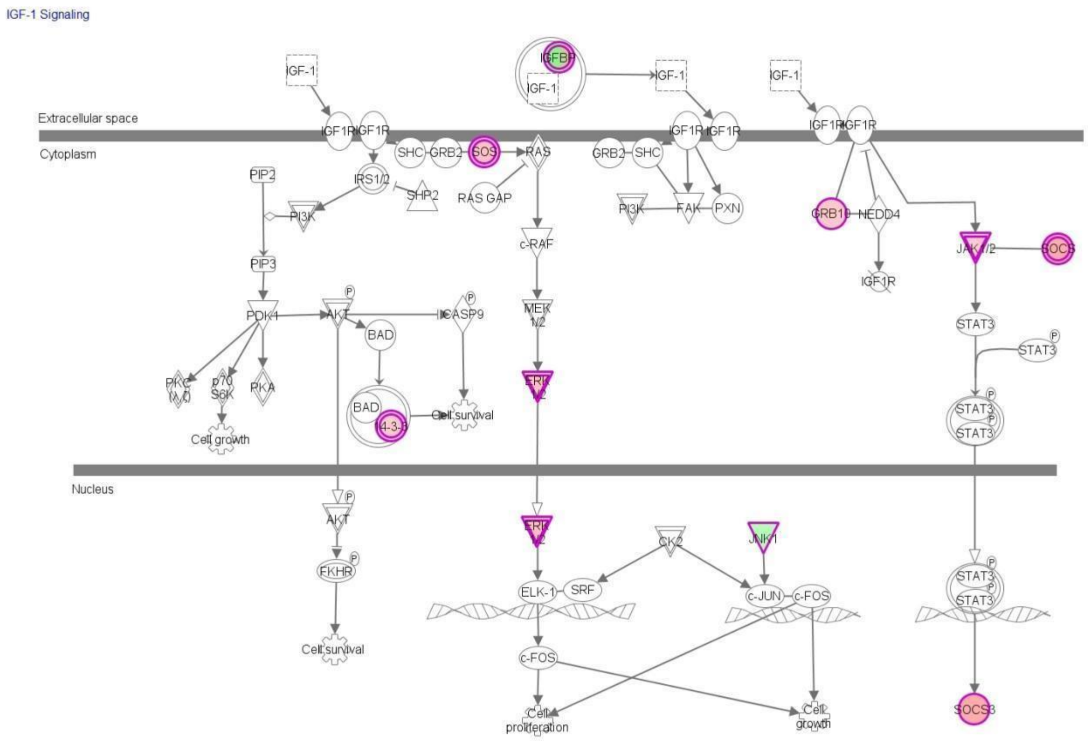

Figure S3.7. Ingenuity canonical pathways enriched in IGF-1 Signaling.

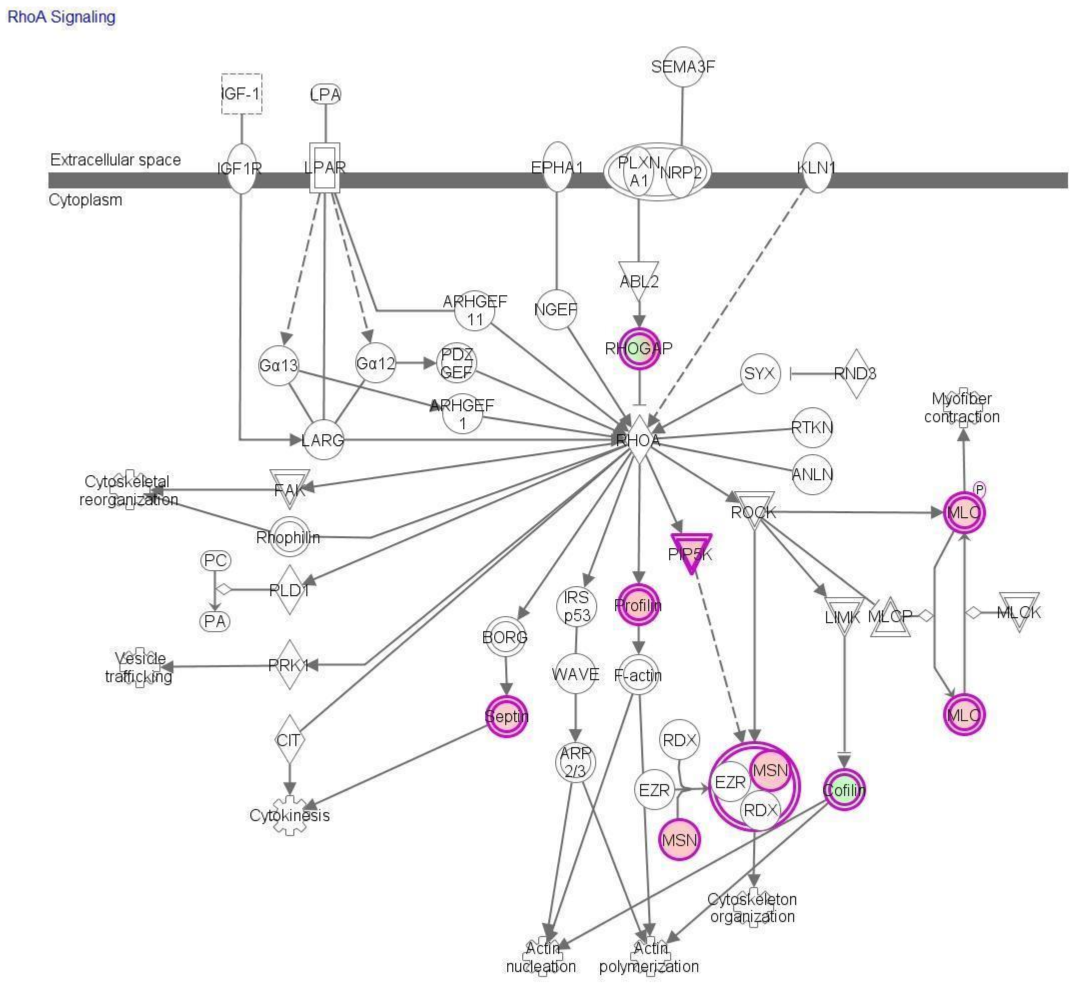

Figure S3.8. Ingenuity canonical pathways enriched in RhoA Signaling.

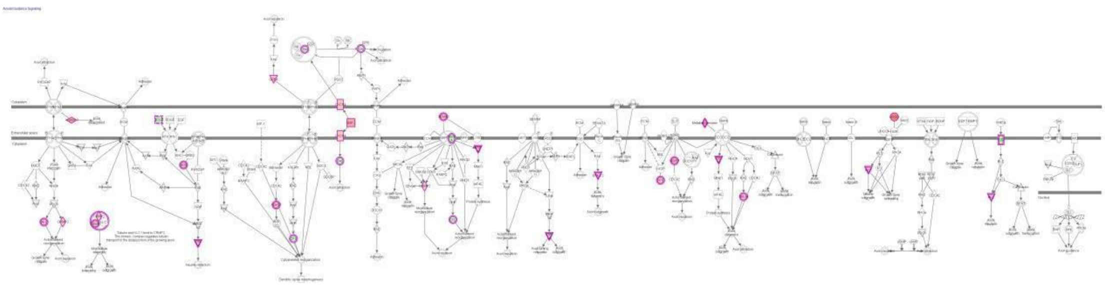

Figure S3.9. Ingenuity canonical pathways enriched in Axonal Guidance Signaling.

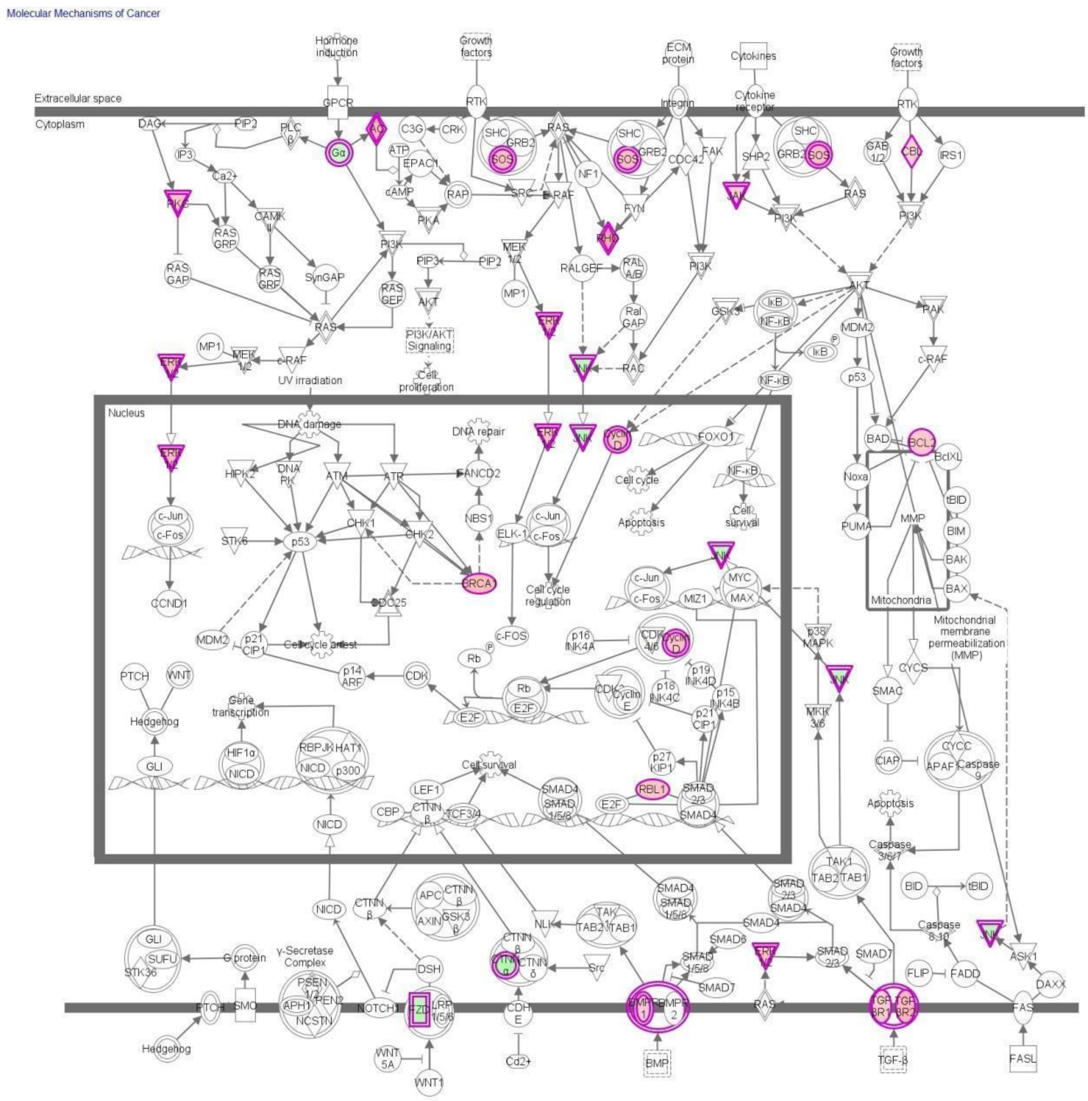

Figure S3.10. Ingenuity canonical pathways enriched in Molecular Mechanisms of Cancer.

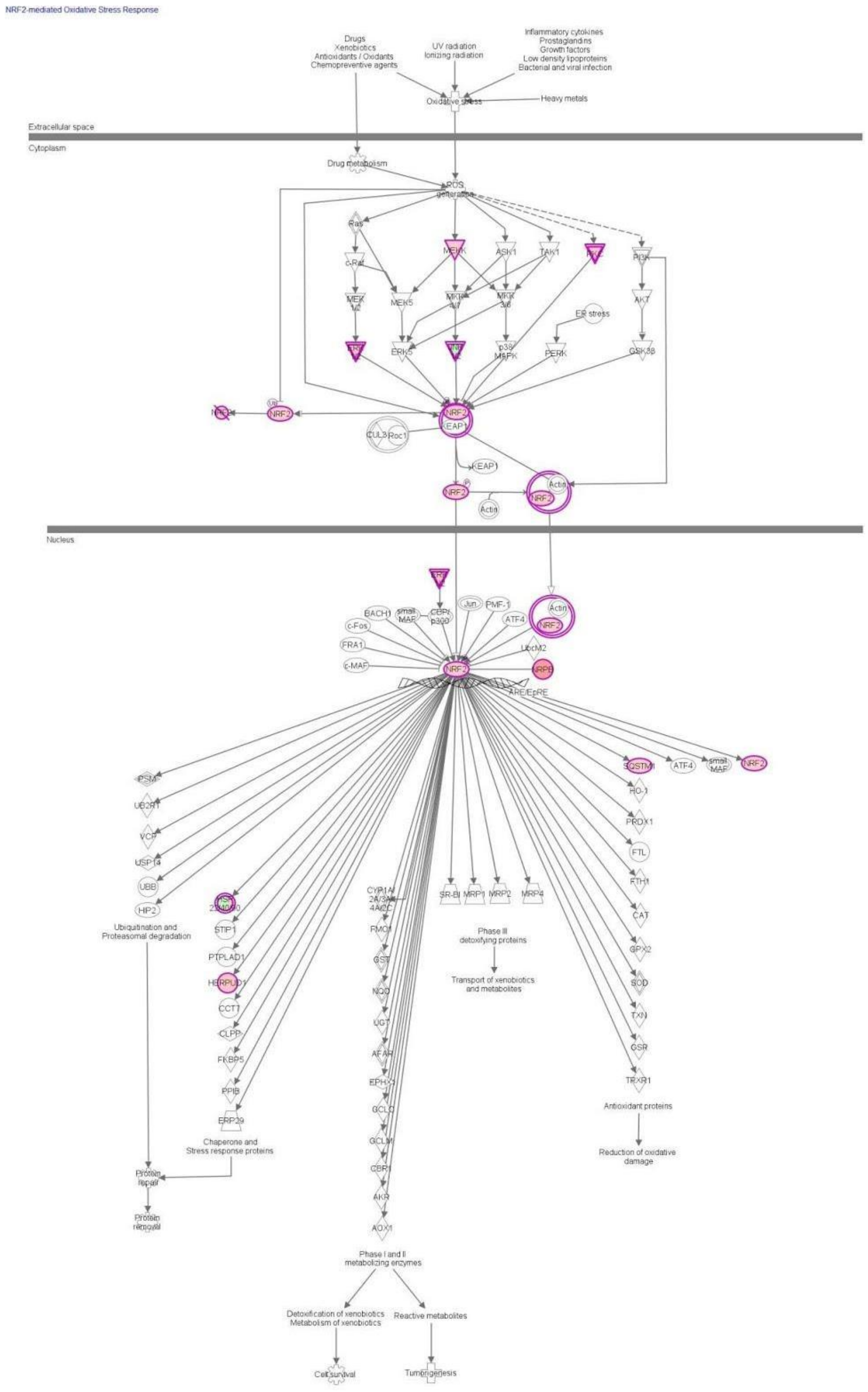

Figure S3.11. Ingenuity canonical pathways enriched in NRF2-mediated Oxidative Stress Response.

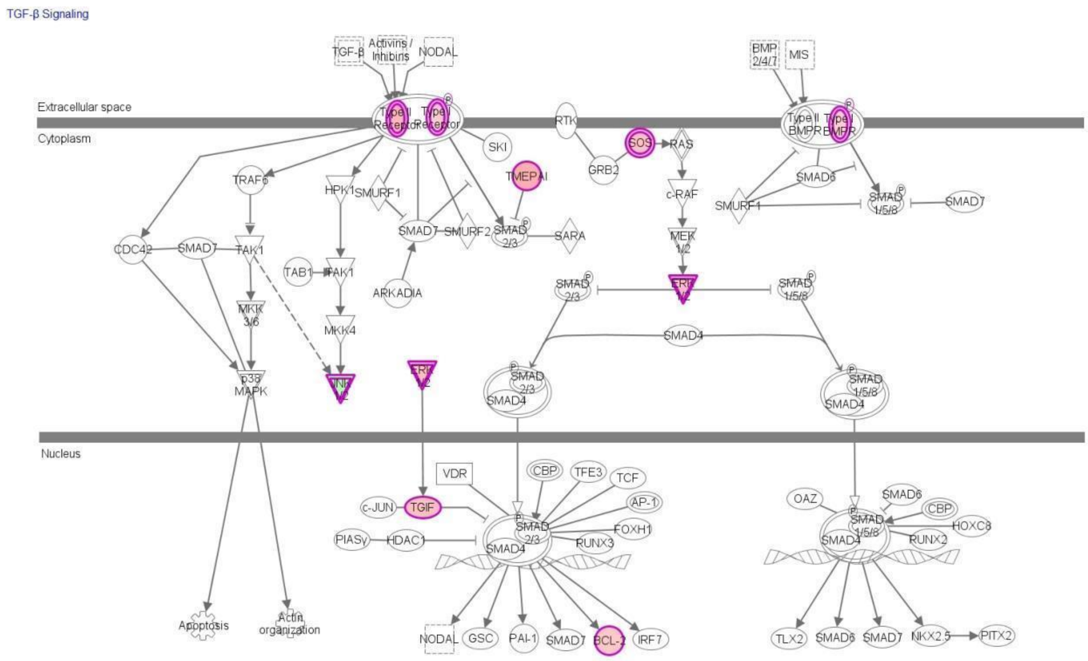

Figure S3.12. Ingenuity canonical pathways enriched in TGF-β Signaling.

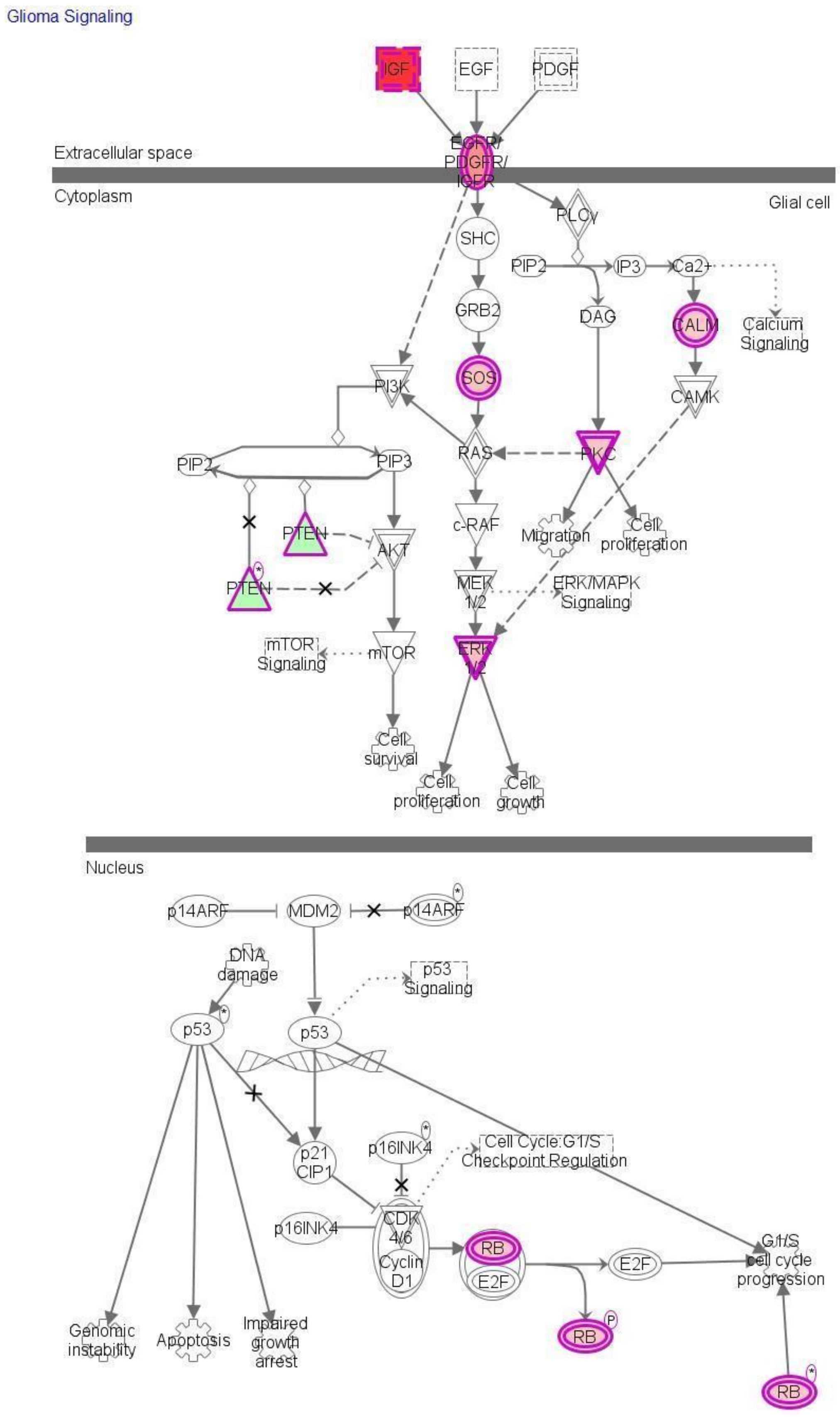

Figure S3.13. Ingenuity canonical pathways enriched in Glioma Signaling.

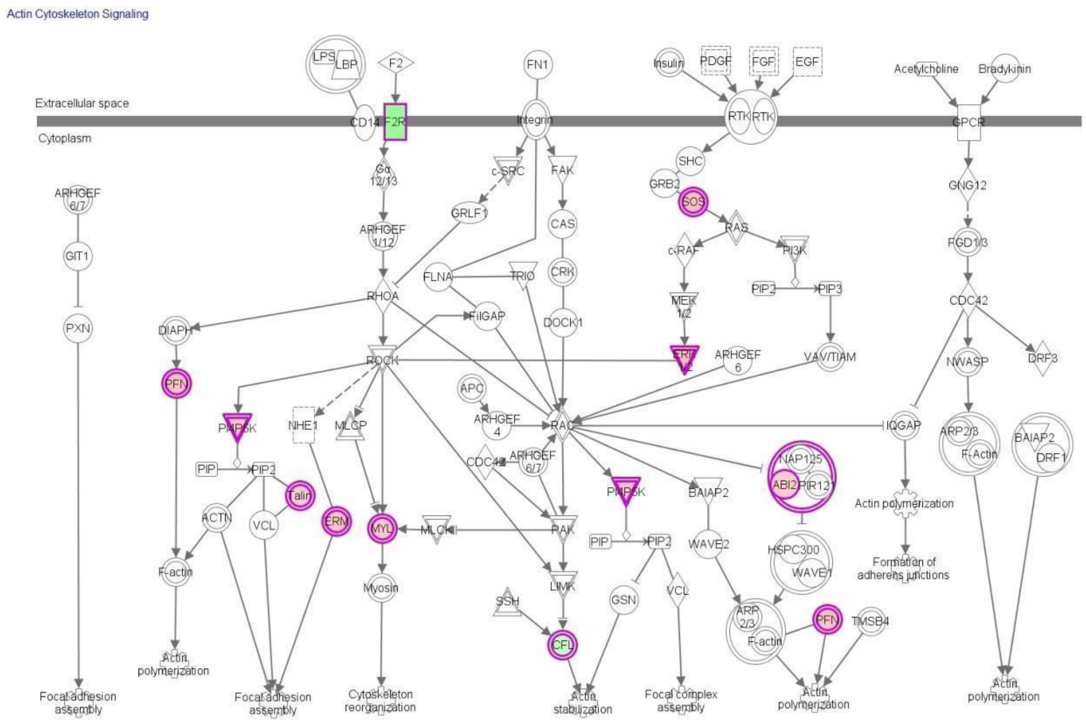

Figure S3.14. Ingenuity canonical pathways enriched in Actin Cytoskeleton Signaling.

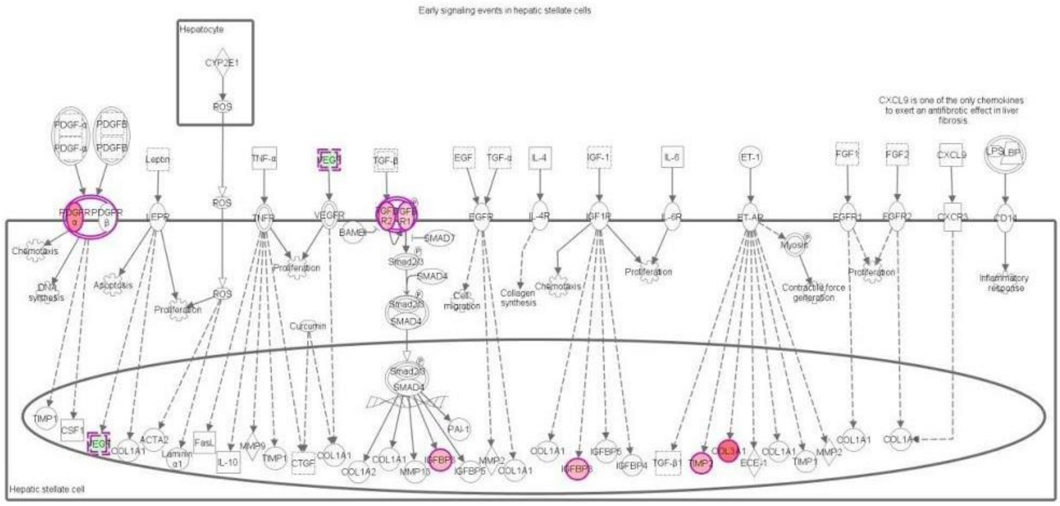

Figure S3.15. Ingenuity canonical pathways enriched in Hepatic Fibrosis.

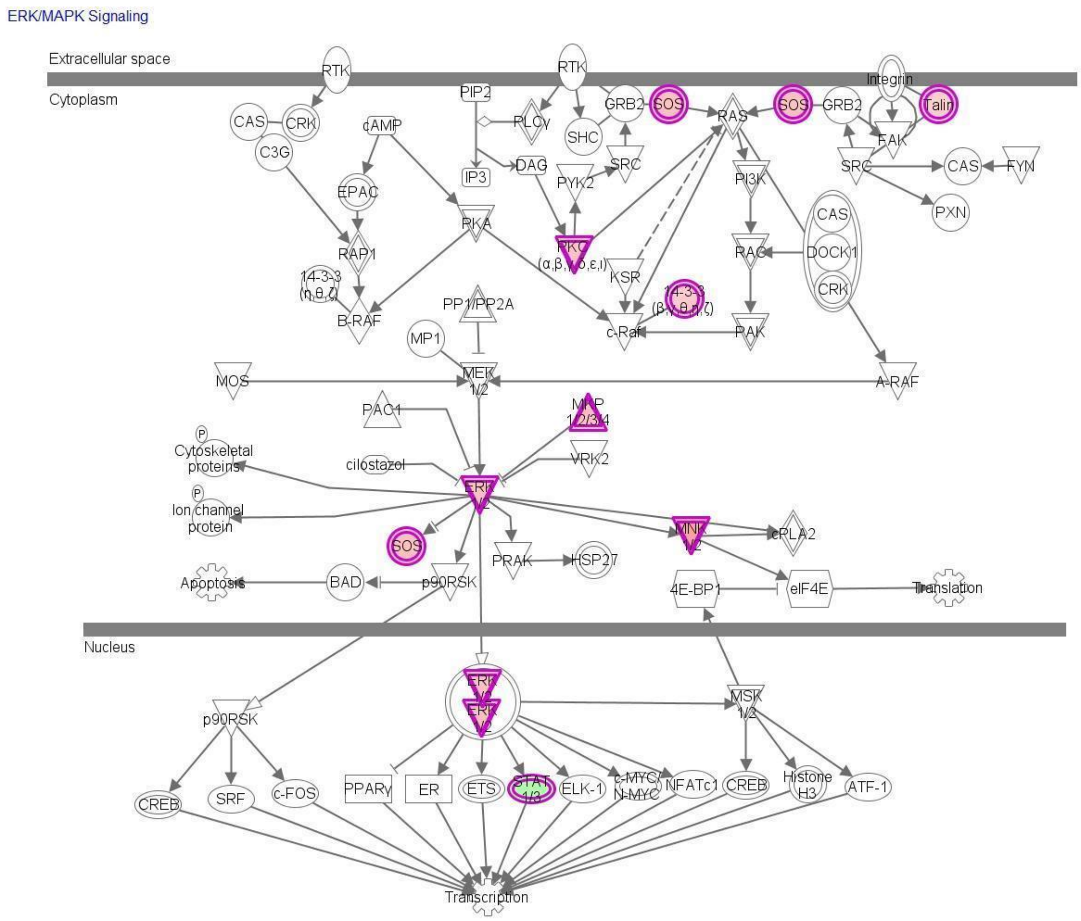

Figure S3.16. Ingenuity canonical pathways enriched in ERK/MAPK Signaling.

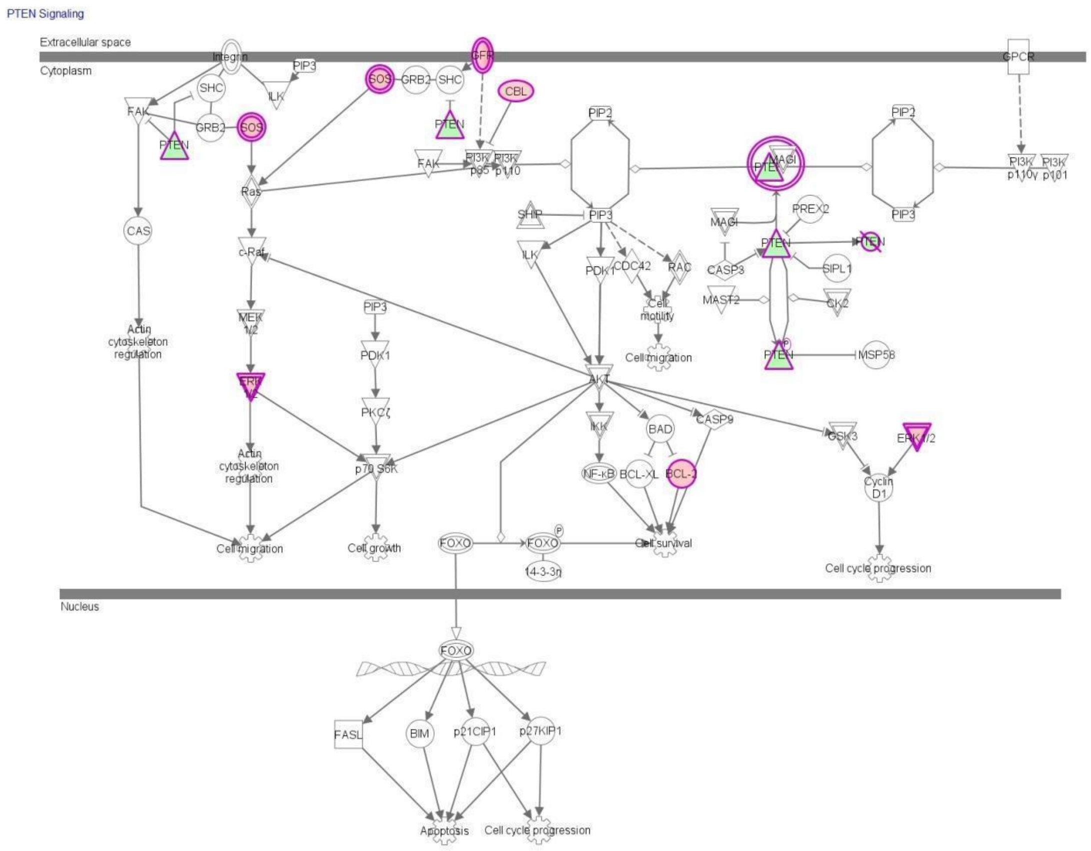

Figure S3.17. Ingenuity canonical pathways enriched in PTEN Signaling.

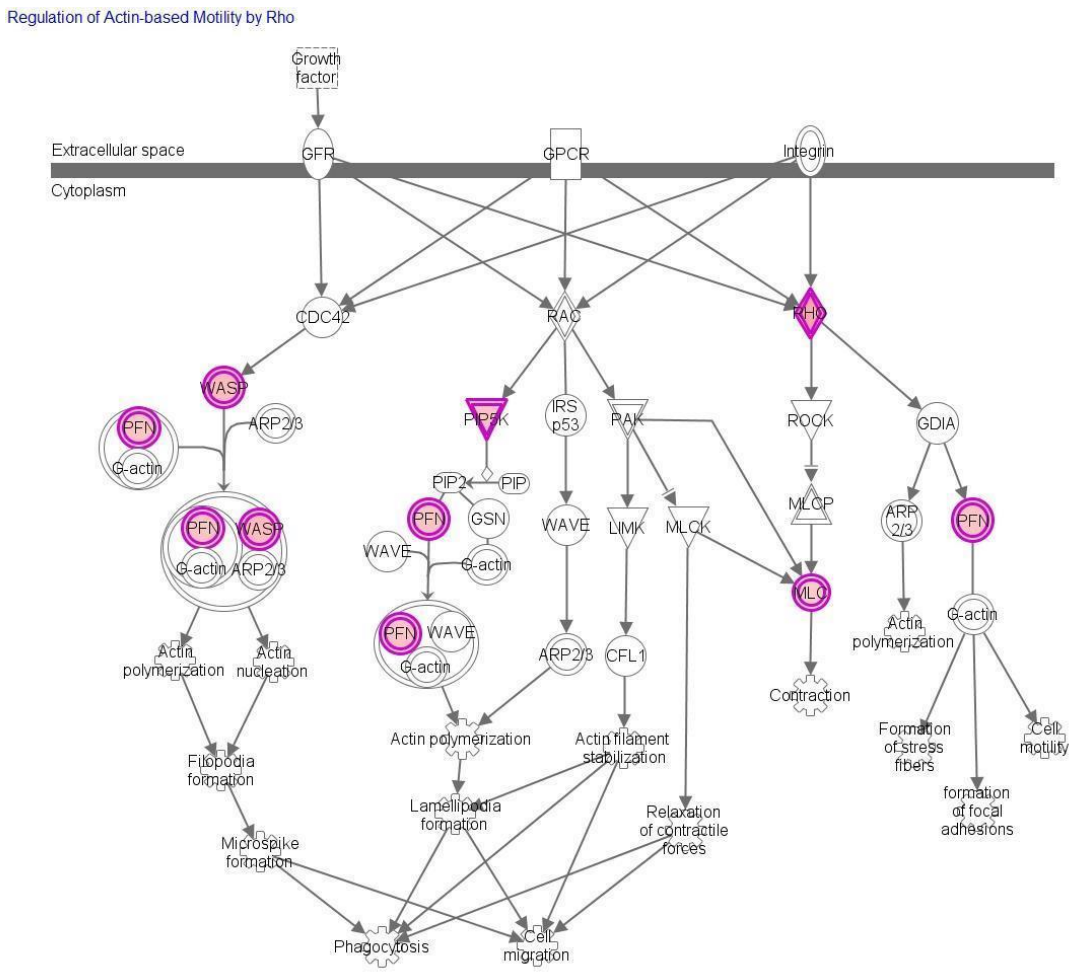

Figure S3.18. Ingenuity canonical pathways enriched in Regulation of Actin-based Motility by Rho.

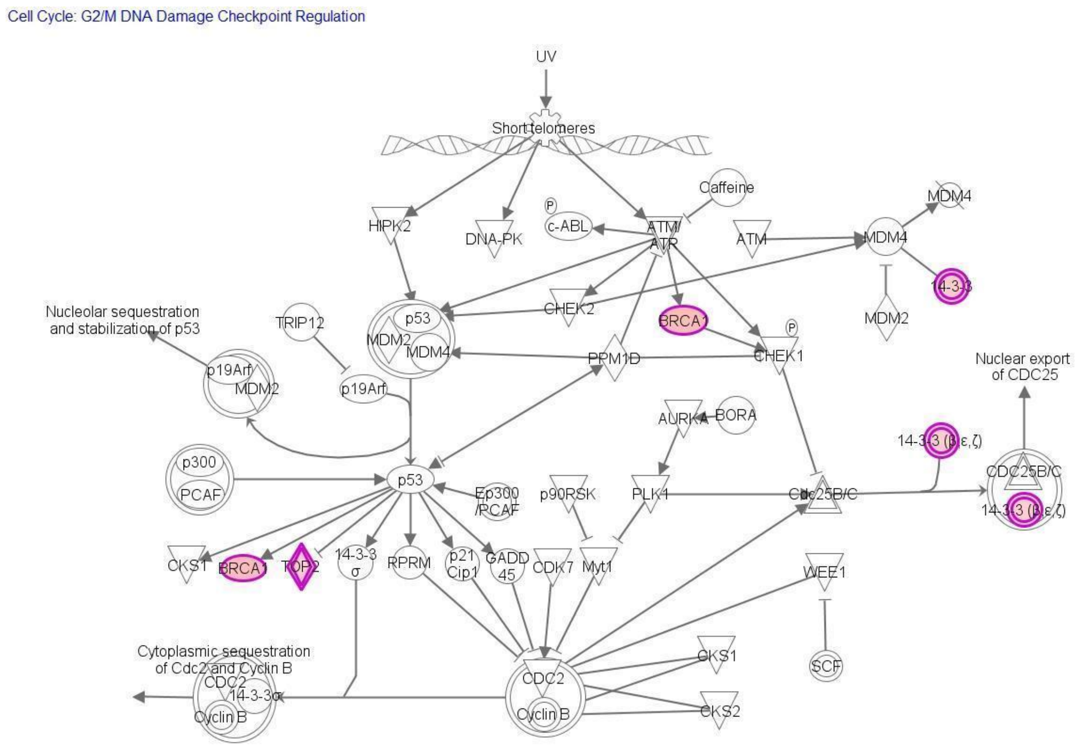

Figure S3.19. Ingenuity canonical pathways enriched in Cell Cycle: G2/M DNA Damage Checkpoint Regulation.
